# Comparative accuracy of biomarkers for the prediction of hospital-acquired acute kidney injury: a systematic review and meta-analysis

**DOI:** 10.1186/s13054-022-04223-6

**Published:** 2022-11-12

**Authors:** Heng-Chih Pan, Shao-Yu Yang, Terry Ting-Yu Chiou, Chih-Chung Shiao, Che-Hsiung Wu, Chun-Te Huang, Tsai-Jung Wang, Jui-Yi Chen, Hung-Wei Liao, Sheng-Yin Chen, Tao-Min Huang, Ya-Fei Yang, Hugo You-Hsien Lin, Ming-Jen Chan, Chiao-Yin Sun, Yih-Ting Chen, Yung-Chang Chen, Vin-Cent Wu

**Affiliations:** 1 Graduate Institute of Clinical Medicine, College of Medicine, National Taiwan University, Taipei, Taiwan; 2 Division of Nephrology, Department of Internal Medicine, Keelung Chang Gung Memorial Hospital, Keelung, Taiwan; 3 Chang Gung University College of Medicine, Taoyuan, Taiwan; 4 Community Medicine Research Center, Keelung Chang Gung Memorial Hospital, Keelung, Taiwan; 5 Division of Nephrology, Department of Internal Medicine, National Taiwan University Hospital, Room 1419, Clinical Research Building, 7 Chung-Shan South Road, Taipei, 100 Taiwan; 6 NSARF (National Taiwan University Hospital Study Group of ARF) and CAKS (Taiwan Consortium for Acute Kidney Injury and Renal Diseases), Taipei, Taiwan; 7 Division of Nephrology, Department of Internal Medicine, Camillian Saint Mary’s Hospital Luodong, Yilan, Taiwan; 8 Saint Mary’s Junior College of Medicine, Nursing and Management, Yilan, 26546 Taiwan; 9 Division of Nephrology, Taipei Tzu Chi Hospital, Buddhist Tzu Chi Medical Foundation, New Taipei City, Taiwan; 10 Department of Critical Care Medicine, Taichung Veterans General Hospital, Taichung, Taiwan; 11 Division of Nephrology, Department of Internal Medicine, Taichung Veterans General Hospital, Taichung, Taiwan; 12 Division of Nephrology, Department of Internal Medicine, Chi Mei Medical Center, Tainan, Taiwan; 13 Department of Health and Nutrition, ChiaNai University of Pharmacy and Science, Tainan, Taiwan; 14 Division of Nephrology, Department of Internal Medicine, Wan Fang Hospital, Taipei Medical University, Taipei, Taiwan; 15 Harvard T.H. Chan School of Public Health, Boston, MA USA; 16 Everan Hospital, Taichung, Taiwan; 17 China Medical University Hospital, Taichung, Taiwan; 18 Department of Internal Medicine, Kaohsiung Municipal Ta-Tung Hospital, Kaohsiung, Taiwan; 19 Division of Nephrology, Department of Internal Medicine, Kaohsiung Medical University Hospital, Kaohsiung, Taiwan; 20 Division of Nephrology, Department of Internal Medicine, Linkou Chang Gung Memorial Hospital, Taoyuan, Taiwan; 21 Institute of Public Health, National Yang Ming Chiao Tung University, Taipei, Taiwan

**Keywords:** Acute kidney injury, Biomarker, Critically ill patient, Neutrophil gelatinase-associated lipocalin

## Abstract

**Background:**

Several biomarkers have been proposed to predict the occurrence of acute kidney injury (AKI); however, their efficacy varies between different trials. The aim of this study was to compare the predictive performance of different candidate biomarkers for AKI.

**Methods:**

In this systematic review, we searched PubMed, Medline, Embase, and the Cochrane Library for papers published up to August 15, 2022. We selected all studies of adults (> 18 years) that reported the predictive performance of damage biomarkers (neutrophil gelatinase-associated lipocalin (NGAL), kidney injury molecule-1 (KIM-1), liver-type fatty acid-binding protein (L-FABP)), inflammatory biomarker (interleukin-18 (IL-18)), and stress biomarker (tissue inhibitor of metalloproteinases-2 × insulin-like growth factor-binding protein-7 (TIMP-2 × IGFBP-7)) for the occurrence of AKI. We performed pairwise meta-analyses to calculate odds ratios (ORs) and 95% confidence intervals (CIs) individually. Hierarchical summary receiver operating characteristic curves (HSROCs) were used to summarize the pooled test performance, and the Grading of Recommendations, Assessment, Development and Evaluations criteria were used to appraise the quality of evidence.

**Results:**

We identified 242 published relevant studies from 1,803 screened abstracts, of which 110 studies with 38,725 patients were included in this meta-analysis. Urinary NGAL/creatinine (diagnostic odds ratio [DOR] 16.2, 95% CI 10.1–25.9), urinary NGAL (DOR 13.8, 95% CI 10.2–18.8), and serum NGAL (DOR 12.6, 95% CI 9.3–17.3) had the best diagnostic accuracy for the risk of AKI. In subgroup analyses, urinary NGAL, urinary NGAL/creatinine, and serum NGAL had better diagnostic accuracy for AKI than urinary IL-18 in non-critically ill patients. However, all of the biomarkers had similar diagnostic accuracy in critically ill patients. In the setting of medical and non-sepsis patients, urinary NGAL had better predictive performance than urinary IL-18, urinary L-FABP, and urinary TIMP-2 × IGFBP-7: 0.3. In the surgical patients, urinary NGAL/creatinine and urinary KIM-1 had the best diagnostic accuracy. The HSROC values of urinary NGAL/creatinine, urinary NGAL, and serum NGAL were 91.4%, 85.2%, and 84.7%, respectively.

**Conclusions:**

Biomarkers containing NGAL had the best predictive accuracy for the occurrence of AKI, regardless of whether or not the values were adjusted by urinary creatinine, and especially in medically treated patients. However, the predictive performance of urinary NGAL was limited in surgical patients, and urinary NGAL/creatinine seemed to be the most accurate biomarkers in these patients. All of the biomarkers had similar predictive performance in critically ill patients.

*Trial registration* CRD42020207883, October 06, 2020.

**Supplementary Information:**

The online version contains supplementary material available at 10.1186/s13054-022-04223-6.

## Background

Acute kidney injury (AKI) is associated with a higher risk of chronic kidney disease (CKD), end-stage renal disease (ESRD), and long-term adverse cardiovascular effects [1, 2]. Due to the lack of effective treatment for impaired kidney function, the best strategy in clinical practice is to identify AKI as early as possible, reverse its cause, and even improve the sequelae. In the past decades, several serum creatinine (SCr)-based classification systems have been proposed to define AKI [3]. Serum creatinine has traditionally served as a surrogate of kidney function, despite its limitations as a diagnostic surrogate of AKI [4]. The limitations of SCr include a lack of steady-state conditions in critically ill patients, and that the determinants of SCr (rate of production, apparent volume of distribution, and rate of elimination) are variable. Therefore, there is an unmet need for other objective measures to help detect AKI in a timely manner. The role of several biomarkers in the early prediction or risk assessment of AKI has been proposed, including kidney tubular damage markers (e.g., neutrophil gelatinase-associated lipocalin (NGAL), kidney injury molecule-1 (KIM-1), liver-type fatty acid-binding protein (L-FABP)) [5–9], inflammation markers (e.g., interleukin-18 (IL-18)) [6, 10, 11], and stress markers (e.g., tissue inhibitor of metalloproteinases-2 and insulin-like growth factor-binding protein-7 (TIMP-2 ×  IGFBP-7)). The ADQI expert group suggests that routine clinical assessments should be combined with stress, damage, and functional biomarkers to stratify risk, discriminate etiologies, assess severity, plan management, and predict the duration and recovery of AKI [12]. In addition, previous meta-analyses including patients with various clinical scenarios have suggested that these biomarkers hold promise as practical tools in the early prediction of AKI [5, 13–17]. However, few studies have compared the diagnostic accuracy of these AKI biomarkers, and systematic assessments of the quality of evidence, which can provide updated information for clinical guidelines, are lacking. Therefore, the aim of this study was to compare the reported predictive accuracy of AKI biomarkers in various clinical settings and appraise the quality of evidence using a pairwise meta-analysis. The findings of this study may be used to update guidelines and recommendations.

## Methods

### Search strategy and selection criteria

We conducted this pairwise meta-analysis according to the Preferred Reporting Items of Systematic Reviews and Meta-Analyses (PRISMA) statement [18] and used Cochrane methods [19]. We prospectively submitted the systematic review protocol for registration on PROSPERO [CRD42020207883].

### Data sources and search strategy

The primary outcome was incident AKI. Electronic searches were performed on PubMed (Ovid), Medline, Embase, and Cochrane library from inception to August 15, 2022 (Additional file 1: Appendix). We screened references by titles and abstracts and included related studies for further analysis. Reference lists of related studies, systematic reviews, and meta-analyses were manually examined to identify any possible publications relevant to our analysis. Both abstracts and full papers were selected for quality assessment and data synthesis.

### Inclusion and exclusion criteria

The inclusion criteria were as follows: (1) clinical studies that included participants over 18 years of age and of any ethnic origin or sex; (2) studies that reported candidate AKI biomarkers including NGAL, KIM-1, L-FABP, IL-18, and TIMP-2 × IGFBP-7; and (3) studies that assessed the occurrence of incident AKI. The exclusion criteria were as follows: (1) studies including patients who had previously received dialysis; (2) studies including pregnant or lactating patients; (3) letters, conference or case reports; and (4) studies that lacked data on sensitivity or specificity of biomarkers to predict the occurrence of AKI. Only regular full papers were selected for quality assessment and data synthesis. We contacted the authors of abstracts for further detailed information, if available.

### Study selection and data extraction

Six investigators (Heng-Chih Pan, Terry Ting-Yu Chiou, Chih-Chung Shiao, Che-Hsiung Wu, Hugo You-Hsien Lin, and Ming-Jen Chan) independently reviewed the search results and identified eligible studies. Any resulting discrepancies were resolved by discussion with a seventh investigator (Vin-Cent Wu). All relevant data were independently extracted from the included studies by eight investigators (Heng-Chih Pan, Chih-Chung Shiao, Terry Ting-Yu Chiou, Yih-Ting Chen, Chun-Te Huang, Ya-Fei Yang, Shu-Chen Yu, and Zi-Ming Chen) according to a standardized form. Extracted data included study characteristics (lead author, publication year, population setting, biomarkers, study endpoint, sample size, events, timing of measurements) and participants’ baseline data (mean age (years), gender (%), comorbidities, severity of illness). When available, odds ratios and 95% confidence intervals (CIs) from cohort or case-controlled studies were extracted. Other a priori determined parameters included the type of intensive care unit (ICU) setting (surgical/mixed or medical), criteria used to diagnose AKI and severe AKI, cohort size, and the presence of sepsis. Any disagreements were resolved by discussion with the investigators (Heng-Chih Pan and Vin-Cent Wu).

### Quality assessment

The Quality Assessment of Diagnostic Accuracy Studies-2 (QUADAS-2) tool was used to assess the quality of each included study [20, 21]. The following 4 domains were assessed: patient selection, index test, reference standard, and flow and timing. Any disagreements in the quality assessment were resolved by discussion and consensus [15].

### Pre-specified subgroup analysis

We hypothesized that the following factors could have high impacts on patient outcomes observed among different studies: clinical setting (ICU/non-ICU), patient population (surgical versus mixed/medical), whether the studies only included patients with sepsis or not and different AKI criteria (risk, injury, failure, loss, ESRD (RIFLE); Acute Kidney Injury Network (AKIN); Kidney Disease: Improving Global Outcomes (KDIGO)).

### Data synthesis and statistical analysis

A 2 by 2 table reporting the patient number of true positive, false positive, true negative, and false negative findings for the cutoff point given by the included studies was used to generate sensitivity, specificity, and diagnostic odds ratio (DOR) for each study. The sensitivity, specificity, and DOR for all of the included studies were combined using a bivariate model. DOR was defined as the endpoint of primary interest in this study because it combines the strengths of sensitivity and specificity with the advantage of accuracy as a single indicator [22]. The sensitivity and specificity were defined as the endpoints of secondary interest in the study. The diagnostic performance for AKI among the 12 different biomarkers was compared using a bivariate model in which the type of biomarker was treated as a categorical covariate. Hierarchical summary receiver operating characteristic curves (HSROCs), which consider the threshold effect [23], were used to illustrate the overall diagnostic performance for each biomarker. The analysis was further stratified by the following pre-specified subgroups: surgical versus mixed/medical patients, ICU/non-ICU patients, sepsis/non-sepsis patients, and different AKI criteria (RIFLE/AKIN/KDIGO). In the subgroup analysis, biomarkers only reported in 1 study could not be compared and were therefore excluded. Potential publication bias was assessed visually using funnel plots. A two-sided *P* value < 0.05 was considered statistically significant. The bivariate model was conducted using SAS version 9.4 (SAS Institute, Cary, NC) with the “METADAS” macro (version 1.3) which is recommended by the Cochrane Diagnostic Test Accuracy Working Group. The HSROC analysis and funnel plots were performed using R software version 3.6.3 with the “meta4diag” package (version 2.0.8) based on Bayesian inference.

## Results

### Search results and study characteristics

The study selection process is summarized in Additional file 1: Appendix. A total of 23,882 articles were identified through the electronic search, and after excluding duplicate and non-relevant articles, the titles and abstracts of the remaining 1803 articles were screened. A total of 242 studies were eligible for full-text review, of which 110 studies including 38,725 patients reported data on the occurrence of AKI with any one of the biomarkers of interest and were included in the meta-analysis [24–133]. The details of the included studies and population characteristics as well as definitions used for the diagnosis of AKI are shown in Tables 1 and 2.

**Table 1:** Characteristics of included comparative studies

No	Study (year)	Population setting	Biomarker	Endpoint	AKI criteria	UOC	Total patient	No AKI (%)	AKI (%)	AKI severity	Timing of measurement
1	Qian et al. 2019 [24]	Patients who underwent cardiac surgery	Urinary NGAL Urinary Klotho	AKI within post-op 7 days	AKIN	No	91	58 (63.7)	33 (36.3)	AKI stage 1, 2, 3	Post-op 0, 2, 4 h
2	Prowle et al. 2015 [25]	Cardiopulmonary bypass, ICU patients	Urinary NGAL Urinary NGAL/Cr Urinary L-FABP	AKI within post-op 5 days	RIFLE	No	93	68 (73.1)	25 (26.9)	AKI stage R, I, F	Pre-op, and post-op 24 h
3	Lei et al. 2018 [26]	Decompensated cirrhosis	Urinary NGAL Urinary KIM-1 Serum CyC Serum Cr	AKI within 7 days	KDIGO	Yes	150	82 (54.7)	68 (45.3)	AKI stage 1, 2, 3	At hospital admission
4	van Wolfswinkel et al. 2016 [27]	Patients with imported falciparum malaria	Urinary NGAL Urinary KIM-1 Serum NGAL	AKI within 7 days	KDIGO	No	39	33 (84.6)	6 (15.4)	AKI stage 1, 2, 3	At hospital admission
5	Srisawat et al. 2015 [28]	Hospitalized patients with Leptospirosis	Urinary NGAL Serum NGAL	AKI within 7 days	KDIGO	No	113	71 (62.8)	42 (37.2)	AKI stage ≥ 1	At hospital admission
6	Zeng et al. 2014 [29]	Major surgery	Urinary NGAL Urinary L-FABP	Post-op AKI within 2 days	AKIN	No	197	160 (81.2)	37 (18.8)	AKI stage ≥ 1	Pre-op, and post-op 0, 4, 12 h and 1, 2, 7, 14 days
7	Aydoğdu et al. 2013 [30]	Critically ill patients with and without sepsis	Urinary NGAL Urinary CyC Serum CyC	AKI within 7 days	RIFLE	Yes	151	88 (58.3)	63 (41.7)	AKI stage R, I, F	Every day since ICU admission to the day of AKI
8	Liu et al. 2013 [31]	Cardiac surgery	Urinary NGAL Urinary L-FABP	Post-op AKI within 3 days	AKIN	No	109	83 (76.1)	26 (23.9)	AKI stage 1, 2, 3	Pre-op, and post-op 0, 2 h
9	Wagener et al. 2011 [32]	Orthotopic liver transplantation	Urinary NGAL/Cr	Post-op AKI within 7 days	RIFLE	No	92	55 (59.8)	37 (40.2)	AKI stage ≥ R	Pre-op, post-op 3, 18, 24 h
10	Makris et al. 2009 [33]	Critically ill multiple trauma patients	Urinary NGAL	AKI within 3 days	RIFLE	No	31	20 (64.5)	11 (35.5)	AKI stage R, I, F	At ICU admission and post-admission 24, 48 h
11	Constantin et al. 2010 [34]	Critically ill patients	Serum NGAL	AKI at ICU admission	RIFLE	No	88	36 (40.9)	52 (59.1)	AKI stage ≥ R	At ICU admission
12	Cruz et al. 2010 [35]	Critically ill patients	Serum NGAL	AKI during ICU stay	RIFLE	No	301	168 (55.8)	133 (44.2)	AKI stage R, I, F	Daily from ICU admission to 4 days after ICU admission
13	de Geus et al. 2011 [36]	Critically ill patients	Urine NGAL Serum NGAL	AKI with 7 days of ICU stay	RIFLE	No	632	461 (72.9)	171 (27.1)	AKI stage R, I, F	At ICU admission
14	Endre et al. 2011 [37]	Critically ill patients	Urinary NGAL/Cr Urinary CysC/Cr Urinary IL-18/Cr Urinary KIM-1/Cr	AKI, Mortality within 7 days	AKIN and RIFLE	No	528	381 (72.2)	147 (27.8)	AKI stage ≥ R or ≥ 1	At ICU admission, and at 12 and 24 h after admission
15	Breidthardt et al. 2012 [38]	Acute heart failure patients presented to emergency department	Serum NGAL	AKI	AKIN	No	207	147 (71)	60 (29)	AKI stage 1, 2, 3	Every 6 h from ER presentation to 48 h after ER
16	Camou et al. 2013 [39]	Critically ill adult with septic shock	Serum NGAL	AKI at ICU admission, and 24 h, 48 h	RIFLE/AKIN	No	50	7 (14)	43 (86)	AKI stage R, I, F, AKI stage 1, 2, 3	at ICU admission, and 24 h, 48 h
17	Doi et al. 2013 [40]	Cardiac surgical patients	Serum NGAL	AKI	AKIN	No	146	93 (63.7)	53 (36.3)	AKI stage ≥ 1	Pre-op, post-op 0, 2, 4, 12, 24, 36, 60 h
18	Gaipov et al. 2015 [41]	Cardiac surgical patients	Urinary NGAL Serum NGAL	Post-op AKI within 12 h, 24 h, 48 h and RRT	KDIGO	No	60	40 (66.7)	20 (33.3)	AKI stage 1, 2, 3, RRT	Post-op 2 h
19	Cuartero et al. 2019 [42]	Critically ill patients	Serum NGAL	AKI and ICU admission and 48 h later	AKIN and KDIGO	No	100	57 (57)	43 (43)	AKI stage 1, 2, 3	At ICU admission, and 24, 48 h later
20	Khawaja et al. 2019 [43]	Critically ill patients with suspected sepsis	Serum NGAL	Sepsis-related AKI	RIFLE	No	46	22 (47.8)	24 (52.2)	AKI stage ≥ R	12, 24, and 48 h after ICU admission
21	Mosa et al. 2018 [44]	Cardiothoracic surgery using cardiopulmonary bypass	Serum NGAL	Post-op AKI	KDIGO	No	182	117 (64.3)	65 (35.7)	AKI stage ≥ 1	Before CPB and at 0, **2**, 12, 24 h after CPB
22	Sun et al. 2017 [45]	Scrub typhus-associated AKI	Serum NGAL Serum KIM-1 Urinary NGAL/Cr Urinary KIM-1/Cr	Scrub typhus–associated AKI	RIFLE	Yes	138	113 (81.9)	25 (18.1)	AKI stage R, I, F	Admission (n = 138) and 3 days after taking the initial sample (n = 37)
23	Ghonemy et al. 2014 [46]	Cardiac surgery (CPB & valve replacement surgery)	Serum NGAL Serum CysC	Post-op AKI	N.A	No	50	33 (66)	17 (34)	Creatinine level at 24 h being elevated either by 25% of the basal level or by 0.3 mg/dL above the basal level	Baseline, and post-op 3, 6, 24 h
24	Padhy et al. 2014 [47]	Patients received percutaneous coronary intervention	Serum NGAL Serum CysC	Contrast-induced AKI	N.A	No	60	30 (50)	30 (50)	by a rise in serum creatinine level of at least 0.5 mg/dL from the baseline value at 48 h	0, 4, 24, 48 h after coronary angiography
25	Geus et al. 2013 [48] (no sepsis)	ICU patients	Serum NGAL	AKI within 24 h after ICU admission	AKIN	No	542	427 (78.8)	115 (21.2)	AKI stage ≥ 1	ICU Admission (0 h) and at 4, 8, 24 h after ICU Admission
25	Geus et al. 2013 [48] (sepsis)	ICU patients	Serum NGAL	AKI within 24 h after ICU admission	AKIN	No	75	25 (33.3)	50 (66.7)	AKI stage ≥ 1	ICU Admission (0 h) and at 4, 8, 24 h after ICU Admission
26	Haase-Fielitz et al. 2009 [49]	Cardiac surgery	Serum NGAL, Serum CysC	Post-op AKI and 24 h after OP	SCr increase > 50% from baseline; RIFLE	No	100	77 (77)	23 (23)	AKI stage R, I, F	Baseline, post-op 6 h and 24 h
27	Hanson et al. 2011 [50]	Severe malaria	Urinary NGAL Serum Cr	RRT	N.A	No	163	79 (48.5)	84 (51.5)	RRT	On study enrollment
28	Introcaso et al. 2018 [51]	Cardiac surgery	Serum NGAL	Post-op AKI	KDIGO	Yes	69	45 (65.2)	24 (34.8)	AKI stage 1, 2, 3	Pre-op and within post-op 4 h in ICU
29	Kim et al. 2017 [52]	Critically ill patients with suspected sepsis	Serum NGAL Serum PENK	AKI, mortality	KDIGO	No	167	126 (75.4)	41 (24.6)	AKI stage ≥ 1, RRT	On study enrollment
30	Ferrari et al. 2019 [53]	Critically ill adult	Urinary TIMP-2 × IGFBP-7	AKI within **12 h**, 24 h, 48 h and 7 days	KDIGO	Yes	442	254 (57.5)	188 (42.5)	AKI stage ≥ 1; RRT	ICU admission
31	Xie et al. 2019 [54]	ICU patients	Urinary TIMP-2 × IGFBP-7	CRRT, mortality, length of ICU stay	KDIGO Stage AKI 1, 2, 3	Yes	719	480 (66.8)	239 (33.2)	AKI stage ≥ 1	immediately upon enrollment
32	Adler et al. 2018 [55]	Out-of-hospital cardiac arrest	Urinary TIMP-2 × IGFBP-7	AKI	KDIGO Stage AKI 1, 2, 3	Yes	48	17 (35.4)	31 (64.6)	AKI stage ≥ 1	3 h and 24 h after OHCA
33	Oezkur et al. 2017[ 56]	Cardiac surgery	Urinary TIMP-2 × IGFBP-7	AKI within 48 h after op	KDIGO	Yes	100	80 (80)	20 (20)	Unknown	Before surgery (baseline), ICU admission (directly after Surgery), 24 h post-surgery
34	Wang et al. 2017 [57]	Cardiac surgery	Urinary TIMP-2 × IGFBP-7	AKI within 7 days after op	KDIGO	Yes	57	37 (64.9)	20 (35.1)	AKI stage 2 or 3	Before surgery, ICU admission (in 2-h intervals from 0 to 12 h after Surgery), 24 h after ICU admission
35	Finge et al. 2017 [58]	Cardiac surgery with cardiopulmonary bypass	Urinary TIMP-2 × IGFBP-7	AKI within 48 h after op	KDIGO	Yes	93	59 (63.4)	34 (36.6)	AKI stage ≥ 1	Before surgery and 3-h postoperative period
36	Cuartero et al. 2017 [59]	Septic and non-septic critically ill patients	Urinary TIMP-2 × IGFBP-7	AKI	AKIN	Yes	98	49 (50)	49 (50)	AKI stage ≥ 2, RRT	at ICU admission and up to 12 h later simultaneously with the morning blood work
37	Mayer et al. 2017 [60]	Cardiac surgery with cardiopulmonary bypass	Urinary TIMP-2 × IGFBP-7	Post-op AKI	KDIGO and RIFLE	Yes	110	101 (91.8)	9 (8.2)	AKI stage 1, 2, 3; stage R, I, F	Pre-op and at 1, 4, 24 h after surgery
38	Meersch et al. 2014 [61]	Cardiac surgery with cardiopulmonary bypass	Urinary TIMP-2 × IGFBP-7	Post-op AKI	AKIN or KDIGO	Yes	50	24 (48)	26 (52)	AKI stage 1, 2, 3	Pre-op and 4, 12, 24 h after CPB
39	Dusse et al. 2016 [62]	Cardiac surgery	Urinary TIMP-2 × IGFBP-7	AKI stage 2 or 3 within 48 h after op	KDIGO	Yes	40	32 (80)	8 (20)	AKI stage 2, 3	post-op 4 h and then twice daily until discharge from ICU (maximum 4 days)
40	Gunnerson et al. 2016 [63]	Critically ill patients	Urinary TIMP-2 × IGFBP-7	AKI stage 2 or 3	KDIGO	No	375	340 (90.7)	35 (9.3)	AKI stage 2, 3	Within 12 h of ICU admission
41	Wetz et al. 2015 [64]	Cardiac surgery	Urinary TIMP-2 × IGFBP-7	Post-op AKI Stage 1 or 2	KDIGO	No	42	26 (61.9)	16 (38.1)	AKI stage 1, 2	Baseline; End of surgery; 4 h after arrest of CPB; 1 day after surgery
42	Kimmel et al. 2016 [65]	ER patient	Urinary TIMP-2 × IGFBP-7	Positive U scores at enrollment		No	362	347 (95.9)	15 (4.41)	Unknown	Admission
43	Pilarczyk et al. 2015 [66]	Post-cardiac surgery	Urinary TIMP-2 × IGFBP-7	Post-op AKI stage 2 or 3 within 48 h	KDIGO	No	60	41 (68.3)	19 (31.7)	AKI stage 1,2,3	Post-op 4 h and every 12 h until discharge
44	Hoste et al. 2014 [67]	Critically ill patients	Urinary TIMP-2 × IGFBP-7	AKI stage 2 or 3 within 12 h	KDIGO	Partial	153	27 (17.6)	126 (82.4)	AKI stage 1,2,3	ICU admission
45	Cummings et al. 2018 [68]	Cardiac surgery	Urinary TIMP-2 × IGFBP-7	Post-op AKI stage 2 or 3 within 48 h	KDIGO	No	400	309 (77.3)	91 (22.7)	AKI stage 1, 2, 3	Immediately after CPB
46	Katagiri et al. 2012 [69]	Cardiac surgery	Urinary L-FABP	Post-op AKI	AKIN	No	77	49 (63.6)	28 (36.4)	Unknown	Pre-op, 0,4,12 h after ICU admission
47	Doi et al. 2011 [70]	Critically ill patients admitted to medical–surgical mixed ICU	Urinary L-FABP Urinary NGAL Urinary IL-18	AKI during admission	RIFLE	No	339	208 (61.4)	131 (38.6)	Unknown	12 h after ICU admission
48	Ferguson et al. 2010 [71]	Ordinary ward and ICU	Urinary L-FABP Urinary NGAL Urinary KIM-1Urinary IL-18 Urinary NAG	AKI	≥ 50% increase in SCr from baseline	No	160	68 (42.5)	92 (57.5)	unknown	NA
49	Li et al. 2012 [72]	Liver transplantation	Urinary L-FABP Urinary NGAL	AKI	AKIN	No	25	14 (56)	11 (44)	Unknown	0,2,4,6,12,24,48, 72,120 h after the anhepatic phase
50	Manabe et al. 2012 [73]	Cardiac catheterization	Urinary L-FABP	Contrast-induced AKI within 48 h	AKIN	No	220	201 (91.4)	19 (8.6)	Unknown	on day 0, 1 and 2 after contrast medium exposure
51	Matsui et al. 2012 [74]	Cardiac surgery	Urinary NGAL Urinary L-FABP	Post-op AKI within 48 h	AKIN	No	85	37 (43.5)	48 (56.5)	Unknown	Before OP, 0,3,6,18,24 and 48 h after OP
52	Khreba et al. 2019 [75]	Post-cardiopulmonary bypass in open heart surgery	Urinary KIM-1	Post-op AKI	KDIGO	No	45	18 (40)	27 (60)	Unknown	Post-op 3 h
53	Tu et al. 2014 [76]	Sepsis	Urinary KIM-1	Sepsis-related AKI	AKIN	No	150	101 (67.3)	49 (32.7)	Unknown	0,1,3,6,24,48 h after ICU admission
54	Parikh et al. 2005 [77]	ARDS	Urinary IL-18	AKI within the first 6 days of ARDS	Increase in SCr by at least 50%	No	138	86 (62.3)	52 (37.7)	Unknown	ICU admission 0,1,3 day
55	Parikh et al. 2004 [78]	Kidney transplant patients	Urinary IL-18	ATN	SCr from normal to > 3 mg/dL (> 265 umol/L)	No	72	50 (69.4)	22 (30.6)	Unknown	24 h after op
56	Han et al. 2009 [79]	Cardiac surgery	Urinary KIM-1/Cr	Post-op AKI within 72 h after surgery	AKIN	No	90	54 (60)	36 (40)	Unknown	0,3,18,24 h after op
57	Liangos et al. 2009 [80]	Cardiac surgery (Cardiopulmonary bypass)	Urinary KIM-1 Urinary NAG Urinary NGAL Urinary IL-18 Urinary CysC Urinary α1-microglobulin	Post-op AKI within 72 h	Cre inc > 50% in 72 h	No	103	90 (87.4)	13 (12.6)	Unknown	2 h
58	Naggar et al. 2012 [81]	Critically ill patients	Urinary KIM-1	AKI	RIFLE	No	40	20 (50)	20 (50)	Unknown	0,24,48 h
59	Nickolas et al. 2012 [82]	ER patients	Urinary KIM-1 Urinary NGAL Urinary IL-18 Urinary L-FABP Urinary CysC	AKI	RIFLE	No	1635	1539 (94.1)	96 (5.9)	Unknown	0 h ER
60	Vaidya et al. 2008 [83]	Inpatient nephrology consultation service	Urinary KIM-1 Urinary NGAL Urinary IL-18 Urinary HGF Urinary CysC Urinary NAG Urinary VEGF Urinary CXCL 10 Urinary Total protein	AKI	RIFLE	No	204	102 (50)	102 (50)	Unknown	0 h
61	Nisula et al. 2015 [84]	ICU patients	Urinary IL-18	AKI	KDIGO on Day 2 or Day 3	YES	1439	942 (65.5)	497 (34.5)	AKI Stage3 RRT	0-24 h
62	Nickolas TL et al. 2008 [85]	ER patients	Urinary NGAL Urinary NAG Urinary α1-microglobulin Urinary α1-acid glycoprotein	AKI	RIFLE-R	No	635	605 (95.3)	30 (4.7)	RRT	ED presentation
63	Cho et al. 2013 [86]	Critically ill patients admitted to medical–surgical mixed ICU	Urinary NGAL Urinary L-FABP	AKI	AKIN	No	145	91 (62.8)	54 (37.2)	AKIN stage 1,2,3 RRT	ICU admission
64	Park et al. 2019 [87]	Sepsis	Urinary NGAL	Sepsis-related AKI	KDIGO	No	140	121 (86.4)	19 (13.6)	Unknown	0 h
65	Perry et al. 2010[88]	Cardiac Surgical	Serum NGAL	Post-op AKI within 4 days	50% increase in serum	No	879	804 (91.5)	75 (8.5)	Unknown	0 h
66	Shapiro et al. 2010 [89]	Sepsis	Serum NGAL/Cr	AKI	AKI/ > 0.5 mg/dL in 72 h	No	661	637 (96.4)	24 (3.6)	RIFLE-I RIFLE-R	12,24,48,72 h
67	Thanakitcharu et al. 2014 [90]	Open cardiac surgery	Urinary NGAL	Post-op AKI	AKIN	No	130	84 (64.6)	46 (35.3)	Unknown	0,3,6 h after surgery
68	Valette et al. 2013 [91]	Contrast-induced	Serum NGAL	Contrast-related AKI within 72 h	AKIN	No	98	68 (64.6)	30 (35.4)	RRT	0,2,6,24 h
69	Varela et al. 2015 [92]	Cardiac surgery	Urinary NGAL	Post-op AKI	AKIN	No	66	50 (75.8)	16 (24.2)	Unknown	0,1,6,24 h after surgery
70	Chen et al. 2012 [93]	CCU, AMI	Serum NGAL, Urinary NGAL/Cr Urinary IL-18/Cr Urinary Cystatin C	AKI	AKIN	No	150	107 (71.3)	43 (28.7)	Unknown	after CCU admission
71	Nisula et al. 2014 [94]	Critically ill	Urinary NGAL	AKI < 72 h	KDIGO	No	1042	663 (63.6)	379 (36.4)	AKI Stage 1.2.3 RRT	ICU arrival, 12 h 24 h after admission
72	Maisel et al. 2016 [95]	Acute heart failure	Serum NGAL	Worsening renal function < 5 days	increase in plasma creatinine of 0.5 mg/dL or ≥ 50% above first value or initiation of acute renal replacement therapy	No	927	855 (92.2)	72 (7.8)	Unknown	Acute heart failure requiring intravenous diuretic agents. 2,6 h, 1,2,3d
73	Matsa et al. 2014 [96]	Critically ill	Serum NGAL Urinary NGAL	AKI < 72 h	RIFLE	No	194	135 (69.6)	59 (30.4)	Unknown	0,24,48,72 h ICU arrival
74	Munir et al. 2013 [97]	Cardiopulmonary bypass	Urine NGAL	Post-op AKI < 48 h	AKIN	No	88	77 (87.5)	11 (12.5)	Unknown	4 h after CPB
75	Onk et al. 2016 [98]	Cardiac surgery	Serum IL-6 Serum NGAL Serum SCr	Post-op AKI < 7 days	RIFLE	No	90	45 (50)	45 (50)	RIFLE-R,I,F	Pre-op 1,6,12,24,36 h,7d
76	AZRINA MD RALIB et al. 2017 [99]	Critically ill	Serum NGAL	AKI	KDIGO	No	225	138 (61.3)	87 (38.7)	Unknown	within 24 h of ICU admission
77	Yang et al. 2016 [100]	Heart failure	Urinary NGAL Urinary KIM-1 Urinary NGAL/Cr Urinary KIM-1/Cr Serum CysC	AKI	KDIGO	No	103	54	49	Unknown	Admission to ICU
78	Ueta et al. 2014 [101]	Endovascular stent graft repair of aortic aneurysm	Urinary NGAL/Cr Urinary NGAL Serum NGAL Serum L-FABP Serum L-FABP/Cr	Post-op AKI	AKIN	No	42	36	6	Unknown	2 h post-op 0 h, 2 h, 6 h, 1d, 3d, 4d
79	Chang et al. 2015 [102]	CCU patients	Urinary NGAL NGAL/Cr	Pre-renal and intrinsic AKI	KDIGO	No	147	76	71	Unknown	Admission to CCU
80	Hjortrup et al. 2014 [103]	ICU severe sepsis	Serum NGAL Urinary NGAL	AKI	KDIGO	No	222	191	31	AKI stage ≥ 1, RRT	On study enrollment
81	Chen et al. 2020 [104]	CCU patients	Serum IL-18 Serum NGAL Serum CysC Urinary NGAL Urinary NGAL/Cr	AKI	KDIGO	No	269	217	52	Unknown	Admission to CCU
82	Wybraniec et al. 2017 [105]	Contrast-induced acute kidney injury	Urinary KIM-1, Urinary IL-18	Contrast-induced AKI	KDIGO	No	95	86	9	Unknown	6 h after procedure
83	Sinkala et al. et al. 2016 [106]	Hospitalized patients	Urinary KIM-1	AKI	unknown	unknown	40	27	13	Unknown	Cross-sectional
84	Torregrosa et al. et al. 2014 [107]	Acute coronary syndrome or heart failure or undergoing coronary angiography	Urinary L-FABP Urinary KIM-1 Urinary NGAL	AKI	RIFLE	No	144	124	20	Unknown	12 h after procedure
85	Tekce et al. 2014 [108]	Patient received cisplatin	Urinary KIM-1, Serum KIM-1	AKI	Cre > 1.5–twofold	No	22	14	8	Unknown	Day 0, 1,3,5
86	Torregrosa et al. 2012 [109] (M)	Acute coronary syndrome	Urinary IL-18 Urinary NGAL	AKI	KDIGO		89	77	12	Unknown	12 h after procedure
86	Torregrosa et al. 2012 [109] (S)	Cardiac surgery	Urinary IL-18 Urinary NGAL	Post-op AKI	RIFLE, Cre inc > 50%	No	46	32	14	Unknown	12 h after surgery
87	Matsui et al. 2011 [110]	ICU patients	Urinary L-FABP/Cr Urinary NAG/Cr	AKI	AKIN (incre > 0.3, 50%)	No	25	11	14	Unknown	0 h after ICU
88	Parikh et al. 2011 [111]	Cardiac surgery	Serum NGAL Urinary NGAL Urinary IL-18	Post-op AKI	RIFLE	R	1219	1159	60	Unknown	0–5 day after surgery
89	Wang 2017 [112]	Cardiopulmonary bypass	Urinary IL-18	Post-op AKI	Cre increase > 50%	No	103	81	22	Unknown	Before CPB, at 2 h, 4 h, 6 h, 8 h and 12 h after CPB
90	Haase-Fielitz et al. 2009 [113]	Cardiac surgery	Serum NGAL	Post-op AKI	Cre increase > 50% within 168 h	No	100	77	23	RIFLE-I,F AKIN-2,3 RRT	6 h after start CPB
91	Waskowski 2021 [114]	Cardiac surgery	11. TIMP-2 × IGFBP-7: 0.3 12. TIMP-2 × IGFBP-7: 2	Post-op AKI	KDIGO	Yes	93	62 (67)	31 (33)	AKI stage ≥ 1	Post-op day 1
92	Imoto 2021 [115]	ICU patients	07. NGAL	AKI	KDIGO	Yes	106	35 (33)	71 (67)	AKI Stage 3	Day 1
93	Lee 2021 [116]	Cardiac surgery	05. L-FABP 06. L-FABP/Cr	Post-op AKI	KDIGO	Yes	144	85 (59)	59 (41)	AKI stage ≥ 1	Post-op 16–18 h
94	Szymanowicz 2021 [117]	Cardiac surgery	07. NGAL	Post-op AKI	KDIGO	No	114	96 (84)	18 (16)	AKI stage ≥ 1	3 h after OP
95	Zhen 2021 [118]	Acute coronary syndrome	09. Serum NGAL	AKI	AKIN	No	172	149 (87)	23 (13)	AKI stage ≥ 1	6 h after admission
96	Obata 2021 [119]	Open abdominal aortic aneurysm repair	06. L-FABP/Cr 08. NGAL/Cr	Post-op AKI	KDIGO	No	64	45 (70)	19 (30)	AKI stage ≥ 1	Pre-op, post-induction, 2 h post-AXC, Post-op, 4 h and 2 day
97	Qiu 2021 [120]	Sepsis	07. NGAL	Sepsis-related AKI	KDIGO	Yes	90	46 (51)	44 (49)	AKI stage ≥ 1	at ICU admission
98	Shakked 2022 [121]	COVID-19 patients	09. Serum NGAL	AKI	KDIGO	No	52	30 (58)	22 (42)	AKI stage ≥ 1, RRT	ER presentation
99	Vogel 2021 [122]	COVID-19 patients	04. KIM-1/Cr	AKI	KDIGO	No	54	46 (85)	8 (15)	AKI stage ≥ 1	ER presentation
100	Ergun 2021 [123]	Major surgery	09. Serum NGAL	Post-op AKI	AKIN	Yes	60	47 (78)	13 (22)	AKI stage ≥ 1	Pre-op, Post-op 6 h,24 h
101	Pilarczyk 2022 [124]	Thoracic aortic surgery	10. TIMP-2 × IGFBP-7: custom	Post-op AKI	KDIGO	Yes	101	74 (73)	27 (27)	AKI stage 2 or 3	Pre-op, Post-op 2 h, 6 h, POD 1
102	Okuda 2022 [125]	Emergency laparotomy	06. L-FABP/Cr	Post-op AKI	KDIGO	Yes	48	38 (79)	10 (21)	AKI stage ≥ 1	Pre-op, Post-op 2 h, 4 h, 6 h, 24 h, 48 h, 72 h
103	Pei 2022 [126]	Sepsis	09. Serum NGAL	Sepsis-related AKI	KDIGO	Yes	162	102 (63)	60 (37)	AKI stage ≥ 1	ER presentation
104	Jahaj 2021 [127]	ICU patients	09. Serum NGAL	AKI	RIFLE	Yes	266	168 (63)	98 (37)	AKI stage ≥ 1	24 h after ICU admission
105	Garms 2021 [128]	Patients received vancomycin	07. NGAL	Drug-related AKI	KDIGO	Yes	94	71 (76)	23 (24)	AKI stage ≥ 1	The first day of vancomycin use
106	Irqsusi 2021 [129]	Cardiac surgery	10. TIMP-2 × IGFBP-7: custom 11. TIMP-2 × IGFBP-7: 0.3 12. TIMP-2 × IGFBP-7: 2	Post-op AKI	KDIGO	Yes	50	36 (72)	14 ( (28)	AKI stage ≥ 1	Post-op 0.5 h, 1 h and 0, 6, 12, and 24 h after ICU admission
107	Guray 2021 [130]	Patients undergoing coronary angiography	09. Serum NGAL	Contrast-induced nephropathy	an increase of over 25% or equal to or over 44.2 μmol/L in baseline SCr at 48–72 h after cardiac catheterization	No	84	68 (81)	16 (19)	AKI stage ≥ 1	Before and at 4 and 24 h after the procedure
108	Tan 2022 [131]	Ureteroscopic lithotripsy-related urosepsis	01 IL-18 03. KIM-1 07. NGAL	Sepsis-related AKI	KDIGO	Yes	157	121 (77)	36 (23)	AKI stage ≥ 1	0, 4, 12, 24 and 48 h after the surgery
109	Lakhal 2021 [132]	Cardiac surgery patients	02. 11. TIMP-2 × IGFBP-7: 0.3	Post-op AKI	KDIGO	Yes	65	38 (58)	27 (42)	AKI stage ≥ 1	before CPB and post-CPB 6 h, 24 h
110	Sahu 2022 [133]	Patients undergoing percutaneous coronary intervention	03. 09. Serum NGAL	Contrast-induced nephropathy	an increase in SCr by > 0.5 mg/dL or > 25%, assessed at 48 h after the procedure	No	212	187 (88)	25 (12)	AKI stage ≥ 1	4 and 48 h after the procedure

*AKI* acute kidney injury, *AKIN* Acute Kidney Injury Network, *ARDS* acute respiratory distress syndrome, *ATN* acute tubular necrosis, *CCU* cardiac care unit, *Cr* creatinine, *CPB* cardiothoracic surgery using cardiopulmonary bypass, *CysC* cystatin C, *ER* emergency room, *ICU* intensive care unit, *IL-18* interleukin-18, *KDIGO* Kidney Disease Improving Global Outcomes, *KIM-1* kidney injury molecule-1, *L-FABP* liver-type fatty acid-binding protein, *NGAL* neutrophil gelatinase-associated lipocalin, *PENK* proenkephalin, *RIFLE* Risk, Injury, Failure, Loss, and End-stage renal disease, *SCr* serum creatinine, *TIMP-2 × IGFBP-7* tissue inhibitor of metalloproteinases-2 × insulin-like growth factor-binding protein-7, *UOC* urine output criteria

**Table 2:** Summary of included comparative studies for outcome evaluation

No	Study (year)	Mean age	Male gender %	Diabetes%	Chronic kidney disease%	Heart failure%	Sepsis%	Surgery%	SOFA score
1	Qian et al. 2019 [24]	61.8	58 (63.7)	14 (15.4)	0%	13 (14.3)	Unknown	100%	Unknown
2	Prowle et al. 2015 [25]	70	64 (69)	7 (7)	37%	6 (6)	Unknown	100%	Unknown
3	Lei et al. 2018 [26]	60.6	91 (60.7)	0%	0%	0%	0%	0%	Unknown
4	van Wolfswinkel et al. 2016 [27]	45.5	33 (84.6)	Unknown	Unknown	Unknown	Unknown	0%	Unknown
5	Srisawat et al. 2015 [28]	39.8	94 (83.2)	Unknown	Unknown	Unknown	Unknown	Unknown	Unknown
6	Zeng et al. 2014 [29]	55.3	109 (55.3)	46 (23.4)	0%	Unknown	Unknown	100%	Unknown
7	Aydoğdu et al. 2013 [30]	67.7	98 (64.9)	44 (29.1)	0%	55 (36.4)	129 (85.4)	Unknown	6
8	Liu et al. 2013 [31]	63	72 (66.1)	28 (25.7)	10 (9.2)	22 (20.2)	19 (17.4)	100%	Unknown
9	Wagener et al. 2011 [32]	54.3	60 (65.2)	Unknown	Unknown	Unknown	Unknown	100%	Unknown
10	Makris et al. 2009 [33]	46	25 (80.6)	Unknown	Unknown	Unknown	Unknown	Unknown	7
11	Constantin et al. 2010 [34]	57	Unknown	Unknown	0%	Unknown	45 (51)	36 (40.9)	7
12	Cruz et al. 2010 [35]	64	207 (68.8)	47 (15.6)	20 (6.6)	Unknown	115 (38.2)	137 (45.5)	5
13	de Geus et al. 2011 [36]	60.1	369 (58.4)	Unknown	0 (0)	Unknown	43 (6.8)	192 (30.4)	8
14	Endre et al. 2011 [37]	60	318 (60.2)	Unknown	Unknown	Unknown	101 (19.1)	310 (58.7)	6.3
15	Breidthardt et al. 2012 [38]	80	122 (58.9)	69 (33)	92 (44)	103 (50)	Unknown	Unknown	Unknown
16	Camou et al. 2013 [39]	60.3	38 (76)	Unknown	Unknown	Unknown	100%	Unknown	12
17	Doi et al. 2013 [40]	69	92 (63)	59 (40.4)	68 (46.6)	Unknown	Unknown	100%	Unknown
18	Gaipov et al. 2015 [41]	56.7	42 (70)	18 (45)	Unknown	6 (15)	3 (7.5)	100%	Unknown
19	Cuartero et al. 2019 [42]	59.1	60 (60)	Unknown	Unknown	Unknown	29 (29)	39%	6.5
20	Khawaja et al. 2019 [43]	46.5	32 (69)	2 (4.3)	Unknown	Unknown	100%	Unknown	Unknown
21	Mosa et al. 2018 [44]	64	97 (53.3)	57 (31.3)	Unknown	Unknown	Unknown	100%	Unknown
22	Sun et al. 2017 [45]	65	49 (36)	26 (19)	9 (7)	Unknown	Unknown	Unknown	Unknown
23	Ghonemy et al. 2014 [46]	43	32 (64)	0%	0%	Unknown	Unknown	100%	Unknown
24	Padhy et al. 2014 [47]	55.9	44 (73.3)	7 (11.7)	Unknown	Unknown	Unknown	100%	Unknown
25	Geus et al. 2013 [48] (no sepsis)	57.9	347 (59.9)	Unknown	0%	Unknown	0%	0%	Unknown
25	Geus et al. 2013 [48] (sepsis)	57.6	38 (47.5)	Unknown	0%	Unknown	100%	0%	Unknown
26	Haase-Fielitz et al. 2009 [49]	71.8	61 (61)	28 (28)	0%	Unknown	Unknown	100%	Unknown
27	Hanson et al. 2011 [50]	35	130 (80)	Unknown	Unknown	Unknown	Unknown	Unknown	Unknown
28	Introcaso et al. 2018 [51]	77	44 (63.8)	Unknown	Unknown	Unknown	Unknown	100%	Unknown
29	Kim et al. 2017 [52]	70	99 (59.3)	Unknown	Unknown	Unknown	100%	Unknown	Unknown
30	Ferrari et al. 2019 [53]	68	276 (62.4)	76 (17.2)	0%	Unknown	80 (18.1)	64 (14.5)	6
31	Xie et al. 2019 [54]	68.2	439 (61.1)	114 (15.9)	98 (13.6)	Unknown	87 (12.1)	103 (14.3)	7
32	Adler et al. 2018 [55]	63	44 (91.7)	8 (17)	11 (23)	42 (88)	6 (12.5)	Unknown	Unknown
33	Oezkur et al. 2017 [56]	68.5	70 (70)	Unknown	0%	46 (46)	Unknown	100%	Unknown
34	Wang et al. 2017 [57]	60	41 (71.9)	8 (14)	2 (3.5)	100% (I-IV)	Unknown	100%	Unknown
35	Finge et al. 2017 [58]	70.5	53 (57)	21 (22.6)	0%	Unknown	Unknown	100%	Unknown
36	Cuartero et al. 2017 [59]	55	65 (66.3)	15 (15.3) table S1	6 (6.1) table S1	Unknown	40 (40.8)	Unknown	7.5
37	Mayer et al. 2017 [60]	68	87 (79.1)	9 (8.2)	9 (8.2)	6 (5.5)	Unknown	100%	Unknown
38	Meersch et al. 2014 [61]	71	33 (66)	20 (40)	15 (30)	46 (92)	Unknown	100%	Unknown
39	Dusse et al. 2016 [62]	81.2	16 (40)	13 (32.5)	Unknown	Unknown	2 (5)	100%	Unknown
40	Gunnerson et al. 2016 [63]	64.3	242 (64.5)	101 (26.9)	40 (10.7)	61 (16.3)	44 (11.7)	261 (69.6)	Unknown
41	Wetz et al. 2015 [64]	72	29 (69)	IDDM 10 (23.8)	26 (61.9)	18 (42.9)	Unknown	41 (97.6)	Unknown
42	Kimmel et al. 2016 [65]	67	241 (67)	82 (23)	39 (11)	81 (22)	Unknown	Unknown	Unknown
43	Pilarczyk et al. 2015 [66]	69.6	48 (80)	21 (35)	Unknown	4 (6.7)	8 (13.3)	100%	Unknown
44	Hoste et al. 2014 [67]	64.5	87 (56.9)	unknown	13 (8.5)	Unknown	29 (19)	23 (15)	Unknown
45	Cummings et al. 2018 [68]	67	269 (67.3)	123 (30.8)	132 (33)	163 (40.8)	Unknown	100%	Unknown
46	Katagiri et al. 2012 [69]	64.25	47 (61)	23 (29.9)	6 (7.8)	Unknown	Unknown	100%	Unknown
47	Doi et al. 2011 [70]	66	223 (65.8)	94 (27.7)	Unknown	Unknown	66 (19.5)	175 (51.6)	Unknown
48	Ferguson et al. 2010 [71]	58	111 (69.4)	Unknown	Unknown	Unknown	AKI group 33 (35.9)	54 (33.8)	Unknown
49	Li et al. 2012 [72]	47	22 (88)	Unknown	Unknown	Unknown	Unknown	100%	Unknown
50	Manabe et al. 2012 [73]	71.7	29 (13.2)	69 (31.4)	220 (100)	Unknown	Unknown	0%	Unknown
51	Matsui et al. 2012 [74]	71.7	64 (75)	27 (36)	Unknown	Unknown	Unknown	100%	Unknown
52	Khreba et al. 2019 [75]	46.3	23 (51.1)	15 (33.3)	Unknown	Unknown	Unknown	100%	Unknown
53	Tu et al. 2014 [76]	57.3	93 (62)	17 (11.3)	Unknown	Unknown	100%	100%	Unknown
54	Parikh et al. 2005 [77]	50	72 (52.2)	Unknown	Unknown	Unknown	29 (21)	Unknown	Unknown
55	Parikh et al. 2004 [78]	44	44 (61.1)	Renal Transplant group 8 (36.4)	22 (30.6)	Unknown	ATN group 6 (42.9)	26 (36.1)	Unknown
56	Han et al. 2009 [79]	63.56	61 (67.8)	Unknown	Unknown	Unknown	Unknown	100%	Unknown
57	Liangos et al. 2009 [80]	68	74 (72)	29 (28.2)	Unknown	23 (22.3)	Unknown	100%	Unknown
58	Naggar et al. 2012 [81]	51	16 (40)	Unknown	Unknown	Unknown	Unknown	Unknown	13
59	Nickolas et al. 2012 [82]	64.4	(52.3)	29.4%	25.2	8.2%	3.4%	Unknown	Unknown
60	Vaidya et al. 2008 [83]	61.2	55%	Unknown	Unknown	Unknown	34%	Unknown	Unknown
61	Nisula et al. 2015 [84]	63	920 (63.9)	326 (22.7)	86 (6)	165 (11.5)	89 (6.2)	485 (33.7)	7
62	Nickolas TL et al. 2008 [85]	60.1	331 (51)	Unknown	106 (16.7)	Unknown	Unknown	Unknown	Unknown
63	Cho et al. 2013 [86]	62.9	85 (58.6)	41 (28.3)	20 (13.8)	Unknown	Unknown	70 (48.3)	Unknown
64	Park et al. 2019 [87]	75	67 (47.9)	Unknown	Unknown	Unknown	85 (60.7)	Unknown	Unknown
65	Perry et al. 2010 [88]	65	704 (80)	298 (33.9)	Unknown	Unknown	Unknown	100%	Unknown
66	Shapiro et al. 2010 [89]	59	318 (48)	188 (28)	Unknown	Unknown	100%	Unknown	Unknown
67	Thanakitcharu et al. 2014 [90]	51.1	76 (58.5)	21 (16.2)	Unknown	54 (35.8	Unknown	100%	Unknown
68	Valette et al. 2013 [91]	60	74 (75)	15 (15)	4 (4)	8 (8)	Unknown	Unknown	8
69	Varela et al. 2015 [92]	68	49 (74)	15 (23)	Unknown	Unknown	Unknown	100%	Unknown
70	Chen et al. 2012 [93]	66	113 (75)	92 (61)	Unknown	Unknown	30 (20)	Unknown	Unknown
71	Nisula et al. 2014 [94]	63	673 (64.6)	242 (23.2)	74 (7.1)	139 (13.5)	67 (6.4)	362 (34.7)	8
72	Maisel et al. 2016 [95]	68.5	(62)	(43.6)	(25.9)	Unknown	Unknown	Unknown	Unknown
73	Matsa et al. 2014 [96]	60.1	104 (56)	Unknown	Unknown	Unknown	15 (8)	76 (39)	Unknown
74	Munir et al. 2013 [97]	52	76 (86)	Unknown	Unknown	Unknown	Unknown	100%	Unknown
75	Onk et al. 2016 [98]	66	52 (58)	26 (29)	Unknown	Unknown	Unknown	100%	Unknown
76	Azrina Md Ralib et al. 2017 [99]	47	151 (67)	Unknown	Unknown	Unknown	129 (57)	98 (43.6)	8
77	Yang et al. 2016 [100]	68	71 (68.9)	Unknown	Unknown	Unknown	Unknown	Unknown	Unknown
78	Ueta et al. 2014 [101]	69.7	60%	25	Unknown	Unknown	Unknown	100%	Unknown
79	Chang et al. 2015 [102]	67	100 (68)	63 (43)	47 (32)	60 (41)	17 (12)	unknown	Unknown
80	Hjortrup et al. 2014 [103]	66	126 (57)	16 (7)	47 (21)	Unknown	100%	98 (44)	8
81	Chen et al. 2020 [104]	64	202 (75)	110 (41)	unknown	Unknown	15 (5.6)	Unknown	Unknown
82	Wybraniec et al. 2017 [105]	65	69.50%	39%	Unknown	Unknown	Unknown	Unknown	Unknown
83	Sinkala et al. et al. 2016 [106]	35.6	50 (62.5)	Unknown	27 (33.75)	Unknown	Unknown	Unknown	Unknown
84	Torregrosa et al. et al. 2014 [107]	65.2	110 (76.4)	Unknown	Unknown	Unknown	Unknown	49%	Unknown
85	Tekce et al. 2014 [108]	57.2	16 (73)	Unknown	Unknown	Unknown	Unknown	Unknown	Unknown
86	Torregrosa et al. 2012 [109] (M)	62.6	67 (75)	Unknown	Unknown	Unknown	Unknown	0%	Unknown
86	Torregrosa et al. 2012 [109] (S)	68.8	34 (74)	Unknown	Unknown	Unknown	Unknown	100%	Unknown
87	Matsui et al. 2011 [110]	73	15 (60)	6 (24%)	5 (20)	Unknown	8 (32)	Unknown	Unknown
88	Parikh et al. 2011 [111]	71	826 (68)	511 (42%)	Unknown (exclude cre > 4.5)	314 (26%)	Unknown	100%	Unknown
89	Wang 2017 [112]	58.2	54 (54.4)	Unknown	Unknown	Unknown	Unknown	100%	Unknown
90	Haase-Fielitz et al. 2009 [113]	69.5	61 (61%)	28 (28%)	27 (27%)	25 (25%)	Unknown	100%	Unknown
91	Waskowski 2021 [114]	69.4	77 (82.8)	15 (16.1)	27 (29)	14 (15.1)	No	71 (76.3)	Unknown
92	Imoto 2021 [115]	72	58 (54.7)	Unknown	Unknown	10 (9.4)	No	No	Unknown
93	Lee 2021 [116]	62	95 (66.0)	53 (36.8)	Unknown	Unknown	No	100%	Unknown
94	Szymanowicz 2021 [117]	68	57 (50)	36 (31.5)	Unknown	74 (64.9)	No	100%	Unknown
95	Zhen 2021 [118]	61.7	110 (63.9)	48 (27.9)	Unknown	Unknown	No	No	Unknown
96	Obata 2021 [119]	69.8	57 (89)	56 (87.5)	Unknown	Unknown	No	100%	Unknown
97	Qiu 2021 [120]	74.7	60 (66.7)	24 (26.7)	Unknown	Unknown	100%	No	6.0
98	Shakked 2022 [121]	52	31 (59.6)	21 (40.4)	Unknown	Unknown	No	No	Unknown
99	Vogel 2021 [122]	55	34 (63)	7 (13)	7 (13)	1 (1.9)	No	No	Unknown
100	Ergun 2021 [123]	71.6	33 (55)	Unknown	Unknown	Unknown	No	100%	Unknown
101	Pilarczyk 2022 [124]	69.1	33 (32.7)	Unknown	5 (4.9)	Unknown	No	100%	Unknown
102	Okuda 2022 [125]	75.2	33 (68.8)	9 (18.8)	12 (25)	Unknown	No	100%	Unknown
103	Pei 2022 [126]	72	97 (59.9)	49 (30.2)	17 (10.5)	43 (26.5)	100%	No	2
104	Jahaj 2021 [127]	47.2	199 (74.8)	Unknown	Unknown	Unknown	No	No	6.4
105	Garms 2021 [128]	49.6	63 (67)	27 (28.7)	5 (5.3)	Unknown	No	43 (45.7)	Unknown
106	Irqsusi 2021 [129]	68.5	50 (100)	17 (34)	8 (16)	47 (94)	No	100%	Unknown
107	Guray 2021 [130]	67.6	48 (57.1)	23 (27.3)	Unknown	Unknown	No	No	Unknown
108	Tan 2022 [131]	50.5	62 (39.5)	33 (2.1)	Unknown	Unknown	100%	100%	Unknown
109	Lakhal 2021 [132]	78.6	32 (49.2)	14 (21.5)	Unknown	Unknown	No	100%	Unknown
110	Sahu 2022 [133]	58.3	182 (85.8)	59 (27.8)	Unknown	3 (1.4)	No	No	Unknown

*SOFA* sequential organ failure assessment

All 110 studies provided quantifiable results for AKI. Seventy-nine studies exclusively enrolled ICU patients, and 31 studies enrolled non-ICU patients. Fifty-seven studies exclusively enrolled surgery patients, and 55 studies enrolled patients from mixed surgical/medical settings. Only 8 studies enrolled patients with sepsis, and therefore, analysis of sepsis was not conducted. Of the enrolled studies, 44 used the KDIGO classification as the only definition for AKI, 23 used AKIN, 21 used RIFLE, 6 used two or more definitions, 6 used a 50% increase in SCr, 1 used an increase in SCr from normal to > 3 mg/dL, 3 used a 0.5 mg/dL increase in SCr within 48–72 h, and 6 were at the discretion of the attending physicians.

### Quality of the enrolled trials

The studies were published over 18 years and varied in sample size from 22 to 1635 patients (Tables 1, 2). The QUADAS-2 tool revealed that the quality of the enrolled studies varied. There was a low and/or unclear risk in each study in most domains of bias evaluation (Additional file 1: Figs. S1, S2). The risk of bias was low for patient selection in 84 studies (76.4%); index test in 26 studies (23.6%); reference standard in 30 studies (27.3%); and flow and timing in 96 studies (87.3%). The applicability concerns were low for patient selection in 89 studies (80.9%); index test in 106 studies (96.4%); and reference standard in 95 studies (86.4%). Therefore, according to the criteria of overall quality, 70 studies (63.6%) were rated as low risk, 15 studies (13.6%) as unclear risk, and 25 studies (22.7%) as high risk.

### Primary outcomes

**Fig. 1:**
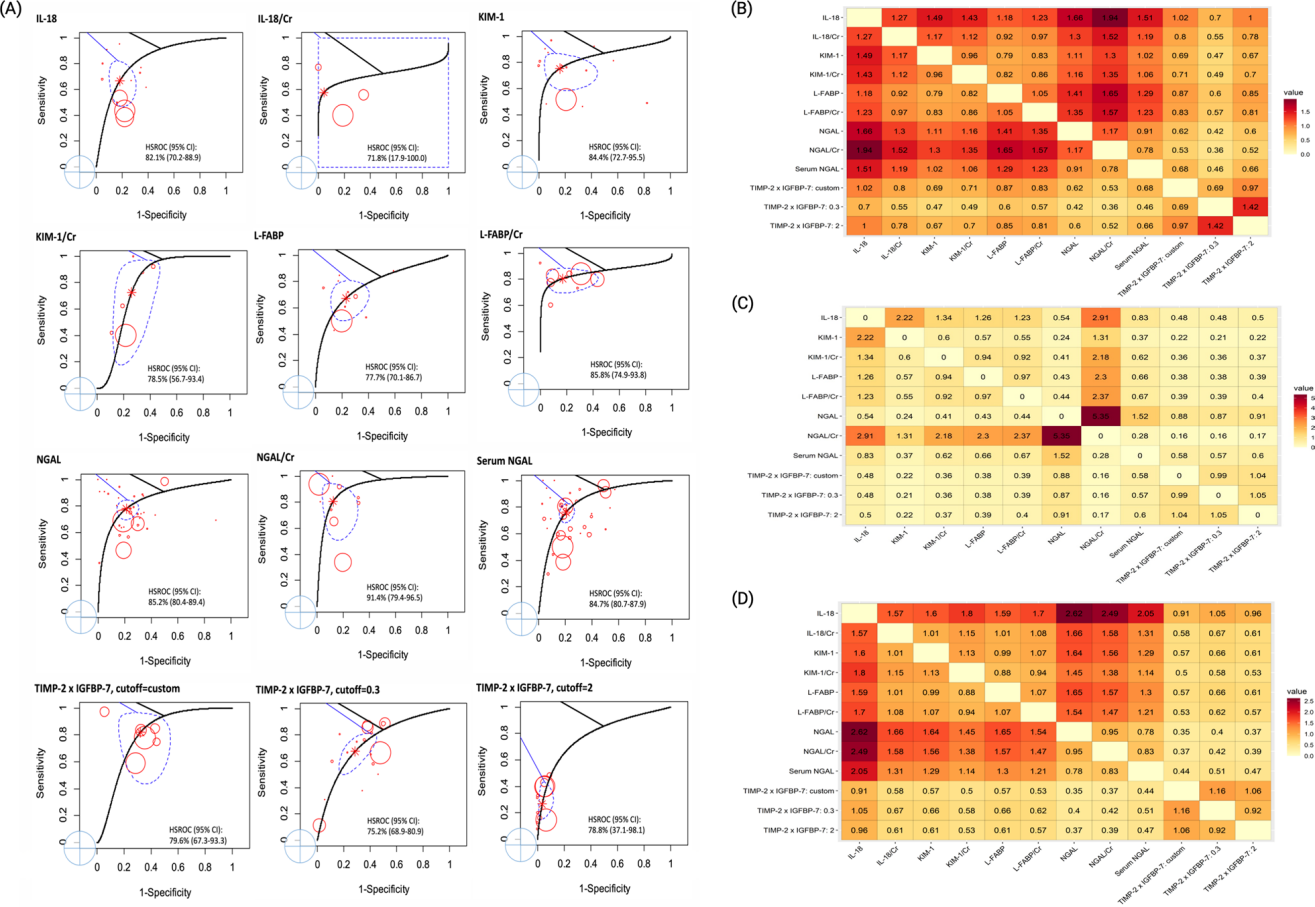
The discriminative accuracy of the biomarkers to diagnose AKI (**A**) HSROCs for the AKI biomarkers. The global HSROCs depicting the discriminative accuracy of the biomarkers to diagnose AKI. The red point represents the observation and the circle represents the sample size. The asterisk “*” represents the estimate of HSROC, and the blue dotted circle around it indicates the 95% confidence interval. Among the biomarkers, NGAL, NGAL/Cr, L-FABP/Cr, TIMP-2 × IGFBP-7: custom, and TIMP-2 × IGFBP-7: 2 had good HSROCs (> 85–90%). (**B**) Heatmap plot depicting pairwise comparisons (row vs. column) of relative DOR between the biomarkers in the whole population. The contents of the diagonal are the values of the relative DOR. Red depicts a positive DOR, while yellow depicts no correlation. NGAL and NGAL/Cr had the best relative DOR of the biomarkers. (**C**) Heatmap plot depicting pairwise comparisons (row vs. column) of relative DOR between the biomarkers in the surgical subgroup. The contents of the diagonal are the values of the relative DOR. Red depicts a positive DOR, while yellow depicts no correlation. NGAL/Cr had the best relative DOR of the biomarkers. (**D**) Heatmap plot depicting pairwise comparisons (row vs. column) of relative DOR between the markers in the studies that did not use UO criteria. The contents of the diagonal are the values of the relative DOR. Red depicts a positive DOR, while yellow depicts no correlation. NGAL had the best relative DOR of the biomarkers. Abbreviations**:** AKI, acute kidney injury; Cr, creatinine; DOR, diagnostic odds ratio; HSROC, hierarchical summary receiver operating characteristic curve; IL-18, interleukin-18; KIM-1, kidney injury molecule-1; L-FABP, liver-type fatty acid-binding protein; NGAL, neutrophil gelatinase-associated lipocalin; TIMP-2 × IGFBP-7: tissue inhibitor of metalloproteinases-2 × insulin-like growth factor-binding protein-7; and UO, urine output

The occurrence of AKI was based on all of the included studies with a total of 38,725 patients, of whom 8,340 had incident AKI. Among the 11 candidate biomarkers, the diagnostic accuracy (defined as the DOR value) was numerically highest for NGAL/creatinine (NGAL/Cr) (DOR 16.2, 95% CI 10.1–25.9), which was reported in 9 studies. The results demonstrated that urinary NGAL had high diagnostic accuracy (DOR 13.8, 95% CI 10.2–18.8), which was significantly better than IL-18 (relative DOR 0.60, 95% CI 0.44–0.82), and TIMP-2 × IGFBP-7: 0.3 (relative DOR 0.42, 95% CI 0.22–0.81) for the occurrence of AKI (Table 3). The HSROCs depicting the overall discriminative accuracy of the biomarkers to diagnose AKI are shown in Fig. 1A. Of the biomarkers, urinary NGAL (HSROC 85.2%, 95% CI 80.4–89.4%), urinary NGAL/Cr (HSROC 91.4%, 95% CI 79.4–96.5%), serum NGAL (HSROC 84.7%, 95% CI 80.7–87.9%), IL-18 (HSROC 82.1%, 95% CI 70.2–88.9%), KIM-1 (HSROC 84.4%, 95% CI 72.7–95.5%), and L-FABP/Cr (HSROC 85.8%, 95% CI 74.9–93.8%) had HSROC values greater than 80%. Additional file 1: Figs. S3, S4 and Fig. 1B illustrate the pairwise comparisons of the biomarkers for pooled sensitivity, specificity, and DOR in the whole population.

**Table 3:** Summary of the diagnostic meta-analysis in the whole population

Marker	No. of study	Sensitivity, % (95% CI)	Specificity, % (95% CI)	DOR (95% CI)	Relative sensitivity (95% CI)	Relative specificity (95% CI)	Relative DOR (95% CI)
NGAL	35	76.8 (72.3–80.8)	80.7 (77.1–83.8)	13.8 (10.2–18.8)	Reference	Reference	Reference
IL-18	12	67.6 (60.4–74.0)	80.0 (76.1–83.5)	8.4 (5.7–12.1)	0.88 (0.80–0.96)*	0.99 (0.97–1.02)	0.60 (0.44–0.82)*
IL-18/Cr	3	71.9 (63.3–79.1)	80.6 (75.0–85.3)	10.6 (6.4–17.6)	0.94 (0.84–1.04)	1.00 (0.95–1.05)	0.77 (0.48–1.23)
KIM-1	14	76.3 (70.4–81.4)	79.4 (75.2–83.1)	12.4 (8.5–18.1)	0.99 (0.93–1.06)	0.98 (0.96–1.01)	0.90 (0.65–1.23)
KIM-1/Cr	6	69.9 (60.1–78.1)	83.8 (78.8–87.7)	12.0 (7.0–20.3)	0.91 (0.80–1.03)	1.04 (0.99–1.09)	0.86 (0.52–1.43)
L-FABP	10	69.8 (62.0–76.5)	81.0 (77.0–84.4)	9.8 (6.5–14.8)	0.91 (0.83–0.998)*	1.00 (0.98–1.03)	0.71 (0.50–1.01)
L-FABP/Cr	8	81.8 (74.0–87.7)	69.6 (58.5–78.7)	10.3 (5.4–19.7)	1.07 (0.97–1.17)	0.86 (0.75–0.99)*	0.74 (0.38–1.44)
NGAL/Cr	9	71.6 (63.5–78.5)	86.5 (82.5–89.7)	16.2 (10.1–25.9)	0.93 (0.84–1.03)	1.07 (1.03–1.11)*	1.17 (0.75–1.82)
Serum NGAL	40	76.3 (71.6–80.4)	79.7 (75.9–83.0)	12.6 (9.3–17.3)	0.99 (0.94–1.05)	0.99 (0.96–1.01)	0.91 (0.69–1.21)
TIMP-2 × IGFBP-7: custom	6	86.3 (74.8–93.0)	57.6 (43.1–70.9)	8.5 (3.4–21.4)	1.12 (0.999–1.26)	0.71 (0.56–0.92)*	0.62 (0.23–1.63)
TIMP-2 × IGFBP-7: 0.3	17	68.0 (58.1–76.4)	73.5 (64.1–81.1)	5.9 (3.3–10.4)	0.88 (0.76–1.02)	0.91 (0.80–1.03)	0.42 (0.22–0.81)*
TIMP-2 × IGFBP-7: 2	11	18.5 (12.4–26.8)	97.3 (95.7–98.4)	8.3 (4.3–16.1)	0.24 (0.16–0.36)*	1.21 (1.15–1.26)*	0.60 (0.29–1.24)

*CI* confidence interval, *Cr* creatinine, *DOR* diagnostic odds ratio, *IL-18* interleukin-18, *KIM-1* kidney injury molecule-1, *L-FABP* liver-type fatty acid-binding protein, *NGAL* neutrophil gelatinase-associated lipocalin, *TIMP-2 × IGFBP-7* tissue inhibitor of metalloproteinases-2 × insulin-like growth factor-binding protein-7

*Numbers in bold indicate significant difference (*P* < 0.05) versus the referent category: “NGAL”

### Subgroup analyses

In the setting of ICU patients, the diagnostic accuracy was numerically highest for NGAL/Cr (DOR 12.6, 95% CI 7.8–20.2), followed by L-FABP/Cr and urinary NGAL. The diagnostic accuracy of urinary NGAL was significantly better than TIMP-2 × IGFBP-7: 0.3 (relative DOR 0.51, 95% CI 0.28–0.92) (upper panel in Table 4). In contrast, urinary NGAL (DOR 17.1, 95% CI 7.8–37.5), urinary NGAL/Cr (DOR 99.3, 95% CI 7.7–1285.0), and serum NGAL (DOR 15.0, 95% CI 7.1–32.0) had better diagnostic accuracy for AKI than IL-18 (DOR 9.6, 95% CI 4.2–21.9) in the non-ICU patients (lower panel in Table 4). Additional file 1: Figs. S5–S7 illustrate the pairwise comparisons of the biomarkers for pooled sensitivity, specificity, and DOR in the ICU patients.

**Table 4:** Summary of the diagnostic meta-analysis in the ICU and non-ICU population

Population/marker	No. of study	Sensitivity, % (95% CI)	Specificity, % (95% CI)	DOR (95% CI)	Relative sensitivity (95% CI)	Relative specificity (95% CI)	Relative DOR (95% CI)
*ICU population*							
NGAL	27	76.2 (71.0–80.7)	78.6 (74.3–82.3)	11.8 (8.6–16.1)	Reference	Reference	Reference
IL-18	8	65.4 (55.3–74.2)	80.4 (75.7–84.3)	7.7 (4.8–12.3)	0.86 (0.75–0.98)*	1.02 (0.99–1.06)	0.66 (0.42–1.02)
IL-18/Cr	3	69.0 (59.3–77.2)	79.5 (73.3–84.5)	8.6 (5.2–14.4)	0.91 (0.80–1.02)	1.01 (0.95–1.07)	0.73 (0.45–1.20)
KIM-1	7	74.1 (65.0–81.5)	77.7 (71.3–83.1)	10.0 (5.9–16.8)	0.97 (0.87–1.08)	0.99 (0.93–1.05)	0.85 (0.52–1.40)
KIM-1/Cr	4	65.8 (55.0–75.2)	83.8 (78.5–88.0)	9.9 (5.8–17.1)	0.86 (0.75–1.001)	1.07 (1.01–1.12)*	0.85 (0.50–1.43)
L-FABP	9	69.4 (59.8–77.5)	80.2 (74.5–84.9)	9.2 (5.6–15.0)	0.91 (0.81–1.03)	1.02 (0.97–1.08)	0.78 (0.48–1.27)
L-FABP/Cr	6	84.0 (74.3–90.6)	69.6 (58.0–79.2)	12.1 (5.8–25.1)	1.10 (0.99–1.23)	0.89 (0.76–1.03)	1.03 (0.48–2.21)
NGAL/Cr	7	68.1 (59.1–76.0)	85.5 (81.0–89.1)	12.6 (7.8–20.2)	0.89 (0.80–1.01)	1.09 (1.04–1.14)*	1.07 (0.68–1.69)
Serum NGAL	26	75.3 (69.8–80.0)	78.2 (73.8–82.1)	11.0 (8.0–15.1)	0.99 (0.92–1.06)	1.00 (0.97–1.02)	0.93 (0.69–1.26)
TIMP-2 × IGFBP-7: custom	5	89.8 (79.0–95.3)	57.5 (43.0–70.9)	11.9 (4.5–31.1)	1.18 (1.06–1.31)*	0.73 (0.57–0.94)*	1.01 (0.37–2.79)
TIMP-2 × IGFBP-7: 0.3	15	67.9 (57.9–76.5)	73.9 (64.8–81.3)	6.0 (3.6–10.0)	0.89 (0.77–1.04)	0.94 (0.83–1.06)	0.51 (0.28–0.92)*
TIMP-2 × IGFBP-7: 2	9	18.1 (11.9–26.6)	97.4 (95.7–98.4)	8.1 (4.3–15.3)	0.24 (0.16–0.36)	1.24 (1.18–1.31)	0.69 (0.34–1.40)
*Non-ICU population*							
NGAL	8	75.8 (65.0–84.1)	84.5 (76.4–90.2)	17.1 (7.8–37.5)	Reference	Reference	Reference
IL-18	4	68.2 (54.8–79.1)	81.7 (72.3–88.4)	9.6 (4.2–21.9)	0.90 (0.79–1.03)	0.97 (0.93–0.999)*	0.56 (0.35–0.91)*
KIM-1	7	77.4 (66.2–85.7)	82.4 (73.2–88.9)	16.0 (7.0–36.2)	1.02 (0.92–1.13)	0.97 (0.94–1.01)	0.93 (0.58–1.51)
KIM-1/Cr	2	92.0 (50.2–99.2)	58.8 (34.4–79.5)	16.4 (1.1–237.5)	1.21 (0.96–1.53)	0.70 (0.46–1.04)	0.96 (0.06–14.79)
L-FABP/Cr	2	75.7 (46.1–91.9)	92.1 (68.5–98.4)	36.0 (3.7–349.5)	0.999 (0.71–1.40)	1.09 (0.93–1.27)	2.11 (0.19–23.38)
NGAL/Cr	2	93.5 (64.1–99.1)	87.4 (65.1–96.3)	99.3 (7.7–1285.0)	1.23 (1.02–1.49)*	1.03 (0.86–1.24)	5.81 (0.41–83.40)
Serum NGAL	14	77.2 (67.5–84.7)	81.6 (72.7–88.1)	15.0 (7.1–32.0)	1.02 (0.87–1.19)	0.97 (0.87–1.07)	0.88 (0.35–2.20)
TIMP-2 × IGFBP-7: 0.3	2	73.0 (42.4–90.9)	61.2 (26.3–87.5)	4.3 (0.5–36.4)	0.96 (0.66–1.40)	0.72 (0.40–1.30)	0.25 (0.03–2.45)
TIMP-2 × IGFBP-7: 2	2	25.9 (8.6–56.5)	95.6 (82.2–99.0)	7.6 (0.8–67.9)	0.34 (0.13–0.91)*	1.13 (1.02–1.26)*	0.44 (0.04–4.56)

*CI* confidence interval, *Cr* creatinine, *DOR* diagnostic odds ratio, *ICU* intensive care unit, *IL-18* interleukin-18, *KIM-1* kidney injury molecule-1, *L-FABP* liver-type fatty acid-binding protein, *NGAL* neutrophil gelatinase-associated lipocalin, *TIMP-2 × IGFBP-7* tissue inhibitor of metalloproteinases-2 × insulin-like growth factor-binding protein-7

*Numbers in bold indicate significant difference (*P* < 0.05) versus the referent category: “NGAL”

On the other hand, urinary NGAL had the highest diagnostic accuracy (DOR 17.9, 95% CI 12.3–26.3), which was significantly better than IL-18 (relative DOR 0.31, 95% CI 0.21–0.47), IL-18/Cr (relative DOR 0.56, 95% CI 0.34–0.94), KIM-1 (relative DOR 0.57, 95% CI 0.40–0.82), L-FABP (relative DOR 0.46, 95% CI 0.30–0.71), and TIMP-2 × IGFBP-7: 0.3 (relative DOR 0.28, 95% CI 0.10–0.79) for the occurrence of AKI in the setting of medical/mixed patients (upper panel in Table 5). Furthermore, urinary NGAL had a low diagnostic accuracy in the setting of surgical patients. Urinary NGAL/Cr (DOR 34.3, 95% CI 9.0–130.6), KIM-1 (DOR 26.2, 95% CI 9.6–71.6), L-FABP (DOR 14.9, 95% CI 7.0–31.5), and IL-18 (DOR 11.8, 95% CI 6.1–22.9) had better diagnostic accuracy than urinary NGAL (lower panel in Table 5). Additional file 1: Figs. S8–S12 and Fig. 1C illustrate the pairwise comparisons of the biomarkers for pooled sensitivity, specificity, and DOR in the medical/mixed and surgical patients.

**Table 5:** Summary of the diagnostic meta-analysis in the medical/mixed and surgical population

Population/marker	No. of study	Sensitivity, % (95% CI)	Specificity, % (95% CI)	DOR (95% CI)	Relative sensitivity (95% CI)	Relative specificity (95% CI)	Relative DOR (95% CI)
*Medical/mixed population*							
NGAL	22	80.0 (74.7–84.4)	81.8 (77.3–85.5)	17.9 (12.3–26.3)	Reference	Reference	Reference
IL-18	7	61.0 (51.3–69.9)	78.3 (72.8–82.9)	5.6 (3.5–9.0)	0.76 (0.67–0.87)	0.96 (0.92–0.99)*	0.31 (0.21–0.47)*
IL-18/Cr	3	71.6 (62.0–79.6)	80.0 (73.4–85.3)	10.1 (5.8–17.6)	0.90 (0.80–1.00)	0.98 (0.92–1.04)	0.56 (0.34–0.94)*
KIM-1	10	73.8 (66.3–80.2)	78.5 (73.0–83.0)	10.3 (6.6–16.0)	0.92 (0.85–1.00)*	0.96 (0.93–0.99)*	0.57 (0.40–0.82)*
KIM-1/Cr	4	69.7 (58.5–78.9)	82.2 (75.5–87.3)	10.6 (5.7–19.5)	0.87 (0.76–1.00)*	1.01 (0.95–1.07)	0.59 (0.33–1.05)
L-FABP	4	68.3 (57.9–77.2)	79.3 (73.9–83.8)	8.3 (4.9–13.9)	0.85 (0.75–0.97)*	0.97 (0.94–1.00)	0.46 (0.30–0.71)*
L-FABP/Cr	3	80.9 (68.7–89.1)	68.2 (41.8–86.4)	9.1 (2.6–31.5)	1.01 (0.89–1.15)	0.83 (0.59–1.18)	0.50 (0.14–1.80)
NGAL/Cr	6	71.4 (61.9–79.3)	86.0 (81.0–89.7)	15.3 (8.9–26.2)	0.89 (0.80–1.00)*	1.05 (1.01–1.10)*	0.85 (0.52–1.39)
Serum NGAL	27	77.5 (71.7–82.3)	80.4 (75.7–84.4)	14.1 (9.6–20.8)	0.97 (0.91–1.03)	0.98 (0.95–1.02)	0.79 (0.56–1.11)
TIMP-2 × IGFBP-7: 0.3	6	70.9 (54.0–83.5)	67.6 (49.7–81.5)	5.1 (2.0–13.2)	0.89 (0.71–1.11)	0.83 (0.65–1.06)	0.28 (0.10–0.79)*
TIMP-2 × IGFBP-7: 2	4	25.6 (13.7–42.6)	96.6 (92.8–98.5)	9.8 (3.5–27.2)	0.32 (0.18–0.57)*	1.18 (1.12–1.25)	0.55 (0.18–1.63)
*Surgical population*							
NGAL	13	67.5 (57.9–75.9)	75.5 (68.2–81.6)	6.4 (3.7–11.2)	Reference	Reference	Reference
IL-18	5	76.1 (65.0–84.5)	78.8 (71.7–84.5)	11.8 (6.1–22.9)	1.13 (0.98–1.29)	1.04 (0.999–1.09)	1.84 (1.08–3.13)*
KIM-1	4	85.8 (72.4–93.3)	81.3 (71.7–88.2)	26.2 (9.6–71.6)	1.27 (1.09–1.49)*	1.08 (0.98–1.18)	4.09 (1.56–10.73)*
KIM-1/Cr	2	71.8 (43.8–89.3)	86.1 (77.5–91.7)	15.7 (4.2–59.3)	1.06 (0.75–1.50)	1.14 (1.05–1.24)*	2.45 (0.66–9.13)
L-FABP	6	68.8 (55.6–79.6)	87.1 (80.6–91.6)	14.9 (7.0–31.5)	1.02 (0.84–1.23)	1.15 (1.07–1.24)*	2.32 (1.12–4.81)*
L-FABP/Cr	5	81.6 (69.4–89.7)	76.5 (63.9–85.7)	14.5 (5.8–36.2)	1.21 (1.02–1.43)*	1.01 (0.88–1.17)	2.26 (0.86–5.93)
NGAL/Cr	3	78.1 (56.1–90.9)	90.6 (80.5–95.7)	34.3 (9.0–130.6)	1.16 (0.90–1.49)	1.20 (1.08–1.33)*	5.35 (1.35–21.17)*
Serum NGAL	13	74.9 (65.9–82.2)	76.6 (69.3–82.5)	9.8 (5.5–17.4)	1.11 (0.97–1.27)	1.01 (0.97–1.06)	1.52 (0.91–2.55)
TIMP-2 × IGFBP-7: custom	5	81.5 (66.4–90.8)	56.1 (39.8–71.1)	5.6 (2.0–16.1)	1.21 (0.99–1.48)	0.74 (0.55–1.005)	0.88 (0.27–2.88)
TIMP-2 × IGFBP-7: 0.3	11	65.7 (53.1–76.3)	74.5 (62.6–83.6)	5.6 (2.6–12.2)	0.97 (0.78–1.22)	0.99 (0.84–1.17)	0.87 (0.34–2.27)
TIMP-2 × IGFBP-7: 2	7	13.9 (7.9–23.2)	97.3 (94.8–98.6)	5.8 (2.3–15.0)	0.21 (0.12–0.36)	1.29 (1.18–1.41)	0.91 (0.31–2.72)

*CI* confidence interval, *DOR* diagnostic odds ratio, *NGAL* neutrophil gelatinase-associated lipocalin, *IL-18* interleukin-18, *Cr* urine creatinine, *KIM-1* kidney injury molecule-1, *L-FABP* liver-type fatty acid-binding protein, *TIMP-2 × IGFBP-7* tissue inhibitor of metalloproteinases-2 × insulin-like growth factor-binding protein-7

*Numbers in bold indicate significant difference (*P* < 0.05) versus the referent category: “NGAL”

Only twelve studies recruited patients with sepsis, and therefore analysis of sepsis was not conducted. The results of the non-sepsis patients were similar to those of the overall cohort: Urinary NGAL (DOR 16.3, 95% CI 11.8–22.4) had significantly better diagnostic accuracy for AKI than IL-18 (relative DOR 0.52, 95% CI 0.37–0.72), L-FABP (relative DOR 0.65, 95% CI 0.46–0.93), and TIMP-2 × IGFBP-7: 0.3 (relative DOR 0.36, 95% CI 0.19–0.67) (Additional file 1: Table S1). Additional file 1: Figs. S13–S15 illustrate the pairwise comparisons of the biomarkers for pooled sensitivity, specificity, and DOR in the non-sepsis patients.

Only 10 studies recruited patients without using standard AKI criteria (RIFLE/AKIN/KDIGO), and therefore, the analysis was not conducted. In the 100 studies which adopted standard AKI criteria, NGAL/Cr had the highest diagnostic accuracy (DOR 15.4, 95% CI 9.6–24.4), followed by KIM-1 (DOR 12.8, 95% CI 8.7–18.7), and urinary NGAL (DOR 12.5, 95% CI 9.2–16.9). Urinary NGAL had significantly better diagnostic accuracy for AKI than IL-18 (relative DOR 0.62, 95% CI 0.45–0.85) and TIMP-2 × IGFBP-7: 0.3 (relative DOR 0.46, 95% CI 0.24–0.86) (Table 6). Additional file 1: Figs. S16–S18 illustrate the pairwise comparisons of the biomarkers for pooled sensitivity, specificity, and DOR in the studies using standard AKI criteria.

**Table 6:** Summary of the diagnostic meta-analysis for the studies using standard AKI criteria (any of RIFLE, AKIN, and KDIGO)

Marker	No. of study	Sensitivity, % (95% CI)	Specificity, % (95% CI)	DOR (95% CI)	Relative sensitivity (95% CI)	Relative specificity (95% CI)	Relative DOR (95% CI)
NGAL	33	75.9 (71.2–80.0)	79.9 (76.0–83.3)	12.5 (9.2–16.9)	Reference	Reference	Reference
IL-18	11	66.2 (58.9–72.8)	79.8 (75.7–83.4)	7.7 (5.3–11.2)	0.87 (0.79–0.96)*	1.00 (0.98–1.02)	0.62 (0.45–0.85)*
IL-18/Cr	3	71.4 (62.8–78.6)	80.1 (74.3–84.9)	10.0 (6.1–16.5)	0.94 (0.84–1.05)	1.00 (0.95–1.06)	0.80 (0.50–1.29)
KIM-1	12	76.2 (70.2–81.4)	80.0 (75.6–83.7)	12.8 (8.7–18.7)	1.01 (0.94–1.08)	1.00 (0.97–1.03)	1.03 (0.74–1.42)
KIM-1/Cr	6	69.3 (59.5–77.5)	83.4 (78.3–87.5)	11.3 (6.7–19.1)	0.91 (0.80–1.04)	1.04 (1.00–1.09)	0.91 (0.55–1.50)
L-FABP	9	70.4 (62.6–77.1)	81.7 (77.7–85.2)	10.6 (7.0–16.1)	0.93 (0.84–1.02)	1.02 (1.00–1.05)	0.85 (0.59–1.22)
L-FABP/Cr	8	81.9 (74.2–87.7)	70.0 (59.0–79.1)	10.6 (5.6–20.1)	1.08 (0.99–1.18)	0.88 (0.76–1.01)	0.85 (0.44–1.63)
NGAL/Cr	9	71.1 (63.0–78.1)	86.2 (82.1–89.5)	15.4 (9.6–24.4)	0.94 (0.85–1.04)	1.08 (1.04–1.12)*	1.23 (0.79–1.91)
Serum NGAL	35	74.3 (69.4–78.8)	78.9 (74.8–82.5)	10.8 (7.9–14.8)	0.98 (0.92–1.04)	0.99 (0.96–1.01)	0.87 (0.65–1.15)
TIMP-2 × IGFBP-7: custom	6	85.9 (74.4–92.7)	58.1 (43.6–71.4)	8.4 (3.4–20.7)	1.13 (1.00–1.28)*	0.73 (0.57–0.93)*	0.67 (0.26–1.75)
TIMP-2 × IGFBP-7: 0.3	16	66.6 (56.7–75.2)	74.0 (64.5–81.7)	5.7 (3.2–10.0)	0.88 (0.75–1.02)	0.93 (0.82–1.05)	0.46 (0.24–0.86)*
TIMP-2 × IGFBP-7: 2	10	17.5 (11.6–25.6)	97.5 (95.8–98.5)	8.3 (4.2–16.1)	0.23 (0.15–0.35)	1.22 (1.16–1.28)	0.66 (0.32–1.38)

*AKI* acute kidney injury, *RIFLE* Risk, Injury, Failure, Loss, and End-stage renal disease, *AKIN* Acute Kidney Injury Network, *KDIGO* Kidney Disease Improving Global Outcomes, *CI* confidence interval, *DOR* diagnostic odds ratio, *NGAL* neutrophil gelatinase-associated lipocalin, *IL-18* interleukin-18, *Cr* urine creatinine, *KIM-1* kidney injury molecule-1, *L-FABP* liver-type fatty acid-binding protein; TIMP-2 × IGFBP-7, tissue inhibitor of metalloproteinases-2 × insulin-like growth factor-binding protein-7;

*Numbers in bold indicate significant difference (*P* < 0.05) versus the referent category: “NGAL”

Only 30 studies diagnosed AKI using urine output criteria, and the diagnostic accuracy was numerically highest for KIM-1 (DOR 14.6, 95% CI 5.9–35.9), followed by IL-18 (DOR 13.1, 95% CI 6.7–25.7), and TIMP-2 × IGFBP-7: 2 (DOR 12.0, 95% CI 5.2–27.8). Among the other 80 studies that diagnosed AKI without using urine output criteria, NGAL had the highest diagnostic accuracy (DOR 18.6, 95% CI 12.8–27.0), followed by urinary NGAL/Cr (DOR 17.6, 95% CI 10.7–29.1). Urinary NGAL had significantly better diagnostic accuracy for AKI than IL-18 (relative DOR 0.38, 95% CI 0.26–0.56), IL-18/Cr (relative DOR 0.60, 95% CI 0.37–0.98), KIM-1 (relative DOR 0.61, 95% CI 0.42–0.88), and L-FABP (relative DOR 0.61, 95% CI 0.41–0.88) (Table 7). Additional file 1: Figs. S19–S20 and Fig. 1D illustrate the pairwise comparisons of the biomarkers for pooled sensitivity, specificity, and DOR in the studies that did not use urine output criteria.

**Table 7:** Summary of the diagnostic meta-analysis according to AKI criteria with or without UO

Population/marker	No. of study	Sensitivity, % (95% CI)	Specificity, % (95% CI)	DOR (95% CI)	Relative sensitivity (95% CI)	Relative specificity (95% CI)	Relative DOR (95% CI)
*Non-UO*							
NGAL	27	81.1 (76.6–84.9)	81.3 (77.2–84.7)	18.6 (12.8–27.0)	Reference	Reference	Reference
IL-18	9	63.7 (55.1–71.6)	80.1 (75.5–84.1)	7.1 (4.5–11.2)	0.79 (0.70–0.89)*	0.99 (0.96–1.02)	0.38 (0.26–0.56)*
IL-18/Cr	3	72.4 (63.8–79.6)	81.0 (75.2–85.7)	11.2 (6.6–19.0)	0.89 (0.80–0.99)*	1.00 (0.95–1.05)	0.60 (0.37–0.98)*
KIM-1	12	73.8 (67.0–79.7)	80.1 (75.4–84.0)	11.3 (7.3–17.5)	0.91 (0.84–0.99)*	0.99 (0.96–1.01)	0.61 (0.42–0.88)*
KIM-1/Cr	6	70.8 (61.2–78.8)	84.1 (79.0–88.2)	12.8 (7.3–22.3)	0.87 (0.77–0.99)*	1.04 (0.99–1.08)	0.69 (0.41–1.16)
L-FABP	9	72.2 (64.2–79.0)	81.2 (76.7–85.0)	11.2 (7.0–18.0)	0.89 (0.81–0.98)*	1.00 (0.97–1.03)	0.61 (0.41–0.88)*
L-FABP/Cr	6	80.3 (70.4–87.4)	74.8 (59.4–85.8)	12.1 (4.9–29.7)	0.99 (0.89–1.11)	0.92 (0.77–1.10)	0.65 (0.26–1.64)
NGAL/Cr	9	72.9 (65.0–79.6)	86.8 (82.6–90.0)	17.6 (10.7–29.1)	0.90 (0.82–0.99)*	1.07 (1.03–1.11)*	0.95 (0.60–1.50)
Serum NGAL	34	79.0 (74.3–83.1)	79.5 (75.1–83.3)	14.6 (10.0–21.2)	0.97 (0.92–1.03)	0.98 (0.95–1.01)	0.78 (0.56–1.09)
TIMP-2 × IGFBP-7: 0.3	5	82.2 (67.8–91.0)	61.8 (41.3–78.9)	7.5 (2.3–24.6)	1.01 (0.87–1.18)	0.76 (0.55–1.05)	0.40 (0.12–1.40)
TIMP-2 × IGFBP-7: 2	5	25.4 (13.7–42.2)	95.3 (89.4–98.0)	6.8 (2.1–22.8)	0.31 (0.18–0.55)*	1.17 (1.10–1.25)*	0.37 (0.10–1.30)
*UO*							
NGAL	7	68.2 (54.7–79.2)	78.5 (67.8–86.3)	7.8 (4.6–13.1)	Reference	Reference	Reference
IL-18	2	77.4 (62.9–87.4)	79.3 (68.5–87.1)	13.1 (6.7–25.7)	1.14 (0.98–1.31)	1.01 (0.97–1.05)	1.68 (0.94–3.01)
KIM-1	2	84.9 (71.6–92.6)	72.2 (55.1–84.6)	14.6 (5.9–35.9)	1.25 (1.08–1.44)*	0.92 (0.79–1.08)	1.87 (0.81–4.31)
L-FABP/Cr	2	70.4 (38.1–90.2)	77.5 (46.5–93.2)	8.2 (2.4–28.2)	1.03 (0.67–1.60)	0.99 (0.71–1.38)	1.05 (0.27–4.02)
Serum NGAL	6	67.8 (53.3–79.6)	79.2 (68.6–86.8)	8.0 (4.5–14.1)	1.00 (0.83–1.19)	1.01 (0.97–1.05)	1.03 (0.57–1.84)
TIMP-2 × IGFBP-7: custom	5	88.2 (76.1–94.6)	55.8 (39.1–71.2)	9.5 (4.0–22.6)	1.29 (1.05–1.60)*	0.71 (0.52–0.98)*	1.21 (0.44–3.36)
TIMP-2 × IGFBP-7: 0.3	12	59.0 (46.3–70.6)	77.2 (66.8–85.1)	4.9 (3.0–7.9)	0.87 (0.65–1.14)	0.98 (0.83–1.16)	0.63 (0.31–1.27)
TIMP-2 × IGFBP-7: 2	6	16.7 (9.6–27.4)	98.4 (96.5–99.3)	12.0 (5.2–27.8)	0.24 (0.14–0.43)*	1.25 (1.11–1.41)*	1.54 (0.57–4.13)

*CI* confidence interval, *DOR* diagnostic odds ratio, *NGAL* neutrophil gelatinase-associated lipocalin, *IL-18* interleukin-18, *Cr* urine creatinine, *KIM-1* kidney injury molecule-1, *L-FABP* liver-type fatty acid-binding protein, *TIMP-2* × *IGFBP-7* tissue inhibitor of metalloproteinases-2 × insulin-like growth factor-binding protein-7, *UO* urine output

*Numbers in bold indicate significant difference (*P* < 0.05) versus the referent category: “NGAL”

### Sensitivity analyses

To determine the robustness of the study results, we examined the extent to which the results were influenced by the quality of the enrolled study, the economic situation of the countries in which they were conducted, and the definition of the study outcome.

We first stratified the studies according to their quality. Seventy studies were of high quality and 40 studies were of low or middle quality. Among the high-quality studies, the diagnostic accuracy was numerically highest for urinary NGAL (DOR 12.95, 95% CI 8.88–18.87), followed by urinary NGAL/Cr (DOR 12.34, 95% CI 5.85–26.02), and serum NGAL (DOR 12.32, 95% CI 8.41–18.06). Urinary NGAL had significantly better diagnostic accuracy for AKI than IL-18 (relative DOR 0.56, 95% CI 0.39–0.78), L-FABP (relative DOR 0.66, 95% CI 0.45–0.97), and TIMP-2 × IGFBP-7: 0.3 (relative DOR 0.43, 95% CI 0.22–0.87). Among the low- or middle-quality studies, KIM-1/Cr had the highest diagnostic accuracy (DOR 35.33, 95% CI 9.87–126.47), followed by KIM-1 (DOR 34.60, 95% CI 17.16–69.77), and IL-18 (DOR 30.43, 95% CI 12.80–72.33). Both KIM-1 (relative DOR 3.00, 95% CI 1.53–5.87) and IL-18 (relative DOR 2.64, 95% CI 1.11–6.28) had significantly better diagnostic accuracy for AKI than NGAL, while IL-18/Cr had significantly worse diagnostic accuracy for AKI than NGAL (relative DOR 0.42, 95% CI 0.22–0.81) (Additional file 1: Table S2).

Seventy-eight studies were conducted in high-income countries, and the diagnostic accuracy was numerically highest for urinary NGAL/Cr (DOR 15.23, 95% CI 9.56–24.26), and urinary NGAL (DOR 14.13, 95% CI 10.03–19.89). Urinary NGAL had significantly better diagnostic accuracy for AKI than IL-18 (relative DOR 0.46, 95% CI 0.33–0.64), L-FABP (relative DOR 0.54, 95% CI 0.36–0.79), and TIMP-2 × IGFBP-7: 0.3 (relative DOR 0.40, 95% CI 0.21–0.74). Among the other 32 studies conducted in middle- or low-income countries, L-FABP had the highest diagnostic accuracy (DOR 45.15, 95% CI 14.56–140.05), which was significantly better than urinary NGAL (relative DOR 2.89, 95% CI 1.12–7.42) (Additional file 1: Table S3).

Thirty-seven studies focused on early onset AKI (AKI developed within 48 h), and the diagnostic accuracy was numerically highest for L-FABP (DOR 33.1, 95% CI 11.5–95.1), serum NGAL (DOR 21.4, 95% CI 10.5–43.7), L-FABP/Cr (DOR 21.4, 95% CI 2.9–158.8), and urinary NGAL (DOR 15.4, 95% CI 7.2–32.9) (Additional file 1: Table S4).

Twenty-four studies focused on severe AKI (AKI stage 2 or 3), and the diagnostic accuracy was numerically highest for TIMP-2 × IGFBP-7: custom (DOR 19.6, 95% CI 7.0–55.3), and serum NGAL (DOR 11.5, 95% CI 6.1–21.9) (Additional file 1: Table S5). Ten studies focused on renal replacement therapy, and both urinary NGAL (DOR 15.2, 95% CI 5.3–43.5) and serum NGAL (DOR 12.1, 95% CI 4.7–31.1) had good diagnostic accuracy (Additional file 1: Table S6).

The findings were not materially different from the standard analysis and remained robust in the sensitivity analyses.

### Publication bias

Publication bias was assessed visually using funnel plots. There were apparent asymmetrical patterns in the funnel plots for all the biomarkers except TIMP-2 × IGFBP-7: custom, TIMP-2 × IGFBP-7: 0.3, and TIMP-2 × IGFBP-7: 2.0. These results suggested that publication bias was obvious in this meta-analysis (Additional file 1: Appendix).

### Assessment of quality of evidence and summary of findings

The quality of evidence was assessed using the GRADE system. We evaluated the primary outcomes and presented them as summary of findings in Additional file 1: Appendix.

## Discussion

The current study is the most comprehensive systematic review to date including the highest number of studies of candidate AKI biomarkers. In this systematic review of 110 studies including 38,725 patients, the overall AKI rate was 21.5% (8340/38725). Serum NGAL and urinary NGAL were the most commonly used biomarkers for AKI (Table 3). In the whole population, both serum and urine NGAL had the best diagnostic accuracy regardless of whether or not they were adjusted by urinary creatinine (Table 3). For the critical patients, all of the biomarkers had similar predictive performance for AKI (upper panel in Table 4). However, for the non-critical patients, NGAL, NGAL/Cr, and serum NGAL had better diagnostic accuracy for AKI than IL-18 (lower panel in Table 4). In the medical patients, NGAL had the best diagnostic accuracy (upper panel in Table 5), while in the surgical patients, NGAL/Cr and KIM-1 had the best diagnostic accuracy (lower panel in Table 5). Our data showed that NGAL/Cr had the best predictive performance when using a HSROC meta-analysis approach.

There is an unmet need for the early detection of AKI due to an increase in the incidence of AKI in hospitalized patients [134, 135]. In clinical practice, it is difficult to recognize AKI before the level of creatinine changes, at which time the damage may be irreversible [4]. Therefore, researchers are increasingly interested in identifying biomarkers that can identify AKI at an early stage. The 23rd ADQI consensus meeting proposed combining clinical assessments, traditional tests, and validated novel biomarkers to identify patients at risk of AKI [136]. In susceptible patients exposed to high-risk events, biomarkers can predict the development or progression of AKI and may guide targeted therapy [137]. In the literature, many biomarkers have performed better than SCr when histologic evidence of kidney injury was used as the reference standard [138]. Although various biomarkers have been associated with AKI and adverse outcomes, the clinical application of any single biomarker has failed to demonstrate troponin-like diagnostic performance in myocardial infarction. The Translational Research Investigating Biomarker Endpoints in AKI (TRIBE-AKI) study [37, 111, 139] showed the heterogeneity of AKI subtype is a major limitation for large-scale population studies. In the present study, we demonstrated that several biomarkers had good predictive performance for AKI. In addition, the damage biomarkers had better predictive ability for AKI than the stress biomarker in various clinical settings. It is likely that the ability to identify different etiologies, mechanisms, and types of AKI will be critical in developing targeted therapies and designing pharmacological trials to enable more precise medicine or therapeutic interventions.

The complexity of the pathogenesis of AKI due to factors such as hemodynamics, inflammatory status, genetic background, the use of nephrotoxic compounds, and interventions means that the clinical course of AKI differs in different clinical situations [140]. In critically ill or surgical patients, the potential benefits of reducing kidney injury-related complications may outweigh the loss caused by over-monitoring the patient, such as related length of stay. Appropriate biomarkers should improve the detection rate of AKI with high sensitivity and good negative predictive value, thus enabling timely initiation of preventive strategies for AKI [141]. Previous investigations have reported that TIMP-2 × IGFBP-7 was a good biomarker to identify patients who will develop AKI and reduce the need for renal replacement therapy [136, 137, 142]. As demonstrated in the present study, NGAL/Cr, L-FABP/Cr, and TIMP-2 × IGFBP-7: custom seemed to have good predictive performance in the setting of critically ill patients, while NGAL/Cr and KIM-1 were the best biomarkers in surgical patients (Tables 4, 5).

In non-critically ill or medical patients, patient stratification for the risk of AKI should be applied to the entire hospital population before any scheduled elective intervention. In order to minimize unnecessary impacts due to these scheduled treatments, the specificity should outweigh the sensitivity [141]. In our study, the clinical performance of TIMP-2 × IGFBP-7 with a cutoff value of 2 was significantly better than that of TIMP-2 × IGFBP-7 with a cutoff value of 0.3 in the medical patients. Urinary NGAL, KIM-1, and serum NGAL seemed to be the best biomarkers in the setting of non-critically ill patients and medical patients (Tables 4, 5).

However, the sensitivity and specificity in the enrolled studies were heterogeneous because they depended on the circumstances and the threshold effects of the biomarkers. Considering the potential threshold effects and the correlation between sensitivity and specificity, HSROC analysis proved the good predictive performance of L-FABP/Cr and the NGAL series (Fig. 1A). There were differences in the applied diagnostic criteria for AKI between the enrolled studies. The subgroup analysis also demonstrated that the relative diagnostic accuracy of the AKI biomarkers remained consistent in the studies using current standard AKI criteria (RIFLE/AKIN/KDIGO) (Table 6). NGAL series seemed to have the best predictive performance for AKI, especially in the high-quality studies and in the studies which were conducted in high-income countries. Other biomarkers outperformed the NGAL series only in low- or moderate-quality studies or in the studies conducted in middle- or low-income countries (Additional file 1: Tables S2-S3). Sensitivity analysis also demonstrated the good predictive performance of serum NGAL, urinary NGAL, and TIMP-2 × IGFBP-7: custom for early onset AKI (AKI developed within 48 h) and severe AKI (stage 2–3 or renal replacement therapy) (Additional file 1: Tables S4-S6). These findings enhance the robustness of the study results.

Although the damage and stress biomarkers in this study had good predictive performance, unlike troponin in acute coronary syndrome, none of the reported biomarkers are completely specific for AKI. Previous studies have reported that NGAL, IL-18, and KIM-1 may be elevated in the setting of sepsis and CKD [143–146]. Of note, these biomarkers can be used to recruit more homogenous patient populations when implementing a clinical trial [147]. Biomarkers to identify and characterize AKI sub-types are necessary and may have the potential to provide individualized timely etiology-based management of AKI. In addition, considering the complex and multifactorial etiology of AKI, a panel of multiple biomarkers including stress, injury, and kidney reserve biomarkers could provide better discrimination for AKI. Furthermore, more kidney tissue-specific markers may help localize and quantify the severity of AKI and provide a deeper understanding of the pathophysiology of AKI. These biomarkers may offer opportunities for personalized management of AKI and support the call for a refinement of the existing AKI criteria.

### Strengths and limitations

The strength of our analysis is the extensive literature search of related studies. We used standard Cochrane protocols and included the largest cumulative study sample size to date in comparison with previous reports. The strength of our meta-analysis also lies in the comprehensive data search with subgroup analyses across several clinical scenarios. We used the GRADE approach to rate the certainty of evidence [148].

Besides limitations in the meta-analysis, there were several limitations in the individual studies. First, most studies had a small sample size, and this contributed to the high heterogeneity of the meta-analysis. Second, our funnel meta-regression and Cochrane Collaboration tool analysis showed significant publication bias (Additional file 1: Appendix). Third, in some scenarios, the limited number of enrolled studies, such as trials focusing on sepsis, made subgroup analysis difficult. Of note, these new biomarkers are most effective in conditions where the time of renal insult is known, for instance, post-cardiac surgery or coronary angiography, compared to situations where the onset of kidney injury is less clear, for instance, in sepsis. To ensure the robustness of the findings, we did not emphasize the diagnostic accuracy of biomarkers extracted from fewer than three articles. Fourth, we did not perform additional analyses to assess the additional predictive value of SCr levels. Most of the included studies did not measure SCr levels with biomarkers to predict AKI. In the literature, SCr has poor predictive performance for AKI due to delayed rise and cannot accurately estimate the timing of injury [118, 127]. Traditionally, the diagnosis of AKI is based on a rise in serum creatinine and the creatinine could be hard to wear two hats, having an administrative role as well as patrolling the beat. Furthermore, the use of SCr as a comparison has several limitations and limits the full interpretation of biomarker performance. For example, SCr may be elevated in pre-renal azotemia, which is not true for renal tissue damage, and biomarkers may not be elevated. On the other hand, in the setting of true renal injury with fluid overload, biomarkers may be elevated but SCr may remain unchanged, which may underestimate the predictive performance of biomarkers [149, 150]. Fifth, the kits for specific biomarker analysis varies among the studies, so it was difficult to determine the optimal cutoff value of biomarkers to predict AKI. Sixth, the occurrence of AKI was diagnosed according to several different criteria in the enrolled studies. However, the KDIGO classification was the mostly commonly used, which has been proposed to provide a uniform definition of AKI, essentially combining the RIFLE and AKIN criteria. Finally, the definition of AKI varied between the studies, and this may have unduly influenced pooled effect estimates. Nonetheless, our conclusions were drawn from studies with different study designs and different clinical scenarios. Further research efforts are certainly needed for the pursuit of better precision medicine, especially with regard to the use of multiple biomarkers. It could be more fruitful to investigate whether different etiologies of AKI (pre-renal versus renal versus obstructive, cardiogenic shock, hypovolemic shock, sepsis-related, etc.) affect the predictive accuracy of biomarkers, and to evaluate whether the efficacy of biomarkers is affected by the severity of AKI. These issues can be incorporated into the design of future randomized controlled trials to evaluate the optimal biomarkers for different clinical settings in order to improve the timely diagnosis of AKI. Moreover, further investigations to improve the diagnosis and manage the underlying mechanisms of AKI may help to mitigate the current high mortality rate of patients with AKI.

## Conclusion

Based on our pairwise meta-analysis of biomarkers to predict AKI, NGAL series had the best diagnostic accuracy for the prediction of AKI, regardless of whether or not they were adjusted by urinary creatinine, especially in medical patients. However, the predictive performance of urinary NGAL was limited in surgical patients, and NGAL/Cr seemed to be the best biomarkers in these patients. All of the biomarkers had similar predictive performance in critically ill patients. Future pragmatic clinical trials are warranted to evaluate the real-world predictive accuracy of AKI biomarkers.

## Supplementary Information

**Additional file 1: **Supplementary appendix.

## Data Availability

The datasets used and/or analyzed during the current study are available from the corresponding author on reasonable request.

## Abbreviations

AKI: Acute kidney injury
AKIN: Acute Kidney Injury Network
CKD: Chronic kidney disease
CI: Confidence interval
DOR: Diagnostic odds ratio
ESRD: End-stage renal disease
HSROC: Hierarchical summary receiver operating characteristic curve
ICU: Intensive care unit
IL-18: Interleukin-18
KDIGO: Kidney Disease: Improving Global Outcomes
KIM-1: Kidney injury molecule-1
L-FABP: Liver-type fatty acid-binding protein
NGAL: Neutrophil gelatinase-associated lipocalin
OR: Odds ratio
PRISMA: Preferred Reporting Items of Systematic Reviews and Meta-Analyses
RIFLE: Risk, injury, failure, loss, ESRD
SCr: Serum creatinine
TIMP-2 × IGFBP-7: Tissue inhibitor of metalloproteinases-2 × insulin-like growth factor-binding protein-7
