# Supplementary material for: Comparative accuracy of biomarkers for the prediction of hospital-acquired acute kidney injury: a systematic review and meta-analysis
Source: Crit Care. 2022 Nov 12;26:349. doi: 10.1186/s13054-022-04223-6 (PMC9652605; doi:10.1186/s13054-022-04223-6)
Supplement: Supplementary file 1 — Additional file 1: Supplementary appendix. [file 13054_2022_4223_MOESM1_ESM.docx]

**Supplementary appendix**

This supplementary appendix provides:

1. Search equation via PubMed, EMBASE, MEDLINE, and [Cochrane](http://www.cochranelibrary.com/) library
2. Flowchart of the updated literature search performed on Aug 15, 2022
3. Quality assessment of the included studies
4. PRISMA checklist
5. Funnel plot for publication bias
6. Supplemental tables
7. Pairwise comparison between the biomarkers
8. Summary of contextual factor data
9. PROSPERO protocol registration
10. The GRADE results
11. **Search equation via PubMed, EMBASE, MEDLINE, and** [**Cochrane**](http://www.cochranelibrary.com/) **library**

**Appendix.**

Search strategies for the different databases ran on Aug 15, 2022.

**PubMed (20988)**

("Acute Kidney Injury"[MeSH Terms] OR ("Acute Kidney Injury"[MeSH Terms] OR ("acute"[All Fields] AND "kidney"[All Fields] AND "injury"[All Fields]) OR "Acute Kidney Injury"[All Fields])) AND ("biomarker s"[All Fields] OR "biomarkers"[MeSH Terms] OR "biomarkers"[All Fields] OR "biomarker"[All Fields] OR "NGAL"[All Fields] OR "NGAL"[All Fields] OR ("lipocalin 2"[MeSH Terms] OR "lipocalin 2"[All Fields] OR ("neutrophil"[All Fields] AND "gelatinase"[All Fields] AND "associated"[All Fields] AND "lipocalin"[All Fields]) OR "Neutrophil Gelatinase Associated Lipocalin"[All Fields]) OR "Neutrophil Gelatinase Associated Lipocalin"[All Fields] OR ("interleukin 18"[MeSH Terms] OR "interleukin 18"[All Fields] OR "il 18"[All Fields]) OR ("interleukin 18"[MeSH Terms] OR "interleukin 18"[All Fields] OR "interleukin 18"[All Fields]) OR ("creatinin"[All Fields] OR "creatinine"[MeSH Terms] OR "creatinine"[All Fields] OR "creatinines"[All Fields]) OR "L-FABP"[All Fields] OR ("Liver-type"[All Fields] AND ("fatty acid binding proteins"[MeSH Terms] OR ("fatty"[All Fields] AND "acid binding"[All Fields] AND "proteins"[All Fields]) OR "fatty acid binding proteins"[All Fields] OR ("fatty"[All Fields] AND "acid"[All Fields] AND "binding"[All Fields] AND "protein"[All Fields]) OR "fatty acid binding protein"[All Fields] OR "aspartate aminotransferase, mitochondrial"[MeSH Terms] OR ("aspartate"[All Fields] AND "aminotransferase"[All Fields] AND "mitochondrial"[All Fields]) OR "mitochondrial aspartate aminotransferase"[All Fields] OR ("fatty"[All Fields] AND "acid"[All Fields] AND "binding"[All Fields] AND "protein"[All Fields]))) OR "KIM-1"[All Fields] OR (("kidney"[MeSH Terms] OR "kidney"[All Fields] OR "kidneys"[All Fields] OR "kidney s"[All Fields]) AND ("injurie"[All Fields] OR "injuried"[All Fields] OR "injuries"[MeSH Subheading] OR "injuries"[All Fields] OR "wounds and injuries"[MeSH Terms] OR ("wounds"[All Fields] AND "injuries"[All Fields]) OR "wounds and injuries"[All Fields] OR "injurious"[All Fields] OR "injury s"[All Fields] OR "injuryed"[All Fields] OR "injurys"[All Fields] OR "injury"[All Fields]) AND ("molecule"[All Fields] OR "molecule s"[All Fields] OR "molecules"[All Fields]) AND "1"[All Fields]) OR (("kidney"[MeSH Terms] OR "kidney"[All Fields] OR "kidneys"[All Fields] OR "kidney s"[All Fields]) AND ("molecule"[All Fields] OR "molecule s"[All Fields] OR "molecules"[All Fields]) AND "1"[All Fields]) OR "TIM-1"[All Fields] OR (("t lymphocytes"[MeSH Terms] OR "t lymphocytes"[All Fields] OR "t cell"[All Fields]) AND ("immunoglobulin s"[All Fields] OR "immunoglobuline"[All Fields] OR "immunoglobulines"[All Fields] OR "immunoglobulins"[MeSH Terms] OR "immunoglobulins"[All Fields] OR "immunoglobulin"[All Fields]) AND ("mucine"[All Fields] OR "mucines"[All Fields] OR "mucinous"[All Fields] OR "mucins"[MeSH Terms] OR "mucins"[All Fields] OR "mucin"[All Fields]) AND ("domain s"[All Fields] OR "domains"[All Fields] OR "protein domains"[MeSH Terms] OR ("protein"[All Fields] AND "domains"[All Fields]) OR "protein domains"[All Fields] OR "domain"[All Fields]) AND "1"[All Fields]) OR (("tissue inhibitor of metalloproteinase 2"[MeSH Terms] OR ("tissue"[All Fields] AND "inhibitor"[All Fields] AND "metalloproteinase 2"[All Fields]) OR "tissue inhibitor of metalloproteinase 2"[All Fields] OR "timp 2"[All Fields]) AND "x"[All Fields] AND ("insulin like growth factor binding protein related protein 1"[Supplementary Concept] OR "insulin like growth factor binding protein related protein 1"[All Fields] OR "igfbp 7"[All Fields])) OR (("TIMP2"[All Fields] AND "x"[All Fields]) AND "IGFBP7"[All Fields]) OR "IGFBP7"[All Fields] OR ("tissue inhibitor of metalloproteinase 2"[MeSH Terms] OR ("tissue"[All Fields] AND "inhibitor"[All Fields] AND "metalloproteinase 2"[All Fields]) OR "tissue inhibitor of metalloproteinase 2"[All Fields] OR "timp 2"[All Fields]) OR "Nephrocheck"[All Fields] OR "tissue inhibitor of metalloproteinases-2"[All Fields] OR "insulin-like growth factor-binding protein 7"[All Fields] OR "PENK"[All Fields] OR ("proenkephalin"[Supplementary Concept] OR "proenkephalin"[All Fields] OR "proenkephalins"[All Fields]))

**EMBASE(1213)**

(acute AND kidney AND injury AND biomarker OR biomarkers OR ngal OR (neutrophil AND gelatinase AND associated AND lipocalin) OR 'il 18' OR 'interleukin 18' OR creatinine OR 'l fabp' OR ('liver type' AND fatty AND acid AND binding AND protein) OR 'kim 1' OR (kidney AND injury AND molecule AND 1) OR (kidney AND molecule AND 1) OR 'tim 1' OR (t AND cell AND immunoglobulin AND mucin AND domain AND 1) OR igfbp7 OR timp2 OR nephrocheck OR penk OR proenkephalin OR (tissue AND inhibitor AND of AND 'metalloproteinases 2')) AND 'insulin like' AND growth AND 'factor binding' AND protein AND 7

**Medline (223)**

((("Acute Kidney Injury"[Mesh]) OR Acute kidney injury)AND(Biomarker OR Biomarkers OR (NGAL) OR (Neutrophil Gelatinase Associated Lipocalin) OR (IL-18) OR (interleukin-18) OR (Creatinine) OR (L-FABP) OR (Liver-type fatty acid binding protein) OR (KIM-1) OR (Kidney injury molecule 1) OR (TIM-1) OR (T cell immunoglobulin mucin domain 1) OR (TIMP-2 x IGFBP-7) OR (tissue inhibitor metalloproteinases-2) OR (insulin-like growth factor-binding protein 7) OR (PENK) OR (Proenkephalin) OR((TIMP2)x(IGFBP7)) OR TIMP2 OR IGFBP7)) {Including Related Terms}

**Cochrane library (2219)**

#1 MeSH descriptor: [Acute Kidney Injury] explode all trees

#2 "Acute kidney injury"

#3 #1 OR #2

#4 Biomarker

#5 NGAL

#6 "Neutrophil Gelatinase Associated Lipocalin"

#7 IL-18

#8 "interleukin-18"

#9 Creatinine

#10 L-FABP

#11 "Liver-type fatty acid binding protein"

#12 KIM-1

#13 "Kidney injury molecule 1"

#14 "Kidney molecule 1"

#15 TIM-1

#16 "T cell immunoglobulin mucin domain 1"

#17 TIMP-2 x IGFBP-7

#18 IGFBP7

#19 TIMP-2

#20 Nephrocheck

#21 "tissue inhibitor of metalloproteinases-2"

#22 "insulin-like growth factor-binding protein 7"

#23 PENK

#24 Proenkephalin

#25 #4 OR #5 OR #6 OR #7 OR #8 OR #9 OR #10 OR #11 OR #12 OR #13 OR #14 OR #15 OR #16 OR #17 OR #18 OR #19 OR #20 OR #21 OR #22 OR #23 OR #24

#26 #3 AND #25

1. **Flowchart of study selection for meta-analysis.**


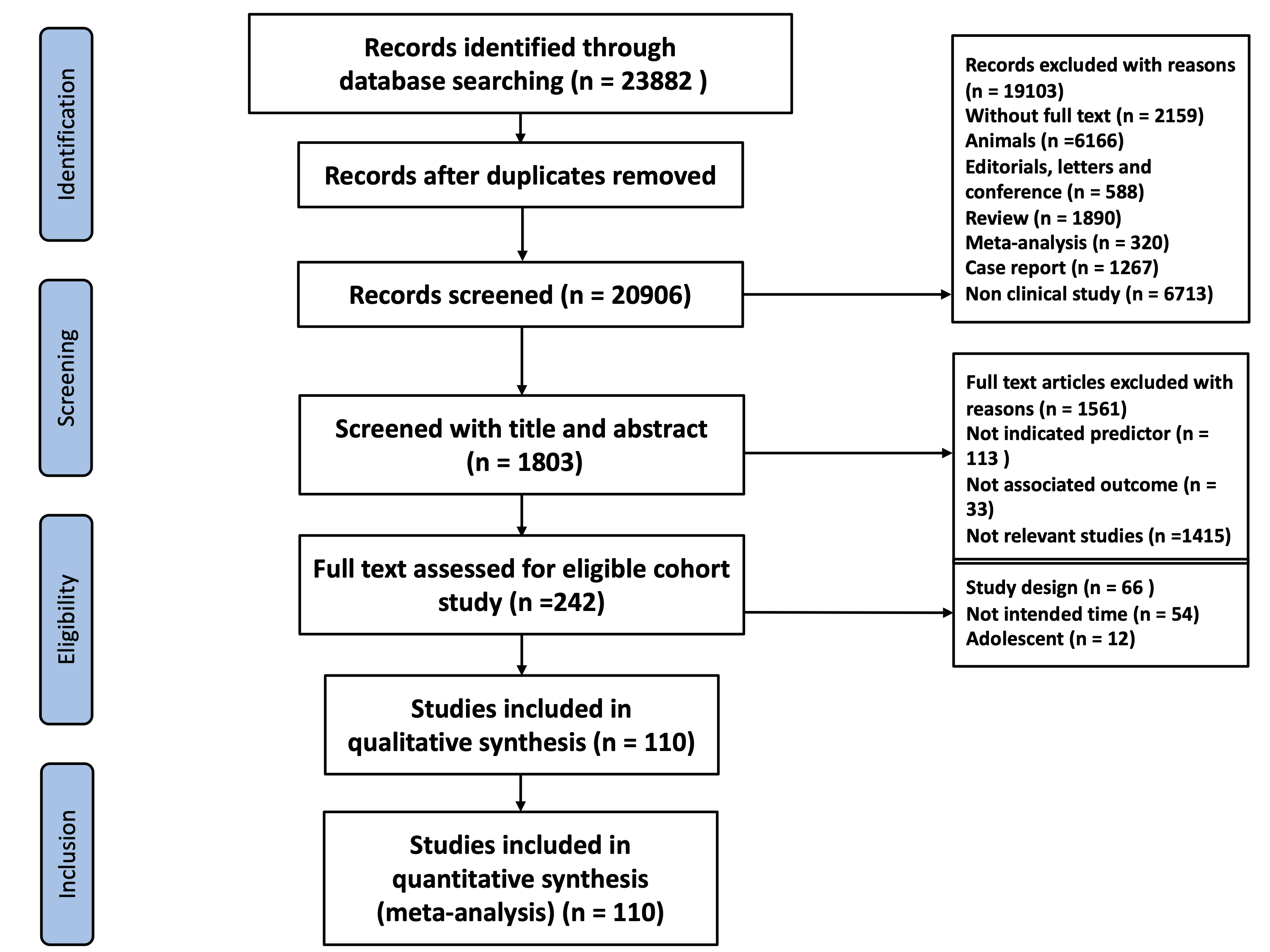


1. **Quality assessment of the included studies**

**Supplemental Figure 1. Overview about the risk of bias and applicability concerns graph: using the QUADAS-2 tool based on the complete literature-based analysis**


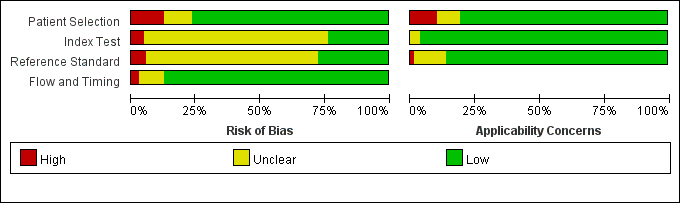


**Supplemental Figure 2**: **Risk of bias and applicability concerns using the QUADAS-2 tool for each included study based on the complete literature-based analysis**


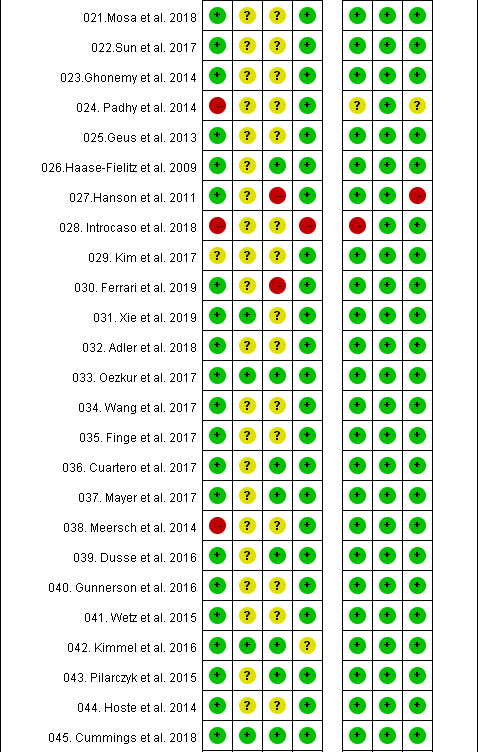


　
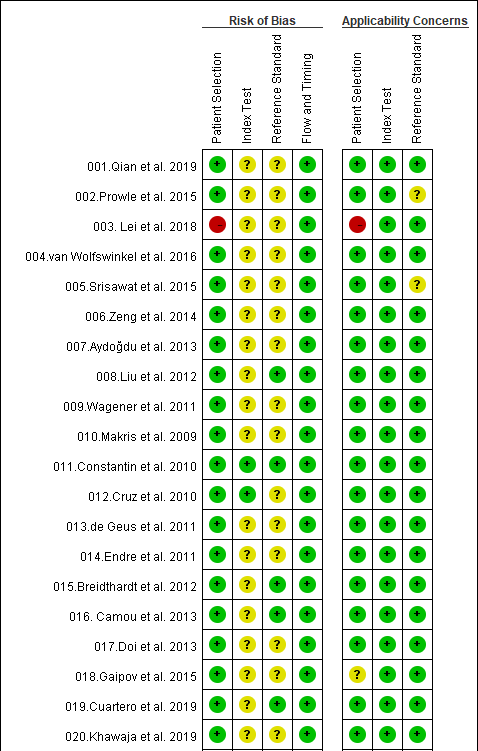


　
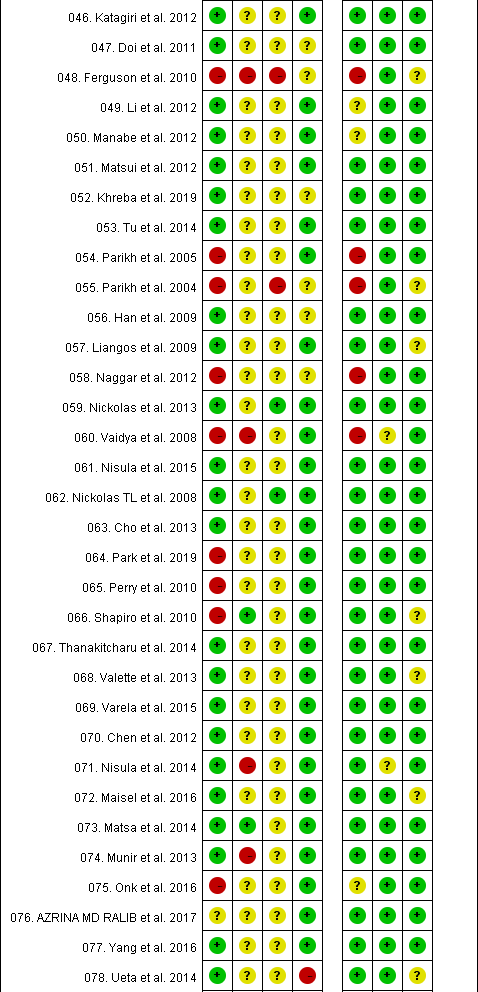


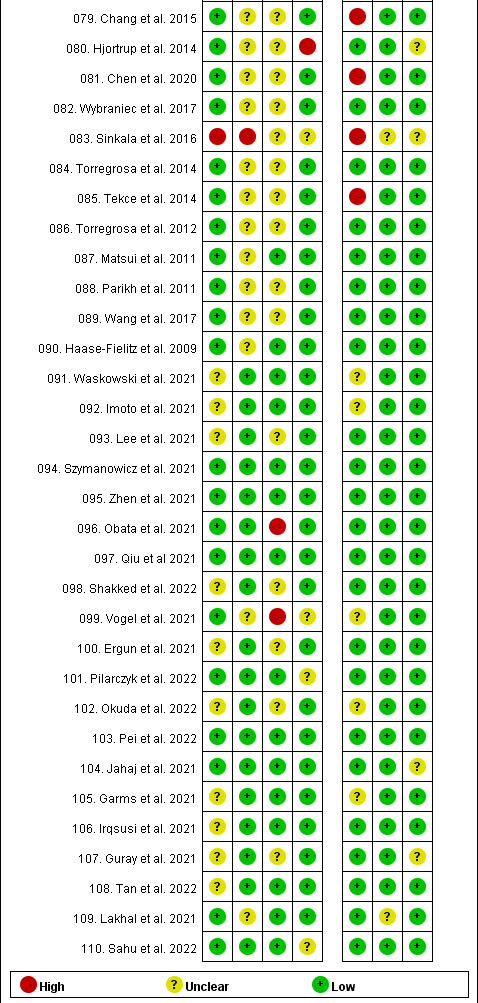


1. **PRISMA checklist**

**5. Funnel plot for publication bias**. There were apparent asymmtery pattern for the funnel plots IL-18, KIM-1, KIM-1/Cr, L-FABP, urinary NGAL, urinary NGAL/Cr, and serum NGAL.


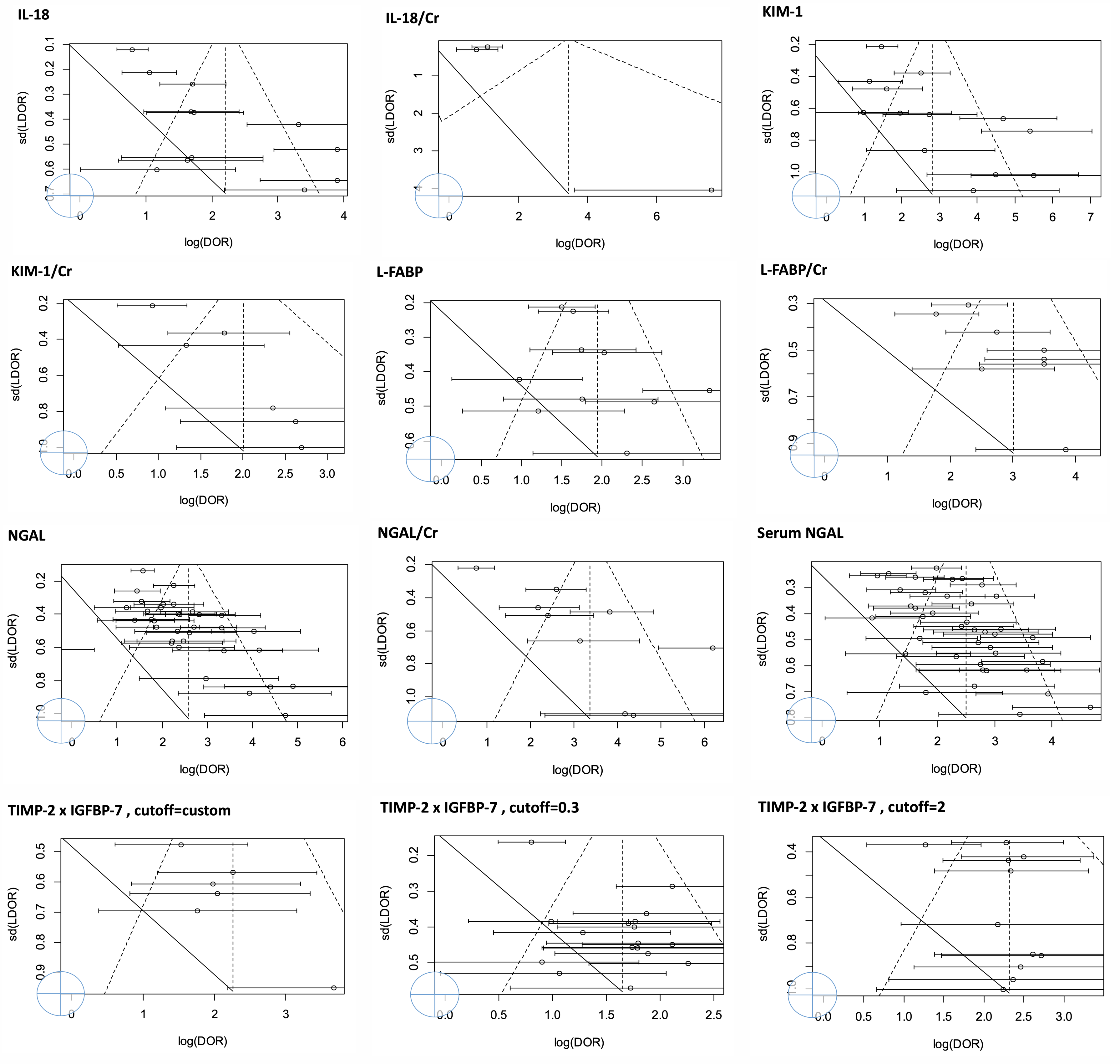


**Abbreviations:** Cr, creatinine; IL-18, interleukin-18; KIM-1, kidney injury molecule-1; L-FABP, liver-type fatty acid binding protein; NGAL, neutrophil gelatinase-associated lipocalin; TIMP-2 x IGFBP-7: tissue inhibitor of metalloproteinases-2 x insulin-like growth factor-binding protein 7.

**6. Supplemental tables**

**Supplemental Table 1. Summary of the diagnostic meta-analysis in the non-sepsis population**

**Abbreviations:** CI, confidence interval; DOR, diagnostic odds ratio; NGAL, neutrophil gelatinase associated lipocalin; IL-18, interleukin-18; Cr, urine creatinine; KIM-1, kidney injury molecule-1; L-FABP, liver-type fatty acid binding protein; TIMP-2 x IGFBP-7, tissue inhibitor of metalloproteinases-2 x insulin-like growth factor binding protein-7;

“*” Indicates significant difference (*P* <0.05) versus the referent category: “NGAL”.

**Supplemental Table 2. Summary of the diagnostic meta-analysis in the high and low/moderate quality studies.**

Abbreviations: CI, confidence interval; DOR, diagnostic odds ratio; NGAL, neutrophil gelatinase associated lipocalin; IL-18, interleukin-18; Cr, urine creatinine; KIM-1, kidney injury molecule-1; L-FABP, liver-type fatty acid binding protein; TIMP-2 x IGFBP-7, tissue inhibitor of metalloproteinases-2 x insulin-like growth factor binding protein-7;

“*” Indicates significant difference (*P* <0.05) versus the referent category: “NGAL”.

**Supplemental Table 3. Summary of the diagnostic meta-analysis in the studies conducted in high and low/middle income countries.**

Abbreviations: CI, confidence interval; DOR, diagnostic odds ratio; NGAL, neutrophil gelatinase associated lipocalin; IL-18, interleukin-18; Cr, urine creatinine; KIM-1, kidney injury molecule-1; L-FABP, liver-type fatty acid binding protein; TIMP-2 x IGFBP-7, tissue inhibitor of metalloproteinases-2 x insulin-like growth factor binding protein-7;

“*” Indicates significant difference (*P* <0.05) versus the referent category: “NGAL”.

**Supplemental Table 4. Summary of the diagnostic meta-analysis in the early AKI population.**

Abbreviations: CI, confidence interval; DOR, diagnostic odds ratio; NGAL, neutrophil gelatinase associated lipocalin; IL-18, interleukin-18; Cr, urine creatinine; KIM-1, kidney injury molecule-1; L-FABP, liver-type fatty acid binding protein; TIMP-2 x IGFBP-7, tissue inhibitor of metalloproteinases-2 x insulin-like growth factor binding protein-7;

“*” Indicates significant difference (*P* <0.05) versus the referent category: “NGAL”.

**Supplemental Table 5. Summary of the diagnostic meta-analysis in the severe AKI population.**

Abbreviations: CI, confidence interval; DOR, diagnostic odds ratio; NGAL, neutrophil gelatinase associated lipocalin; IL-18, interleukin-18; Cr, urine creatinine; KIM-1, kidney injury molecule-1; L-FABP, liver-type fatty acid binding protein; TIMP-2 x IGFBP-7, tissue inhibitor of metalloproteinases-2 x insulin-like growth factor binding protein-7;

“*” Indicates significant difference (*P* <0.05) versus the referent category: “NGAL”.

**Supplemental Table 6. Summary of the diagnostic meta-analysis in the RRT population.**

Abbreviations: CI, confidence interval; DOR, diagnostic odds ratio; NGAL, neutrophil gelatinase associated lipocalin; IL-18, interleukin-18; Cr, urine creatinine; KIM-1, kidney injury molecule-1; L-FABP, liver-type fatty acid binding protein; TIMP-2 x IGFBP-7, tissue inhibitor of metalloproteinases-2 x insulin-like growth factor binding protein-7;

“*” Indicates significant difference (*P* <0.05) versus the referent category: “NGAL”.

**7. Pairwise comparison between the biomarkers**

**Supplemental Figure 3.** **Heatmap plot depicted pairwise comparison (row vs. column) of relative sensitivity between the biomarkers in the whole population.** The contents of the diagonal are the values of the relative sensitivity. Red depicts positive relative sensitivity while yellow depicts no correlation. L-FABP/Cr and TIMP-2 x IGFBP-7: custom had the best relative sensitivity in the biomarkers.


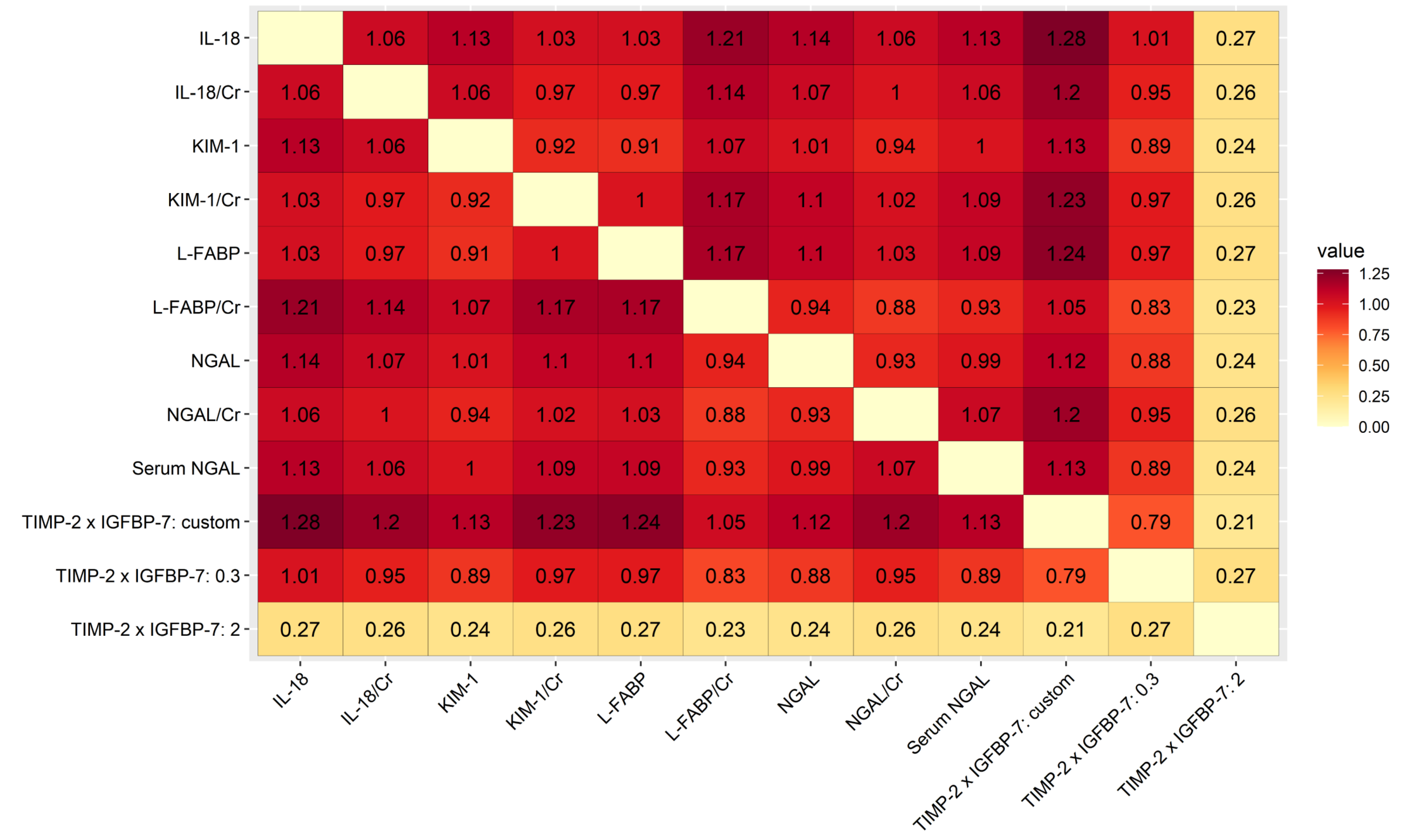


**Abbreviations:** Cr, creatinine; IL-18, interleukin-18; KIM-1, kidney injury molecule-1; L-FABP, liver-type fatty acid binding protein; NGAL, neutrophil gelatinase-associated lipocalin; TIMP-2 x IGFBP-7: tissue inhibitor of metalloproteinases-2 x insulin-like growth factor-binding protein 7.

**Supplemental Figure 4. Heatmap plot depicted pairwise comparison (row vs. column) of relative specificity between the biomarkers in the whole population.** The contents of the diagonal are the values of the relative specificity. Red depicts positive relative specificity while yellow depicts no correlation. NGAL/Cr and TIMP-2 x IGFBP-7: 2 had the best relative specificity in the biomarkers.


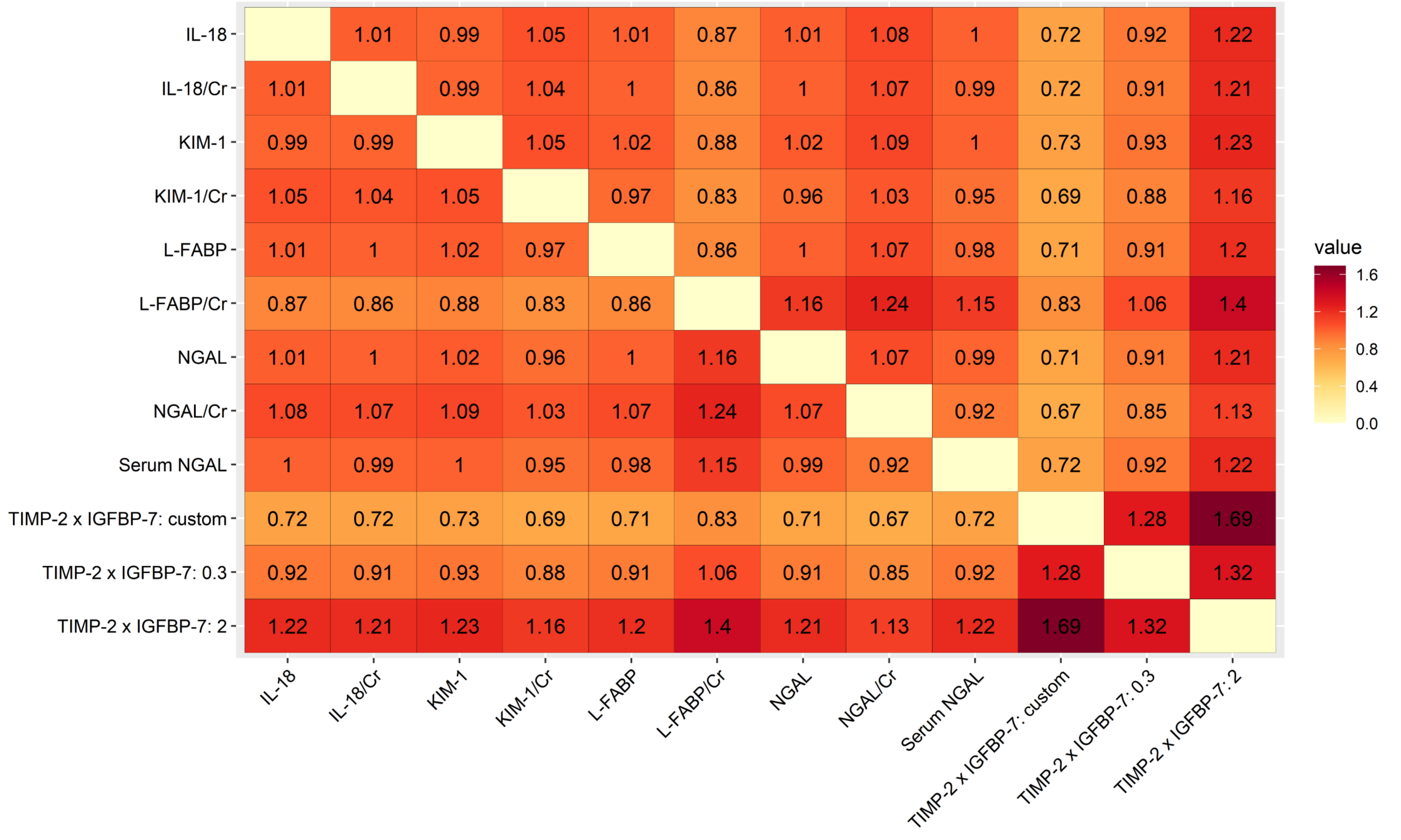


**Abbreviations:** Cr, creatinine; IL-18, interleukin-18; KIM-1, kidney injury molecule-1; L-FABP, liver-type fatty acid binding protein; NGAL, neutrophil gelatinase-associated lipocalin; TIMP-2 x IGFBP-7: tissue inhibitor of metalloproteinases-2 x insulin-like growth factor-binding protein 7.

**Supplemental Figure 5.** **Heatmap plot depicted pairwise comparison (row vs. column) of relative sensitivity between the biomarkers in the ICU subgroup.** The contents of the diagonal are the values of the relative sensitivity. Red depicts positive sensitivity while yellow depicts no correlation. L-FABP/Cr and TIMP-2 x IGFBP-7: custom had the best relative sensitivity in the biomarkers.


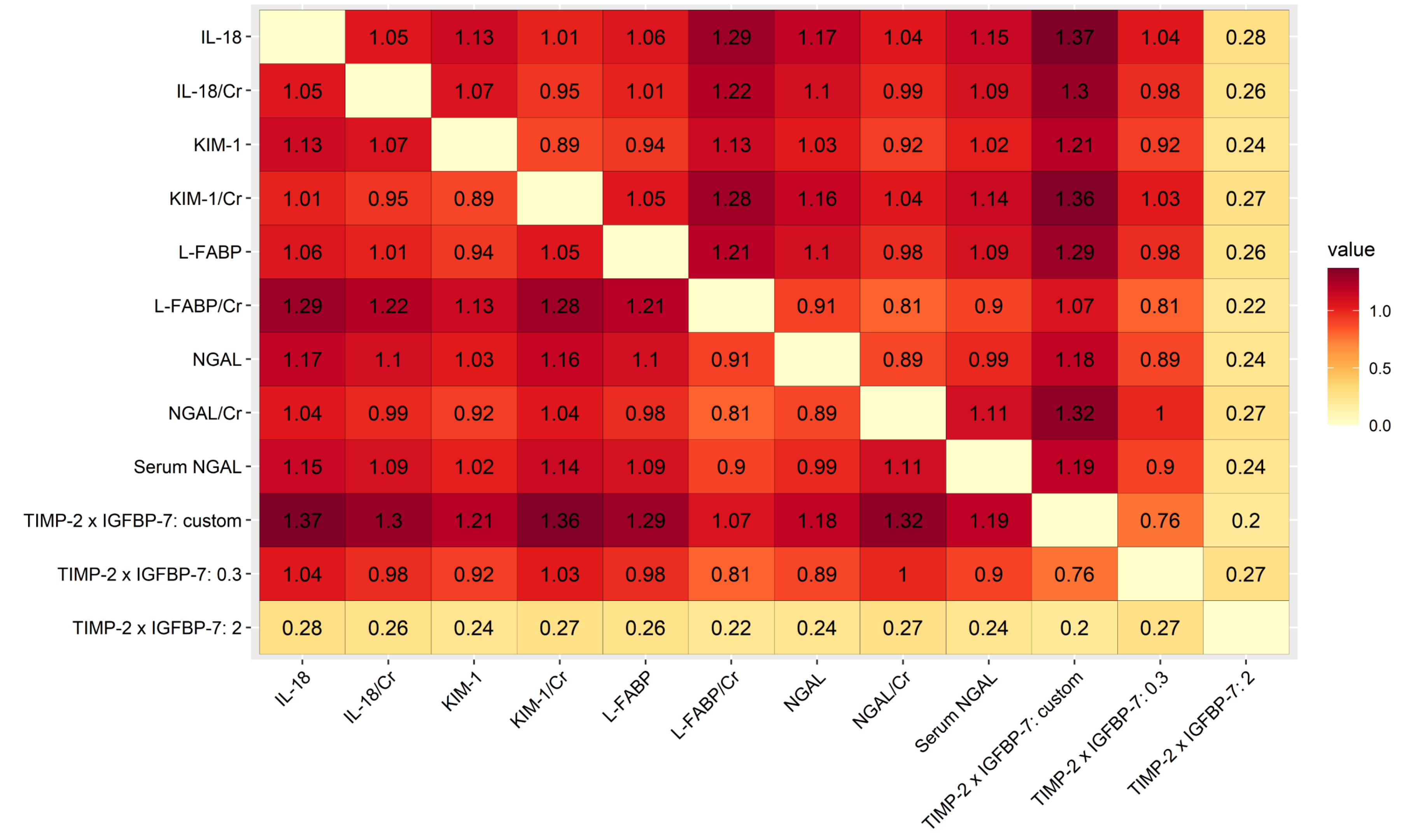


**Abbreviations:** Cr, creatinine; IL-18, interleukin-18; KIM-1, kidney injury molecule-1; L-FABP, liver-type fatty acid binding protein; NGAL, neutrophil gelatinase-associated lipocalin; TIMP-2 x IGFBP-7: tissue inhibitor of metalloproteinases-2 x insulin-like growth factor-binding protein 7.

**Supplemental Figure 6.** **Heatmap plot depicted pairwise comparison (row vs. column) of relative specificity between the biomarkers in the ICU subgroup.** The contents of the diagonal are the values of the relative specificity. Red depicts positive specificity while yellow depicts no correlation. TIMP-2 x IGFBP-7: 2 had the best relative specificity in the biomarkers.


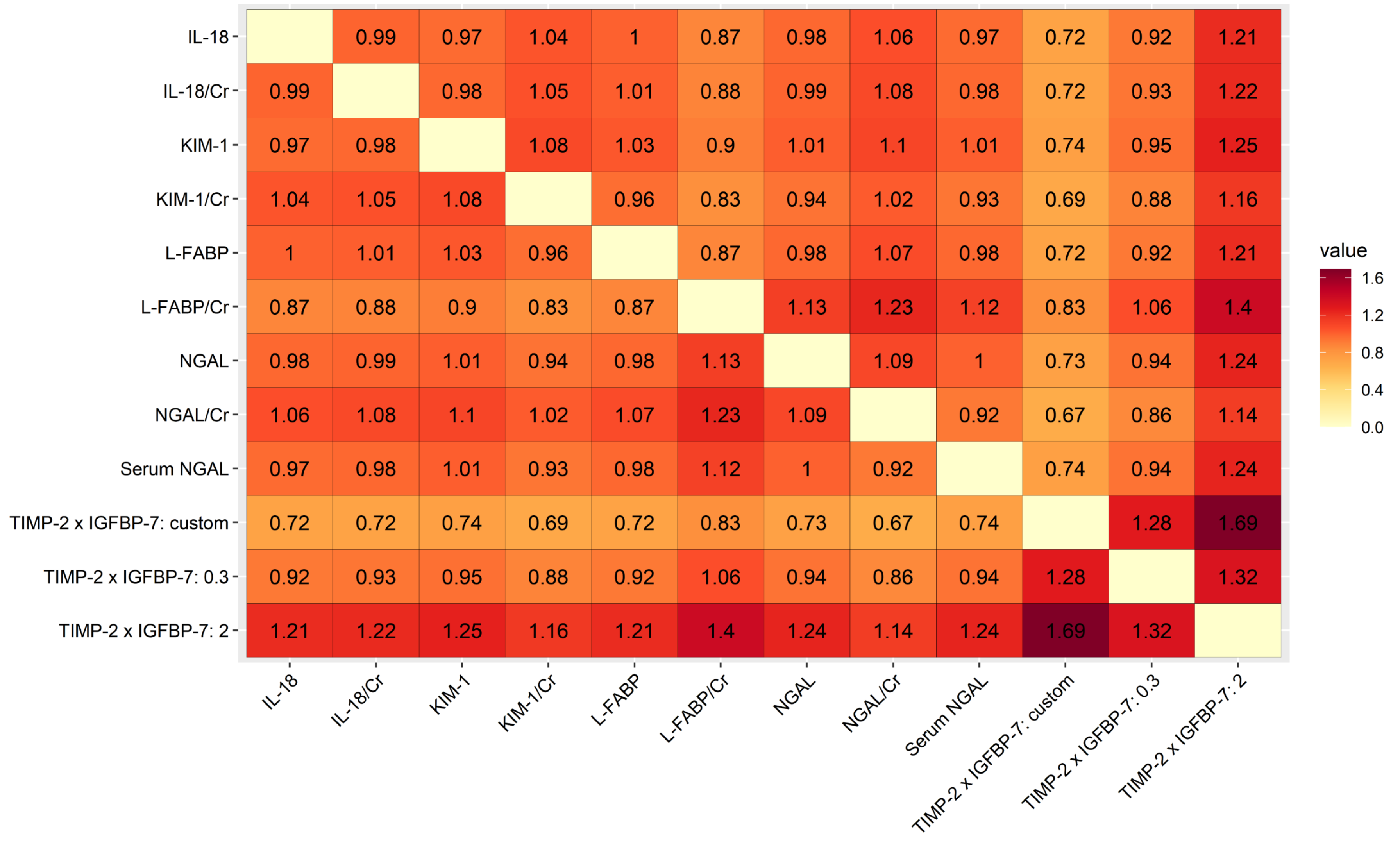


**Abbreviations:** Cr, creatinine; IL-18, interleukin-18; KIM-1, kidney injury molecule-1; L-FABP, liver-type fatty acid binding protein; NGAL, neutrophil gelatinase-associated lipocalin; TIMP-2 x IGFBP-7: tissue inhibitor of metalloproteinases-2 x insulin-like growth factor-binding protein 7.

**Supplemental Figure 7.** **Heatmap plot depicted pairwise comparison (row vs. column) of relative DOR between the biomarkers in the ICU subgroup.** The contents of the diagonal are the values of the relative DOR. Red depicts positive DOR while yellow depicts no correlation. L-FABP/Cr had the best relative DOR in the biomarkers.


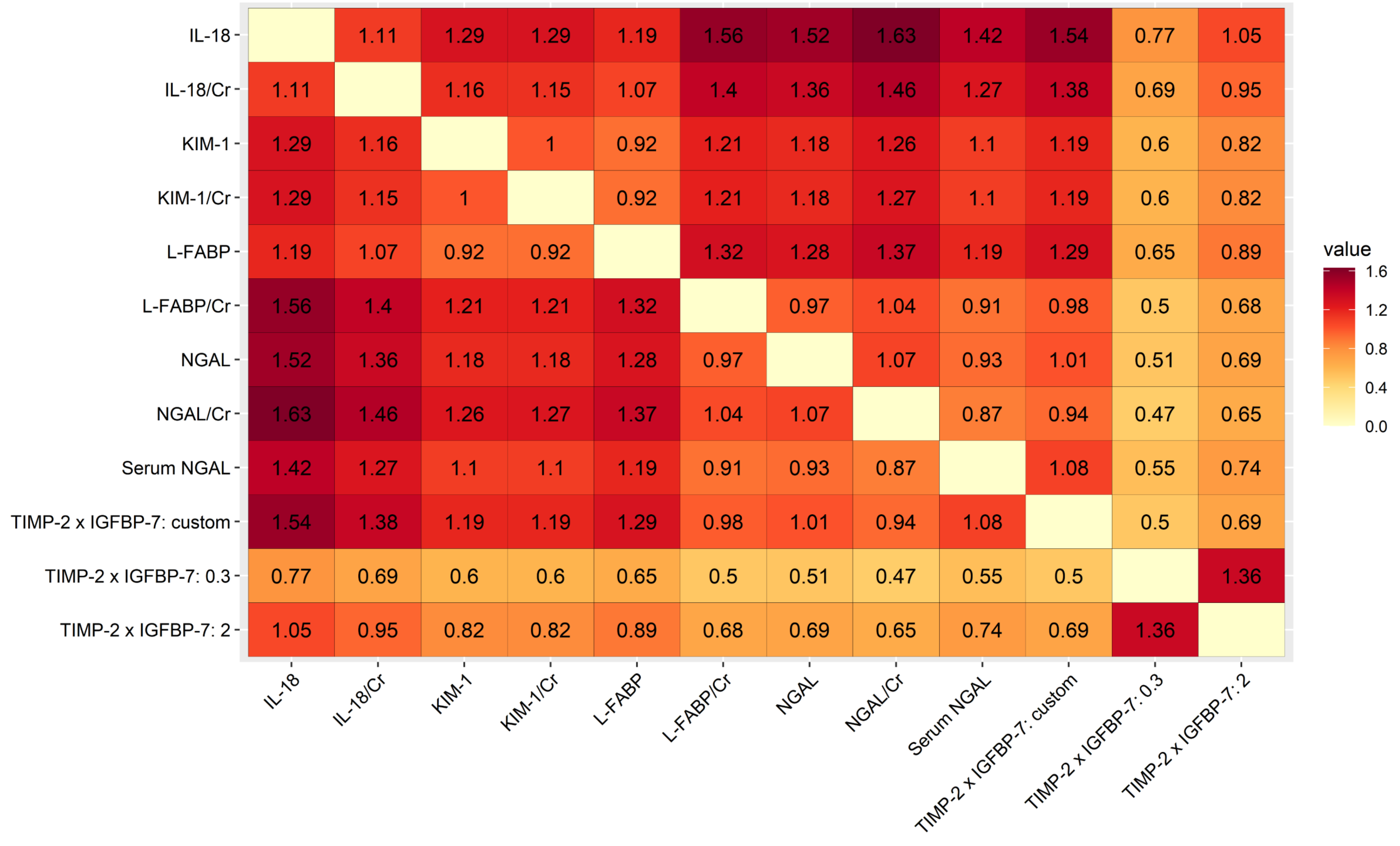


**Abbreviations:** Cr, creatinine; DOR, diagnostic odds-ratio; IL-18, interleukin-18; KIM-1, kidney injury molecule-1; L-FABP, liver-type fatty acid binding protein; NGAL, neutrophil gelatinase-associated lipocalin; TIMP-2 x IGFBP-7: tissue inhibitor of metalloproteinases-2 x insulin-like growth factor-binding protein 7.

**Supplemental Figure 8.** **Heatmap plot depicted pairwise comparison (row vs. column) of relative sensitivity between the biomarkers in the medical subgroup.** The contents of the diagonal are the values of the relative sensitivity. Red depicts positive sensitivity while yellow depicts no correlation. L-FABP/Cr and NGAL had the best relative sensitivity in the biomarkers.


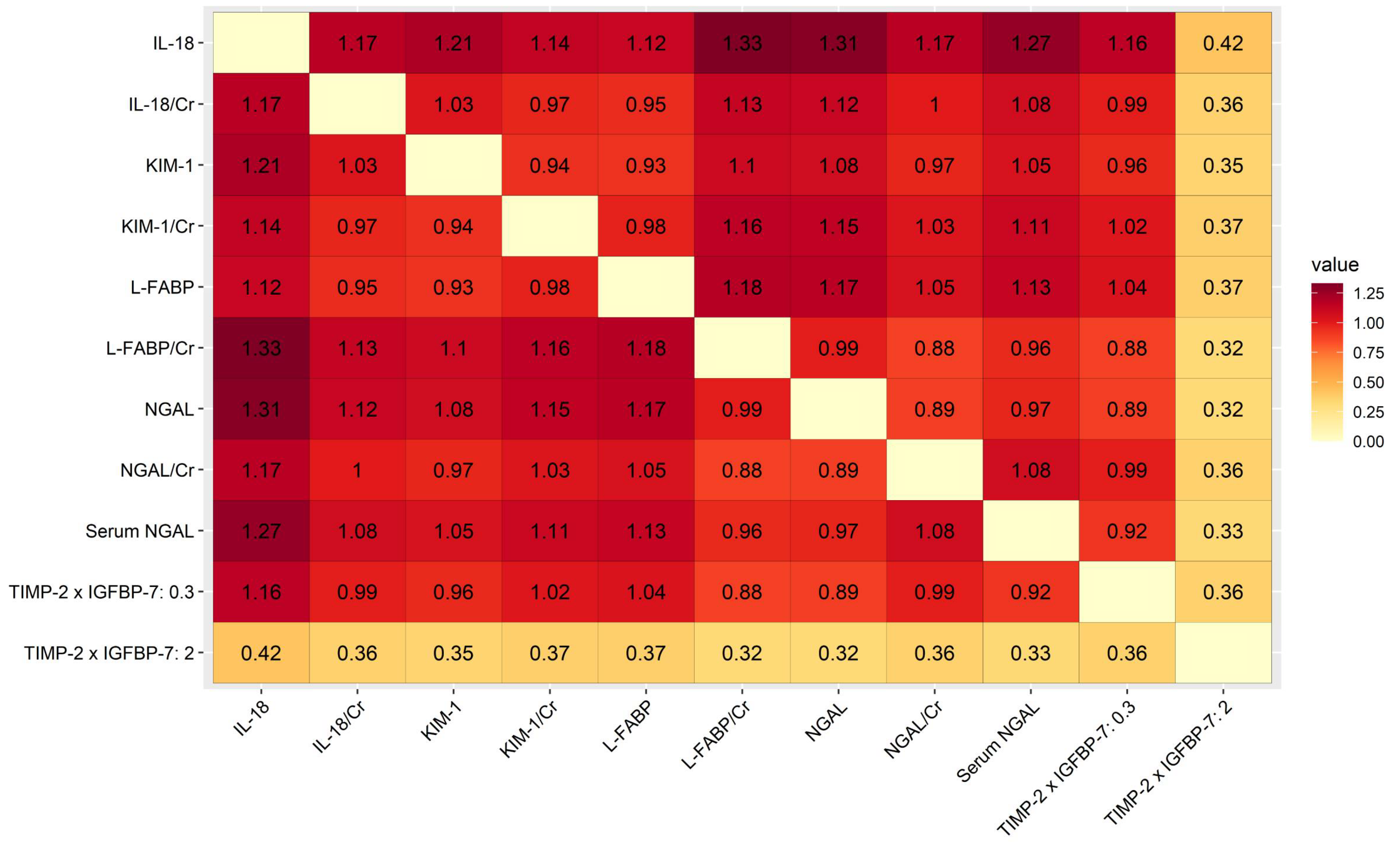


**Abbreviations:** Cr, creatinine; IL-18, interleukin-18; KIM-1, kidney injury molecule-1; L-FABP, liver-type fatty acid binding protein; NGAL, neutrophil gelatinase-associated lipocalin; TIMP-2 x IGFBP-7: tissue inhibitor of metalloproteinases-2 x insulin-like growth factor-binding protein 7.

**Supplemental Figure 9. Heatmap plot depicted pairwise comparison (row vs. column) of relative specificity between the biomarkers in the medical subgroup.** The contents of the diagonal are the values of the relative specificity. Red depicts positive specificity while yellow depicts no correlation. TIMP-2 x IGFBP-7: 2 had the best relative specificity in the biomarkers.


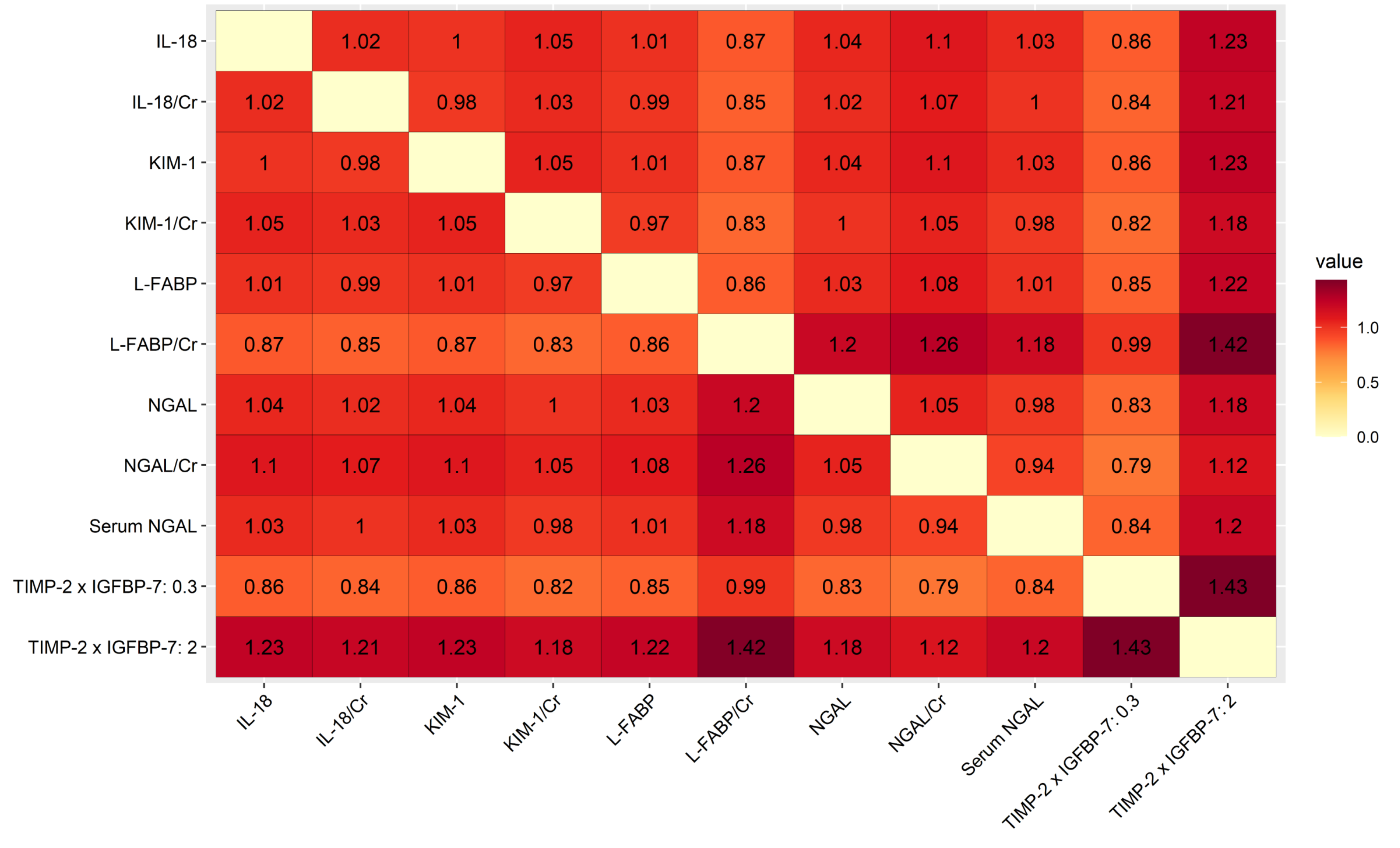


**Abbreviations:** Cr, creatinine; IL-18, interleukin-18; KIM-1, kidney injury molecule-1; L-FABP, liver-type fatty acid binding protein; NGAL, neutrophil gelatinase-associated lipocalin; TIMP-2 x IGFBP-7: tissue inhibitor of metalloproteinases-2 x insulin-like growth factor-binding protein 7.

**Supplemental Figure 10.** **Heatmap plot depicted** **pairwise comparison (row vs. column) of relative DOR between the biomarkers in the medical subgroup.** The contents of the diagonal are the values of the relative DOR. Red depicts positive DOR while yellow depicts no correlation. NGAL serials had the best relative DOR in the biomarkers.


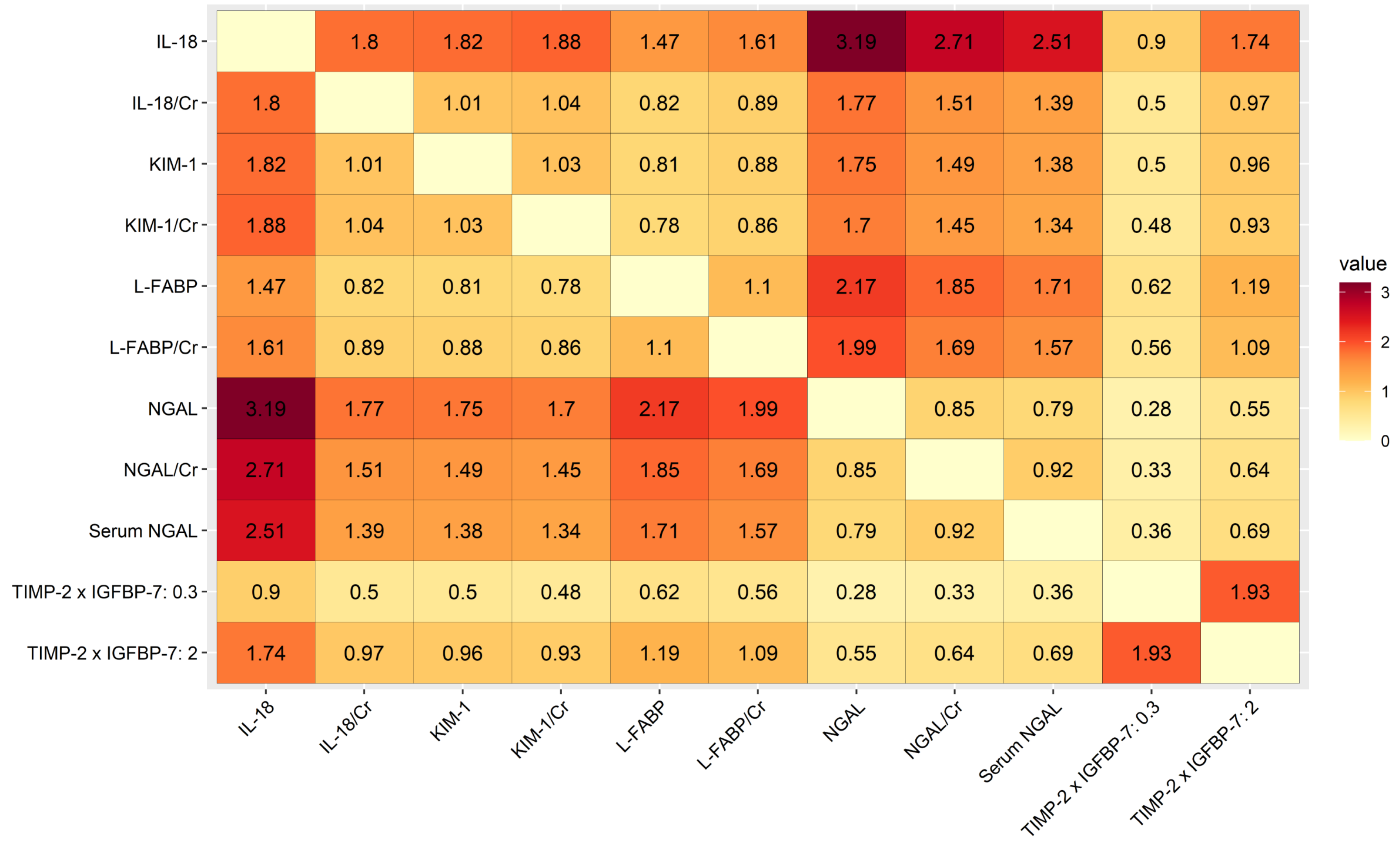


**Abbreviations:** Cr, creatinine; DOR, diagnostic odds-ratio; IL-18, interleukin-18; KIM-1, kidney injury molecule-1; L-FABP, liver-type fatty acid binding protein; NGAL, neutrophil gelatinase-associated lipocalin; TIMP-2 x IGFBP-7: tissue inhibitor of metalloproteinases-2 x insulin-like growth factor-binding protein 7.

**Supplemental Figure 11. Heatmap plot depicted pairwise comparison (row vs. column) of relative sensitivity between the biomarkers in the surgical subgroup.** The contents of the diagonal are the values of the relative sensitivity. Red depicts positive relative sensitivity while yellow depicts no correlation. NGAL/Cr and TIMP-2 x IGFBP-7: custom had the best relative sensitivity in the biomarkers.


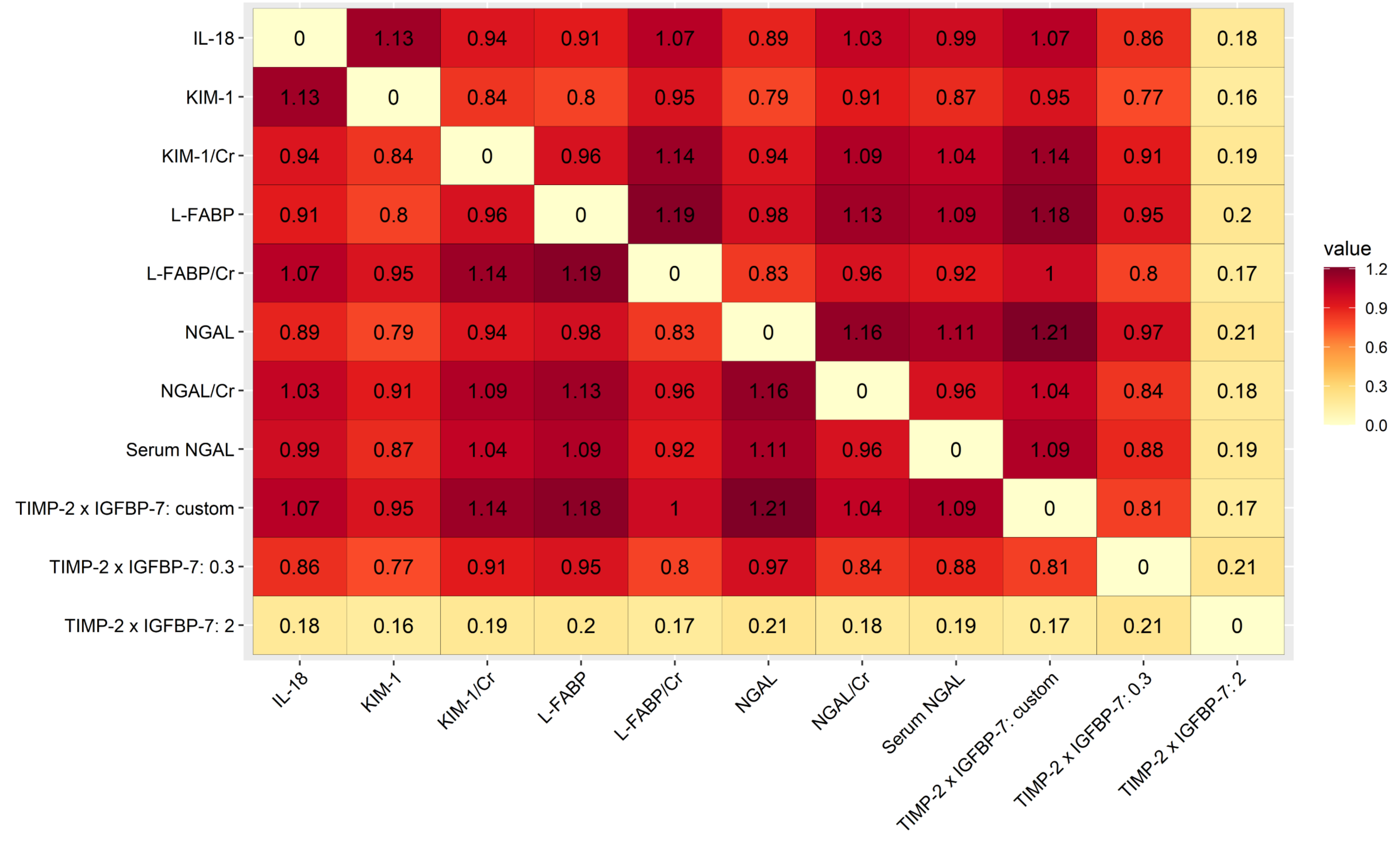


**Abbreviations:** Cr, creatinine; interleukin-18; KIM-1, kidney injury molecule-1; L-FABP, liver-type fatty acid binding protein; NGAL, neutrophil gelatinase-associated lipocalin; TIMP-2 x IGFBP-7: tissue inhibitor of metalloproteinases-2 x insulin-like growth factor-binding protein 7.

**Supplemental Figure 12.** Heatmap plot depicted **pairwise comparison (row vs. column) of relative specificity between the biomarkers in the surgical subgroup.** The contents of the diagonal are the values of the relative specificity. Red depicts positive specificity while yellow depicts no correlation. NGAL/Cr and TIMP-2 x IGFBP-7: 2 had the best relative specificity in the biomarkers.


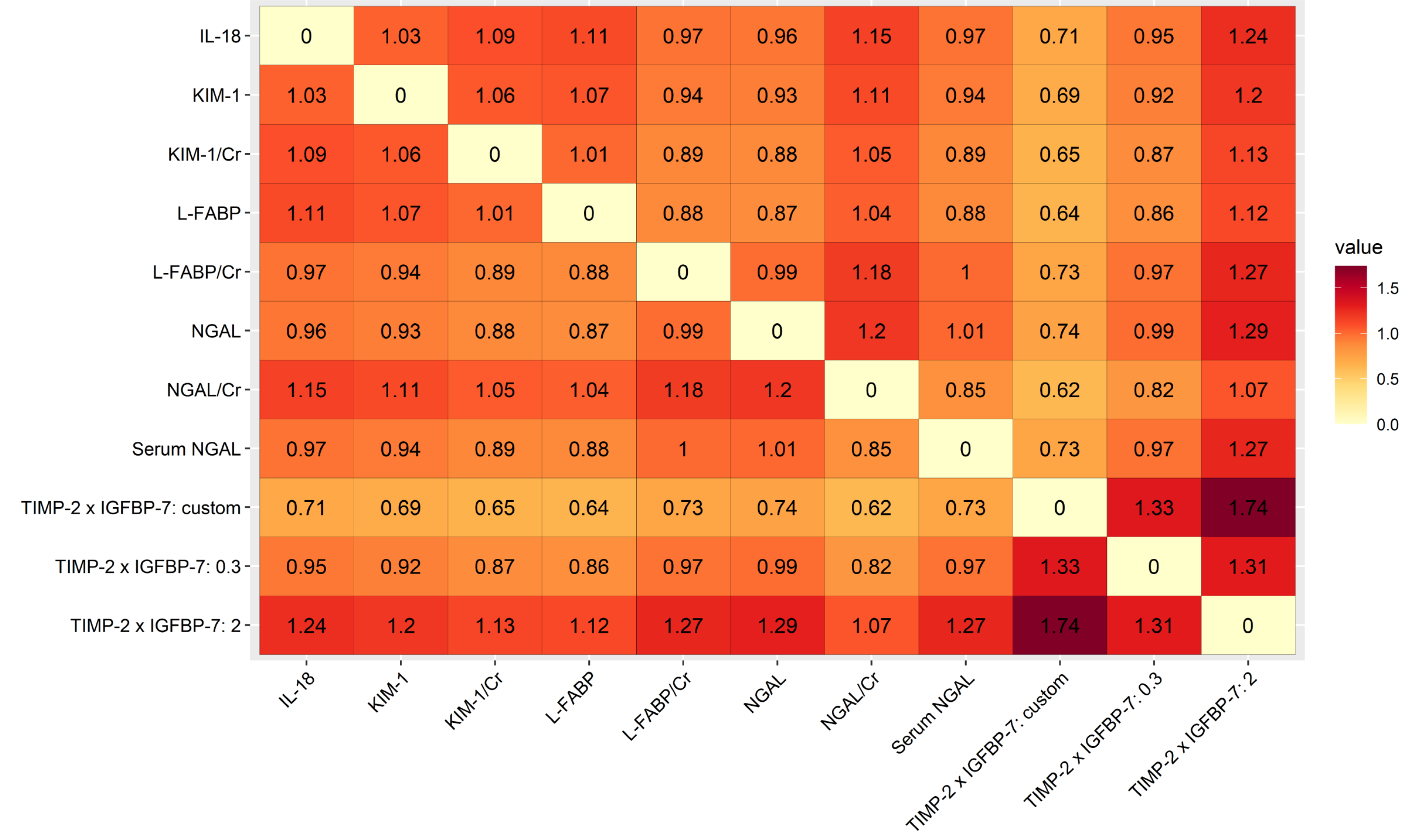


**Abbreviations:** Cr, creatinine; IL-18, interleukin-18; KIM-1, kidney injury molecule-1; L-FABP, liver-type fatty acid binding protein; NGAL, neutrophil gelatinase-associated lipocalin; TIMP-2 x IGFBP-7: tissue inhibitor of metalloproteinases-2 x insulin-like growth factor-binding protein 7.

**Supplemental Figure 13.** **Heatmap plot depicted pairwise comparison (row vs. column) of relative sensitivity between the biomarkers in the non-sepsis subgroup.** The contents of the diagonal are the values of the relative sensitivity. Red depicts positive sensitivity while yellow depicts no correlation. TIMP-2 x IGFBP-7: custom had the best relative sensitivity in the biomarkers.


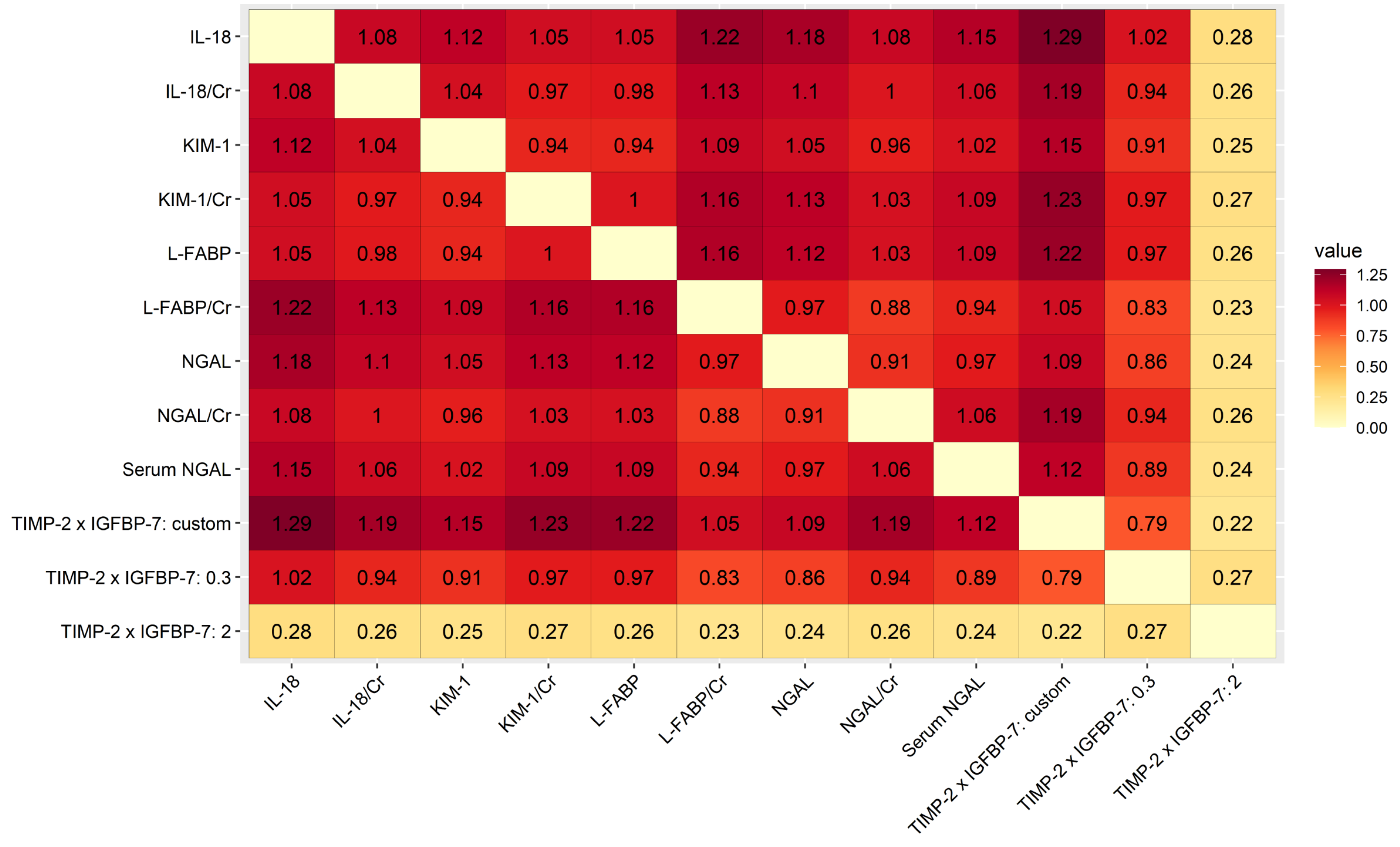


**Abbreviations:** Cr, creatinine; IL-18, interleukin-18; KIM-1, kidney injury molecule-1; L-FABP, liver-type fatty acid binding protein; NGAL, neutrophil gelatinase-associated lipocalin; TIMP-2 x IGFBP-7: tissue inhibitor of metalloproteinases-2 x insulin-like growth factor-binding protein 7.

**Supplemental Figure 14.** **Heatmap plot depicted pairwise comparison (row vs. column) of relative specificity between the biomarkers in the non-sepsis subgroup.** The contents of the diagonal are the values of the relative specificity. Red depicts positive specificity while yellow depicts no correlation. TIMP-2 x IGFBP-7: 2 had the best relative specificity in the biomarkers.


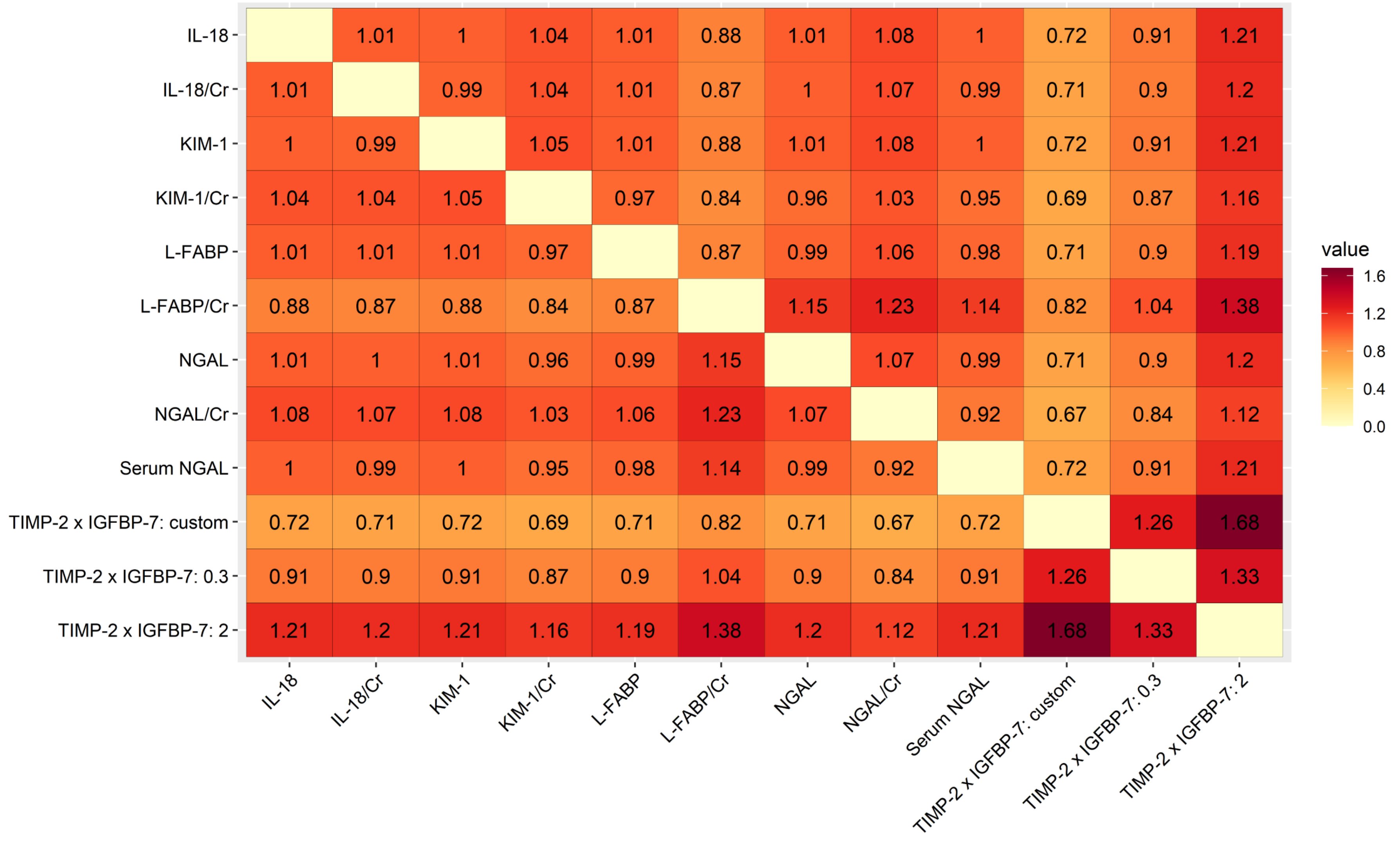


**Abbreviations:** Cr, creatinine; IL-18, interleukin-18; KIM-1, kidney injury molecule-1; L-FABP, liver-type fatty acid binding protein; NGAL, neutrophil gelatinase-associated lipocalin; TIMP-2 x IGFBP-7: tissue inhibitor of metalloproteinases-2 x insulin-like growth factor-binding protein 7.

**Supplemental Figure 15.** **Heatmap plot depicted pairwise comparison (row vs. column) of relative DOR between the biomarkers in the non-sepsis subgroup.** The contents of the diagonal are the values of the relative DOR. Red depicts positive DOR while yellow depicts no correlation. NGAL and NGAL/Cr had the best relative DOR in the biomarkers.


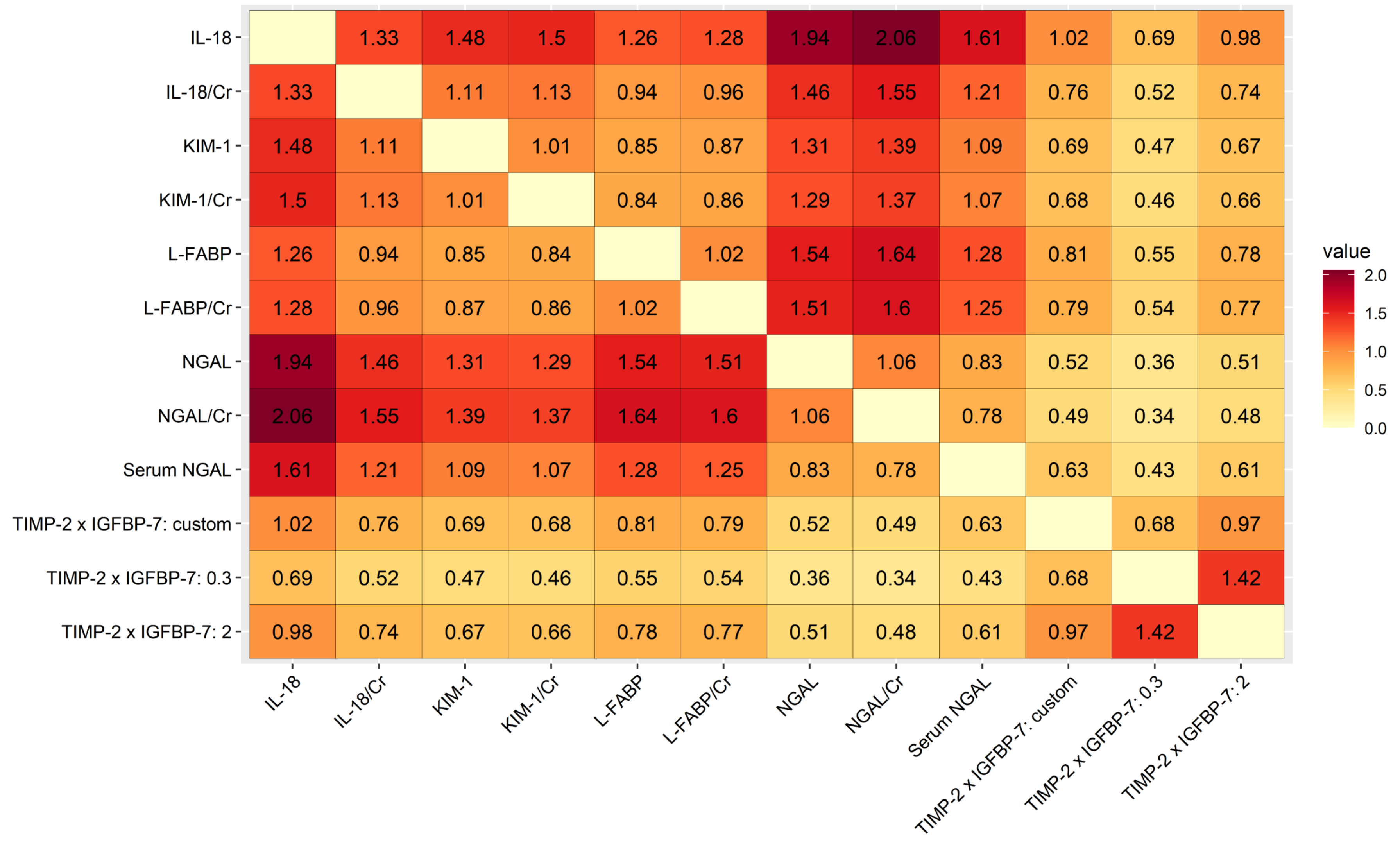


**Abbreviations:** Cr, creatinine; DOR, diagnostic odds-ratio; IL-18, interleukin-18; KIM-1, kidney injury molecule-1; L-FABP, liver-type fatty acid binding protein; NGAL, neutrophil gelatinase-associated lipocalin; TIMP-2 x IGFBP-7: tissue inhibitor of metalloproteinases-2 x insulin-like growth factor-binding protein 7.

**Supplemental Figure 16.** **Heatmap plot depicted pairwise comparison (row vs. column) of relative sensitivity between the markers in the studies using standard AKI criteria (RIFLE/AKIN/KDIGO).** The contents of the diagonal are the values of the relative sensitivity. Red depicts positive sensitivity while yellow depicts no correlation. TIMP-2 x IGFBP-7: custom had the best relative sensitivity in the biomarkers.


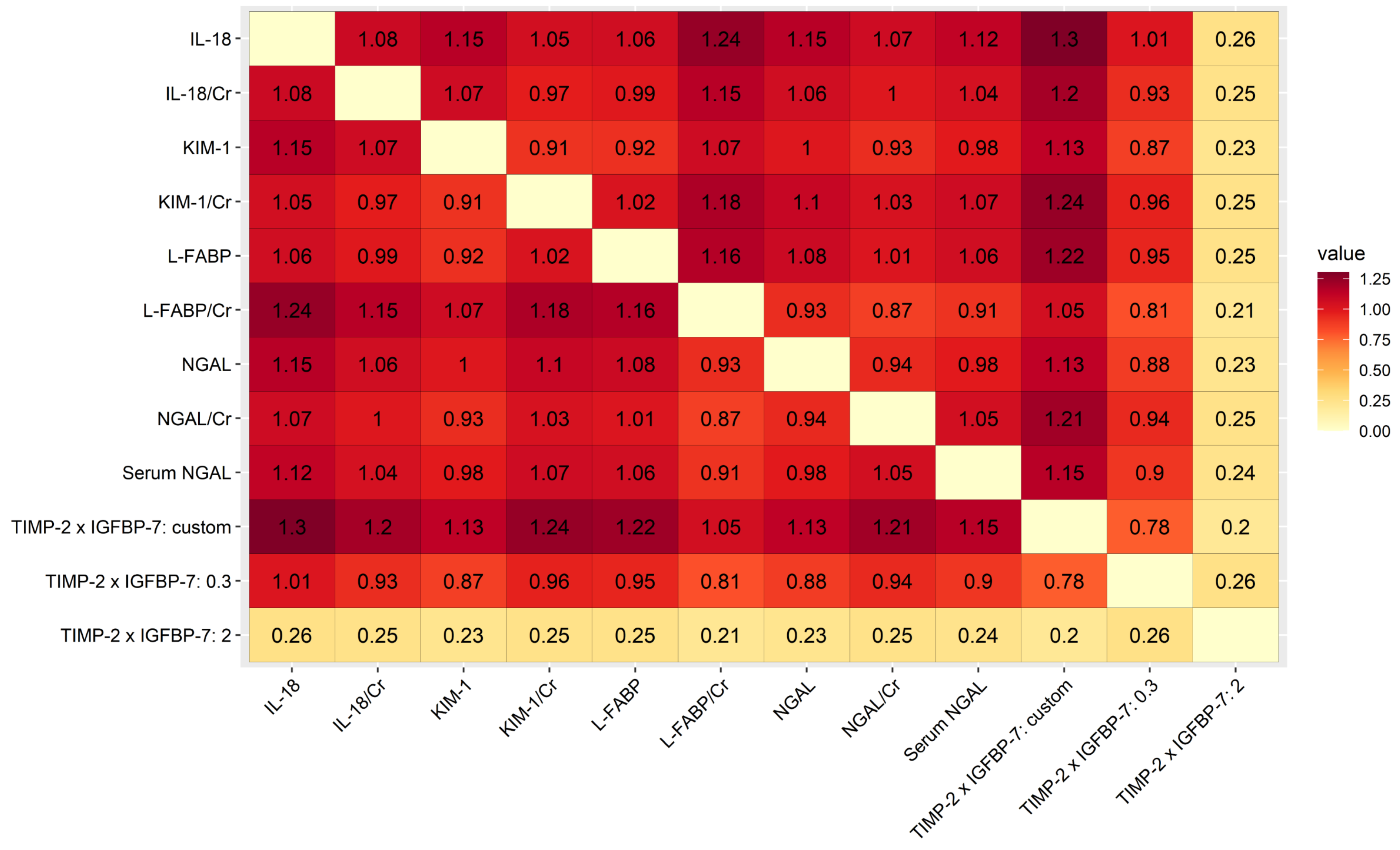


**Abbreviations:** AKI, acute kidney injury; AKIN, Acute Kidney Injury Network; CI, confidence interval; Cr, creatinine; IL-18, interleukin-18; KDIGO, Kidney Disease Improving Global Outcomes; KIM-1, kidney injury molecule-1; L-FABP, liver-type fatty acid binding protein; NGAL, neutrophil gelatinase associated lipocalin; RIFLE, Risk, Injury, Failure, Loss, and End-stage renal disease; TIMP-2 x IGFBP-7, tissue inhibitor of metalloproteinases-2 x insulin-like growth factor binding protein-7.

**Supplemental Figure 17.** **Heatmap plot depicted pairwise comparison (row vs. column) of relative specificity between the markers in the studies using standard AKI criteria (RIFLE/AKIN/KDIGO).** The contents of the diagonal are the values of the relative specificity. Red depicts positive relative specificity while yellow depicts no correlation. TIMP-2 x IGFBP-7: 2 had the best relative specificity in the biomarkers.


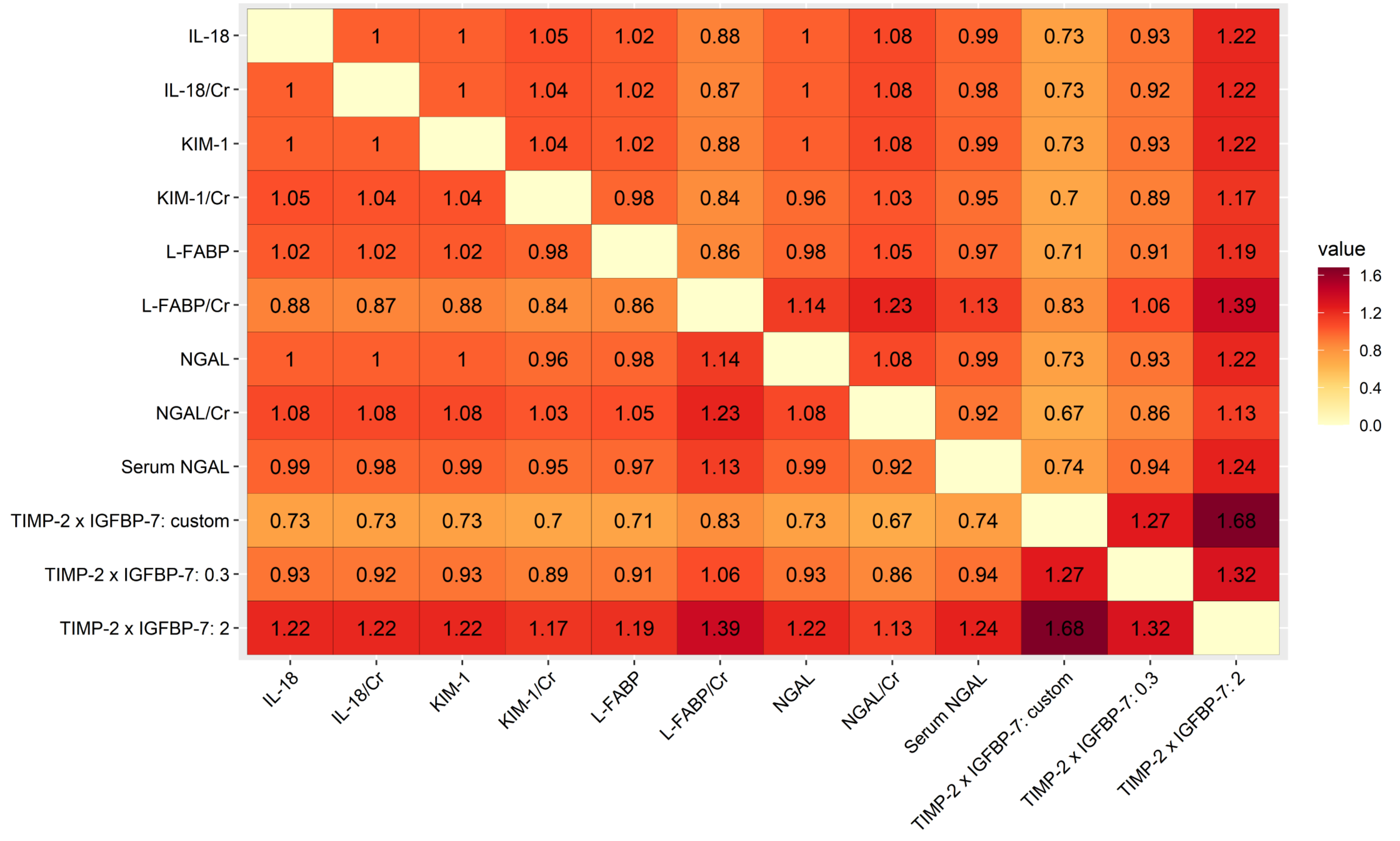


**Abbreviations:** AKI, acute kidney injury; AKIN, Acute Kidney Injury Network; CI, confidence interval; Cr, creatinine; IL-18, interleukin-18; KDIGO, Kidney Disease Improving Global Outcomes; KIM-1, kidney injury molecule-1; L-FABP, liver-type fatty acid binding protein; NGAL, neutrophil gelatinase associated lipocalin; RIFLE, Risk, Injury, Failure, Loss, and End-stage renal disease; TIMP-2 x IGFBP-7, tissue inhibitor of metalloproteinases-2 x insulin-like growth factor binding protein-7.

**Supplemental Figure 18.** **Heatmap plot depicted pairwise comparison (row vs. column) of relative DOR between the markers in the studies using standard AKI criteria (RIFLE/AKIN/KDIGO).** The contents of the diagonal are the values of the relative DOR. Red depicts positive DOR while yellow depicts no correlation. L-FABP/Cr had the best relative DOR in the biomarkers.


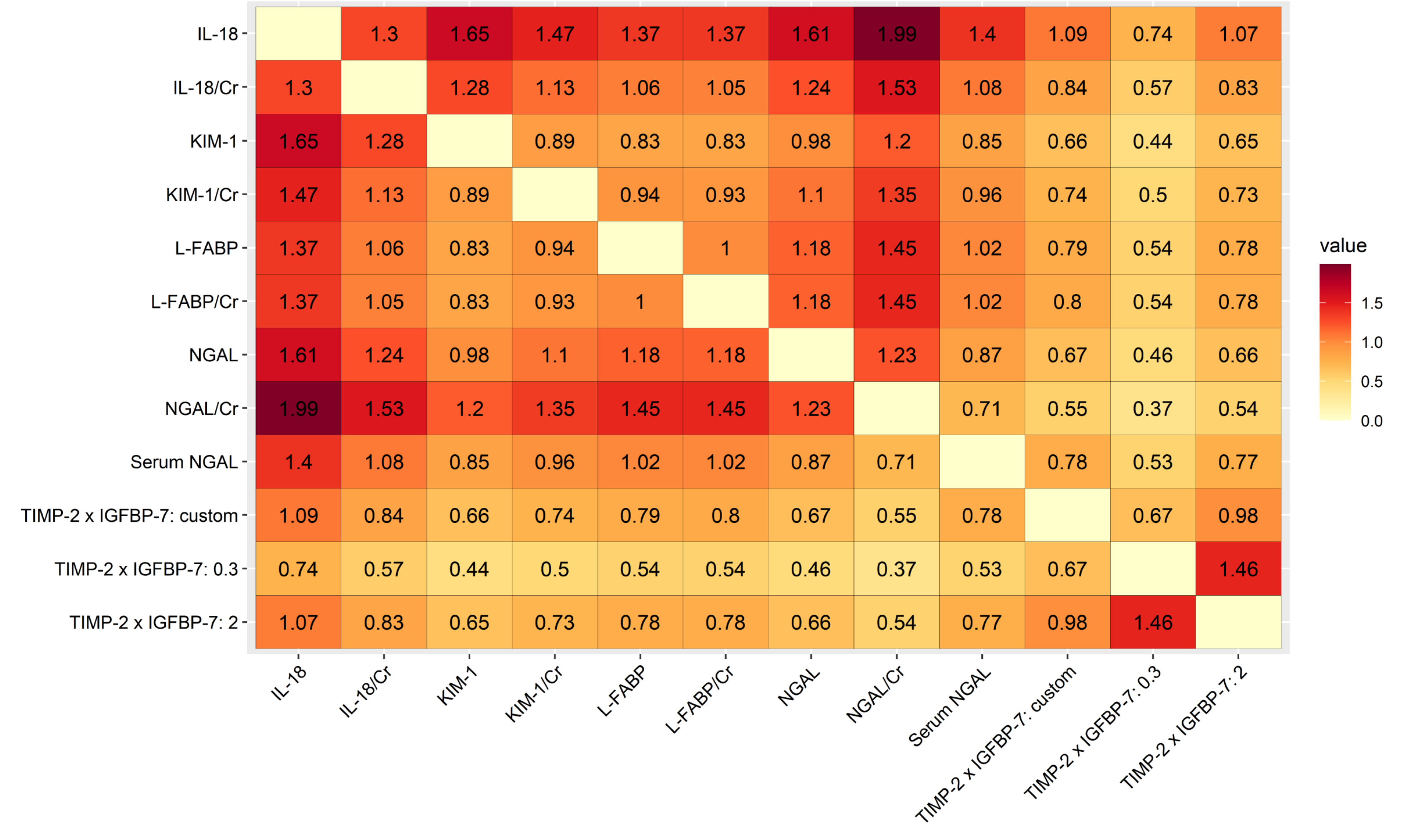


**Abbreviations:** AKI, acute kidney injury; AKIN, Acute Kidney Injury Network; CI, confidence interval; Cr, creatinine; DOR, diagnostic odds ratio; IL-18, interleukin-18; KDIGO, Kidney Disease Improving Global Outcomes; KIM-1, kidney injury molecule-1; L-FABP, liver-type fatty acid binding protein; NGAL, neutrophil gelatinase associated lipocalin; RIFLE, Risk, Injury, Failure, Loss, and End-stage renal disease; TIMP-2 x IGFBP-7, tissue inhibitor of metalloproteinases-2 x insulin-like growth factor binding protein-7.

**Supplemental Figure 19.** **Heatmap plot depicted pairwise comparison (row vs. column) of relative sensitivity between the markers in the studies that did not use UO criteria.** The contents of the diagonal are the values of the relative sensitivity. Red depicts positive sensitivity while yellow depicts no correlation. TIMP-2 x IGFBP-7: custom had the best relative sensitivity in the biomarkers.


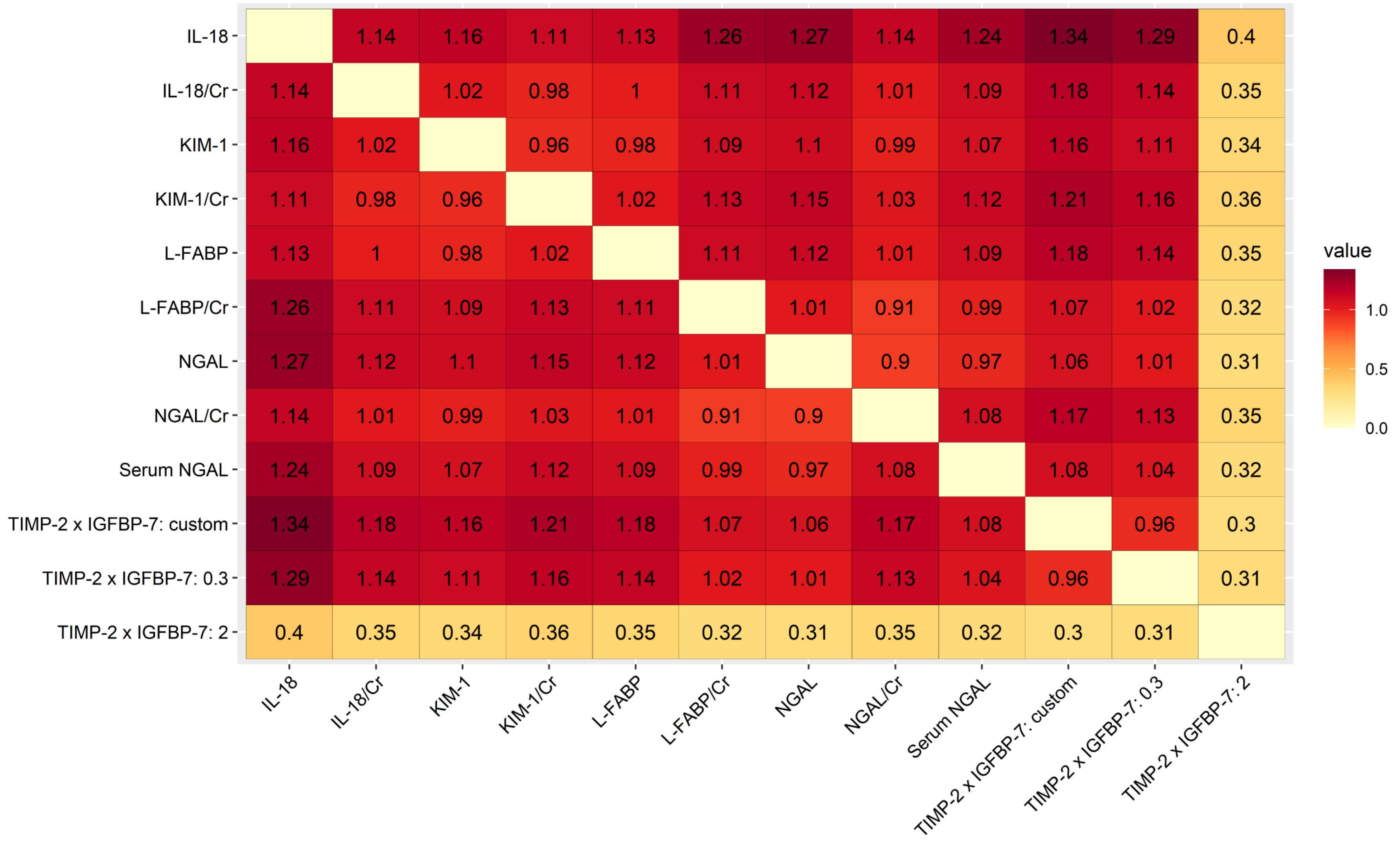


**Abbreviations:** AKI, acute kidney injury; AKIN, Acute Kidney Injury Network; CI, confidence interval; Cr, creatinine; IL-18, interleukin-18; KDIGO, Kidney Disease Improving Global Outcomes; KIM-1, kidney injury molecule-1; L-FABP, liver-type fatty acid binding protein; NGAL, neutrophil gelatinase associated lipocalin; RIFLE, Risk, Injury, Failure, Loss, and End-stage renal disease; TIMP-2 x IGFBP-7, tissue inhibitor of metalloproteinases-2 x insulin-like growth factor binding protein-7; UO, urine output.

**Supplemental Figure 20.** **Heatmap plot depicted pairwise comparison (row vs. column) of relative specificity between the markers in the studies that did note use UO criteria.** The contents of the diagonal are the values of the relative specificity. Red depicts positive specificity while yellow depicts no correlation. TIMP-2 x IGFBP-7: 2 had the best relative specificity in the biomarkers.


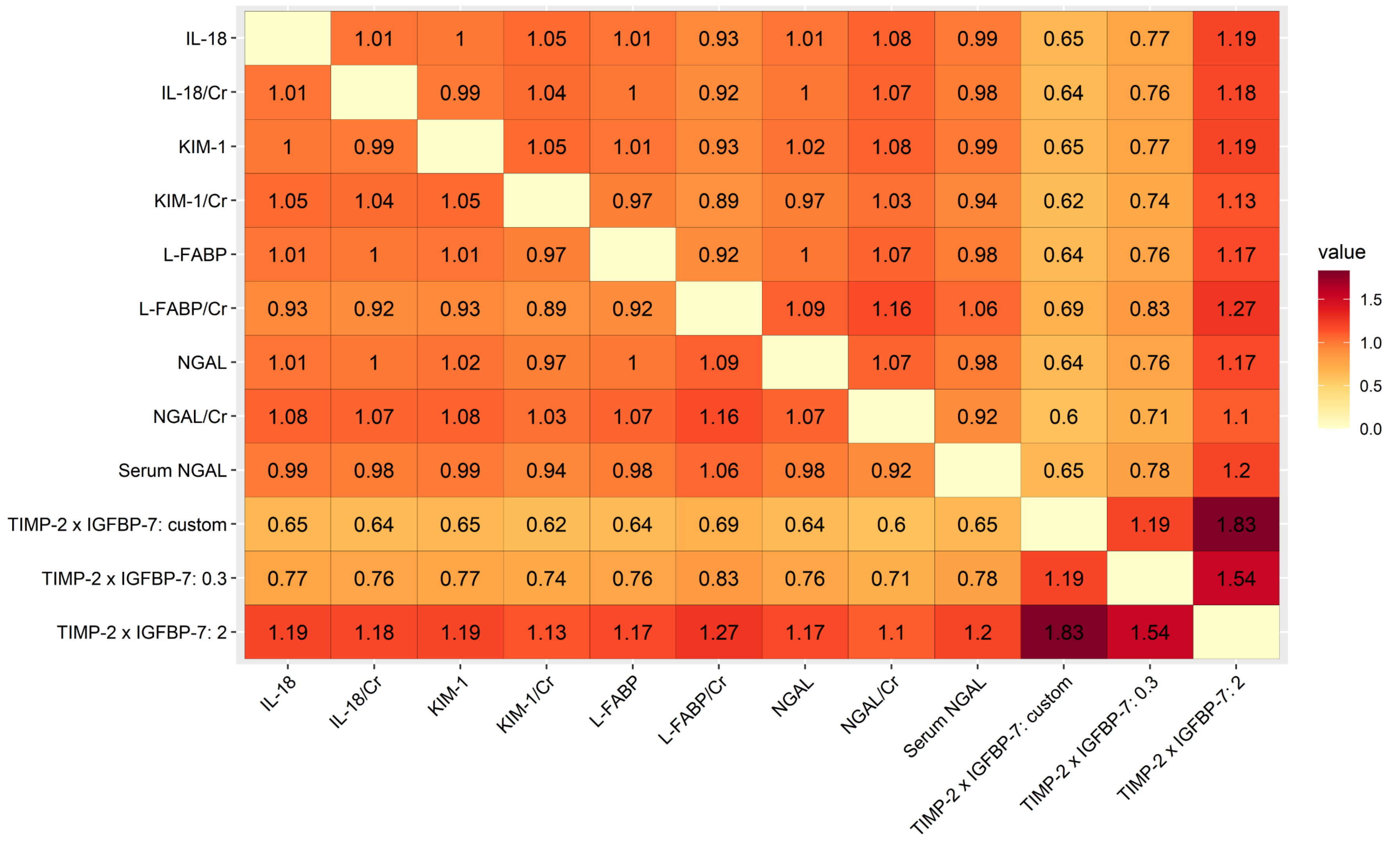


**Abbreviations:** AKI, acute kidney injury; AKIN, Acute Kidney Injury Network; CI, confidence interval; Cr, creatinine; IL-18, interleukin-18; KDIGO, Kidney Disease Improving Global Outcomes; KIM-1, kidney injury molecule-1; L-FABP, liver-type fatty acid binding protein; NGAL, neutrophil gelatinase associated lipocalin; RIFLE, Risk, Injury, Failure, Loss, and End-stage renal disease; TIMP-2 x IGFBP-7, tissue inhibitor of metalloproteinases-2 x insulin-like growth factor binding protein-7; UO, urine output.

**8. Summary of contextual factor data**

In our study, a total 110 articles were included in this meta-analysis.

**For analysis of the clinical use of neutrophil gelatinase associated lipocalin (NGAL) as a biomarker for acute kidney injury (AKI), studies exploring the urine NGAL (n = 35), urine NGAL/urine creatinine (n = 9), and serum or plasma NGAL (n = 40) were included.**

Qian et al. (2019) included 91 patients undergoing cardiac surgery, and found that postoperative urine NGAL collected 2 h after admission to intensive care unit (ICU) to predict AKI had a highest area under the curve of the receiver operating characteristic curve (AUC-ROC) 0.78, (95% confidence interval (CI) 0.68 - 0.88) with the cutoff 4.49 ng/μmol (corrected for urine creatinine excretion).

Prowle et al. (2015) enrolled 93 patients undergoing cardiopulmonary bypass (CPB), and revealed that both postoperative urine NGAL and urine NGAL/creatinine could predict AKI, with AUC-ROC 0.71 and 0.73 respectively.

Lei et al. (2018) included 150 patients with decompensated cirrhosis and healthy controls, and showed that urinary NGAL for diagnosis of AKI secondary to decompensated cirrhosis had sensitivity (SEN) 80.2% and specificity (SPE) 74.9%.

van Wolfswinkel et al. (2016) included 39 patients with imported falciparum malaria, and revealed that urine NGAL had an AUC-ROC of 1.00 to predict AKI.

Srisawat et al. (2015) enrolled 113 patients with confirmed leptospirosis, and showed that urine and plasma NGAL levels had AUC-ROC of 0.91 and 0.92 respectively, to predict leptospirosis-associated AKI.

Zeng et al. (2014) enrolled 199 patients undergoing major surgery, and revealed that urinary NGAL, with an AUC-ROC of 0.83 (95 % CI 0.74 - 0.91) could be used to detect AKI.

Aydogdu et al. (2013) investigated 151 critically ill patients, and showed that urinary NGAL discriminated the diagnosis of AKI significantly (AUC-ROC 0.80) with a threshold value of 29.5 ng/ml (SEN 88%, SPE 73%).

Liu et al. (2013) included 109 patients undergoing open heart surgery, and showed urine NGAL increased at an early stage after surgery. They revealed that AUC-ROC for NGAL was 0.866 at 0 h (SEN 0.769, SPE 0.819, cutoff 131.12 μg/g Ucr) and 0.871 at 2 h (SEN 0.808, SPE 0.831, cutoff 33.73 μg/g Ucr) to predict AKI.

Wagener et al. (2011) included 92 patients undergoing liver transplantation. The AUC-ROC of urinary NGAL/creatinine ratio to predict AKI were 0.800 (95% CI 0.732 - 0.869) 3 h and 0.636 (95% CI 0.551 - 0.720) 18 h after reperfusion.

Makris et al. (2009) studied 31 multi-trauma patients admitted to ICU, and revealed that urinary NGAL at baseline could predict AKI (AUC-ROC 0.977, 95% CI 0.823 - 0.980).

Constantin et al. (2010) investigated 88 patients admitted to ICU, and found that for plasma NGAL to predict AKI using a cutoff of 155 nmol/L, SEN and SPE were 82% and 97% respectively, with the AUC-ROC 0.92 (95% CI 0.852 - 0.972).

Cruz et al. (2010) included 301 adults admitted to ICU, and found that plasma NGAL was a good diagnostic marker for AKI development within the next 48 h (AUC-ROC 0.78, 95% CI 0.65 - 0.90).

de Geus et al. (2011) studied 632 adults admitted to ICU, and found that plasma and urine NGAL at ICU admission were significantly related to AKI severity.

Endre et al. (2011) included 528 patients admitted to ICU, and concluded that the duration of injury and baseline renal function should be considered in evaluating urinary NGAL performance to diagnose AKI.

Breidthardt et al. (2012) studied 207 patients with acute heart failure (AHF), and concluded that plasma NGAL does not adequately predict AKI in patients with AHF.

Camou et al. (2013) studied 50 patients with septic shock, and suggested that plasma NGAL predicted AKI in septic shock at ICU admission.

Doi et al. (2013) investigated 146 adults with scheduled cardiac surgery, and found that increased preoperative plasma NGAL was an independent risk factor for post-cardiac surgery AKI.

Gaipov et al. (2015) studied 60 patients undergoing cardiac surgery, and found that urine NGAL was an independent predictor of development of progressive AKI.

Cuartero et al. (2019) included 100 critically ill patients, and found that whole-blood NGAL at ICU admission was a good stratifier of AKI in these patients.

Khawaja et al. (2019) enrolled 46 patients with sepsis, and found that plasma NGAL was an early predictor of AKI in adults admitted to ICU, and it allowed the diagnosis of AKI 48 h prior to a clinical diagnosis based on RIFLE criteria.

Mosa et al. (2018) analyzed 182 patients using CPB, and found that postoperative serum NGAL was a sensitive marker for AKI.

Sun et al. (2017) investigated 138 patients with scrub typhus, and revealed that serum NGAL might be an additive predictor for scrub typhus-associated AKI.

Ghonemy et al. (2014) enrolled 50 patients receiving cardiac surgery, and showed that plasma NGAL may be considered as an early predictor of AKI after CPB operations.

Padhy et al. (2014) studied 60 patients undergoing percutaneous coronary intervention (PCI), and suggested that serum NGAL may act as an early marker of contrast-induced AKI in these patients.

de Geus et al. (2013) analyzed 663 patients admitted to ICU, and concluded that plasma NGAL similarly predicted AKI in sepsis and non-sepsis.

Haase-Fielitz et al. (2009) included 100 adult cardiac surgical patients, and found that early postoperative plasma NGAL was of good value in identifying patients who developed AKI after cardiac surgery.

Hanson et al. (2011) analyzed 163 adults with severe malaria, and found that in patients with impaired renal function on admission, the urinary NGAL was not superior to the plasma creatinine when used to predict a later requirement for renal replacement therapy (RRT).

Introcaso et al. (2018) included 69 patients who underwent cardiac surgeries. For predicting AKI, post-operative plasma NGAL had an AUC-ROC of 0.71 (95% CI 0.60 - 0.82) with a cut-off 154 ng/mL (SEN 76%, SPE 59%). NGAL after surgery also had a good correlation with the AKI stage severity (p ≤ 0.001).

Kim et al. (2017) studied 167 septic patients, and found that plasma NGAL had an AUC-ROC 0.675 (95% CI 0.599 - 0.746), with cutoff 493 ng/mL (SEN 73.2%, SPE 60.3%), in predicting AKI.

Doi et al. (2011) investigated 339 critically ill adults, and revealed that urinary NGAL had an AUC-ROC 0.695 for AKI detection.

Ferguson et al. (2010) studied 92 patients with AKI and 68 control subjects, and showed that urinary NGAL had an AUC-ROC 0.92 for detecting AKI in hospitalized patients.

Li et al. (2012) studied 25 patients receiving liver transplantation, and urinary NGAL before surgery and at 2, 4, 6, 12, 24, 48, 72 and 120 h after the anhepatic phase were tested. For predicting AKI, the AUC-ROC of urinary NGAL were 0.766, 0.773, and 0.773 at 2, 4 and 6 h respectively.

Matsui et al. (2012) studied 85 adults undergoing cardiac surgery, and found that urinary liver-type fatty acid binding protein (L-FABP) was a better biomarker for early detection and a good early predictor of the onset of AKI, than the urinary NGAL.

Liangos et al. (2009) enrolled 103 patients undergoing cardiac surgery, and showed that the 2-h post-CPB urinary NGAL was not feasible for early detection of AKI (AUC-ROC 0.50).

Nickolas et al. (2012) enrolled 1635 emergency department patients at the time of admission, and found that urinary NGAL had an AUC-ROC 0.81 to distinguish intrinsic AKI from other diagnosis.

Vaidya et al. (2008) investigated 102 patients with AKI and 102 patients without AKI, and revealed that urinary NGAL had and AUC-ROC 0.89 for detecting AKI.

Nickolas et al. (2008) studied 635 patients admitted to the hospital, and found that urinary NGAL had an AUC-ROC 0.948 to detect AKI.

Cho et al. (2013) enrolled 145 patients admitted to ICU, and showed the diagnostic performance for AKI assessed by AUC-ROC was 0.773 for urinary NGAL.

Park et al. (2019) studied 140 patients with sepsis in the emergency department, and revealed the AUC-ROC of urinary NGAL was 0.820 for predicting AKI.

Perry et al. (2010) enrolled 879 patients after coronary artery bypass graft (CABG) surgery, and showed the AUC-ROC of post-operative plasma NGAL was 0.64 for predicting AKI.

Shapiro et al. (2010) investigated 661 Emergency Department patients with suspected sepsis, and found that plasma NGAL concentrations greater than 150 ng/mL were 96% sensitive (95% CI 79 - 100%) and 51% (95% CI 47 - 55%) specific for AKI.

Thanakitcharu et al. (2014) included 130 adults undergoing open cardiac surgery. They concluded that using cut-off value 11.3 ng/mL, the urinary NGAL at 3 h after surgery predicted AKI with SEN 72% and SPE 60%.

Valette et al. (2013) analyzed 98 critically ill patients, and found that the discriminative value of plasma NGAL to predict contrast-induced AKI and mortality was poor.

Varela et al. (2015) investigated 66 adults undergoing cardiac surgery, and revealed that urine NGAL at 6-h time point had an AUC-ROC 0.77 (SEN: 87.5%, SPE: 65.9 %) for predicting post-operative AKI.

Chen et al. (2012) investigated 150 patients in coronary care unit (CCU), and showed that serum and urine NGAL had the AUC-ROC 0.828 and 0.796 respectively for AKI prediction.

Nisula et al. (2014) enrolled 1042 adult ICU patients, and concluded that urine NGAL did not provide additional predictive value for AKI in critically ill patients.

Maisel et al. (2016) studied 927 patients presenting with AHF requiring intravenous diuretics, and concluded that the plasma NGAL did not add significant diagnostic utility over the first creatinine.

Matsa et al. (2014) included 194 critically ill patients, and concluded that both plasma and urine NGAL measured at admission could predict AKI occurrence up to 72 h post-ICU admission, with fair performance.

Munir et al. (2013) analyzed 88 patients undergoing CPB surgery, and found that urine NGAL at a cutoff value of 87 ng/ml had an AUC-ROC 0.91 (95% CI 0.83 - 0.96) with SEN 90.9% (95% CI 58.7 - 98.5) and SPE 98.7% (95% CI 92.9 - 99.8).

Onk et al. (2016) enrolled 375 patients who underwent CABG surgery, and concluded that the early postoperative NGAL results were highly specific for the early recognition of AKI.

Ralib et al. (2017) recruited 225 ICU patients with sepsis and systemic inflammatory response syndrome, and found that the AUC-ROC of plasma NGAL for predicting AKI was 0.81 (95% CI 0.74 - 0.87).

Yang et al. (2016) enrolled 103 patients with acute decompensated heart failure admitted to CCU, and revealed that the discrimination of urine NGAL on the first day of CCU admission in predicting intrinsic AKI was good (AUC-ROC: 0.813, 95% CI 0.732 - 0.894).

Ueta et al. (2014) studied 42 patients receiving endovascular stent graft repair of aortic aneurysm, and found that urinary NGAL/creatinine was a potentially useful early biomarker for AKI after stent graft repair of aortic aneurysm.

Chang et al. (2015) included 147 CCU patients, and found that urinary NGAL displayed excellent AUC-ROC (0.918, 95% CI 0.866 - 0.970) for predicting intrinsic AKI.

Hjortrup et al. (2014) enrolled 222 ICU patients with severe sepsis, and showed that the AUC-ROC of plasma and urine NGAL for predicting AKI were 0.66 (95% CI 0.54 - 0.77) and 0.71 (95% CI 0.59 - 0.82), respectively.

Chen et al. (2020) investigated 269 CCU patients, and revealed that the AUC-ROC of serum and urine NGAL for predicting acute kidney disease were 0.63 and 0.66 (95% CI 0.563 - 0.697 and 0.591 - 0.730, both p < 0.001) respectively.

Torregrosa et al. (2014) enrolled 193 ICU patients with acute coronary syndrome (ACS) or heart failure undergoing coronary angiography or cardiac surgery, and showed that the AUC-ROC of urine NGAL for predicting AKI was 0.96 for the angiography group and 0.92 for the cardiac surgery group.

Torregrosa et al. (2012) investigated 135 patients admitted to the ICU after heart angiography or heart surgery in patients with ACS or AHF, and concluded that urinary NGAL is an early marker of AKI in these patients, with a higher predictive value than cystatin C or interleukin-18 (IL-18).

Parikh et al. (2011) enrolled 1219 adults undergoing cardiac surgery, and showed that the AUC-ROC of urine and plasma NGAL for predicting AKI were 0.67 and 0.70 respectively.

Haase-Fielitz et al. (2009) analyzed 100 adult cardiac surgery patients, and found that the discriminatory ability of postoperative plasma NGAL for predicting AKI also increased with increasing RIFLE classes (AUC-ROC R: 0.72, I: 0.79, F: 0.80) or AKIN stages (AUC-ROC 1: 0.75, 2: 0.78, 3: 0.81).

Imoto et al. (2021) included 106 patients admitted to ICU, and found that in ROC analysis on days 1–3 after admission, urinary NGAL levels can be used as biomarkers for the diagnosis of AKI, particularly AKI stage 3.

Szymanowicz et al. (2021) enrolled 114 adult patients undergoing cardiac surgery using CPB, and identified preoperative NGAL ≥ 91.5 ng/mL and postoperative NGAL ≥ 140.5 ng/mL as independent and significant cardiac surgery-associated AKI predictors.

Zhen et al. (2021) investigated 172 patients with ACS admitted to CCU, and revealed that plasma NGAL could discriminate the development of AKI with an AUC-ROC of 0.864.

Obata et al. (2021) explored 64 patients undergoing open surgical repair of an abdominal aortic aneurysm, and found that urinary NGAL levels 2 h after aortic cross-clamping may be useful for early detection of AKI.

Qiu et al. (2021) analyzed 90 critically ill patients with sepsis, and revealed that urine NGAL was a predictive indicator of AKI occurrence (AUC-ROC 0.729, 95% CI 0.625 - 0.818).

Shakked et al. (2021) enrolled 52 patients presenting to the emergency department with laboratory-confirmed Coronavirus disease-2019 (COVID-19), and revealed that serum NGAL displayed acceptable performance for predicting AKI (AUC-ROC 0.81) and need for RRT (AUC-ROC 0.87).

Ergun et al. (2021) included 60 geriatric patients undergoing laparotomic oncological surgery, and found that high NGAL levels at 6 and 24 h after surgery seem to be highly predictive of AKI development. They also reported that at 6 h, a plasma NGAL level greater than 71.8 ng/mL has SEN 85% and SPE 81% in predicting subsequent AKI development.

Pei et al. (2022) enrolled 162 patients with sepsis, and showed that the AUC-ROC of serum NGAL for predicting AKI after sepsis was 0.620 (95% CI 0.529 - 0.711).

Jahaj et al. (2021) analyzed 266 initially nonseptic patients admitted to ICU, and demonstrated that NGAL was shown to be more accurate in predicting AKI development than creatinine. They also reported that NGAL levels were associated with an increased risk of AKI development.

Garms et al. (2021) studied 94 patients receiving vancomycin and admitted to wards, and Logistic regression identified urinary NGAL levels between 96 and 144 h (OR 1.123, 95% CI 1.096 - 1.290, p < 0.03) as a predictor of AKI.

Guray et al. (2021) enrolled 84 patients with left ventricular systolic dysfunction patients referred for coronary angiography, and found that plasma NGAL 24th h levels after the index procedure were found to be an independent and significant predictor of contrast-induced nephropathy in multivariate analysis.

Tan et al. (2022) included 157 patients with urosepsis after ureteroscopic lithotripsy, and revealed that the combined detection of urine IL-18, NGAL and KIM-1 at 12 h had a larger AUC-ROC than a single marker (0.997, 95% CI 0.991 - 0.998) with SEN 98.2% and SPE 96.7%.

Sahu et al. (2022) investigated 212 patients undergoing PCI. For predicting the occurrence of contrast-induced nephropathy, the AUC-ROC of plasma NGAL was 0.878 (95% CI: 0.801 - 0.955) with SEN 68% and SPE 95.2% at a cutoff of 256.5 ng/mL.

**We included 18 studies exploring the urinary tissue inhibitor of metalloproteinases-2 x insulin-like growth factor binding protein-7 (TIMP-2 × IGFBP-7) as a biomarker of AKI.**

Ferrari et al. (2019) included 442 critically ill adults. For predicting AKI, urinary TIMP-2 × IGFBP-7 at ICU admission had AUC-ROC 0.70 (95% CI 0.65 - 0.76) within 48 h, and AUC-ROC 0.68 (95% CI 0.63 - 0.73) within 7 days.

Xie et al. (2019) analyzed 719 patients admitted to ICU, and found that the urinary TIMP-2 × IGFBP-7 values could serve to identify patients with AKI at increased risk for adverse outcomes in the ICU.

Oezkur et al. (2017) studied 150 patients undergoing cardiac surgery, and found that early detection of elevated urinary TIMP-2 × IGFBP-7 at ICU admission was strongly predictive for postoperative AKI.

Wang et al. (2017) enrolled 57 cardiac surgery patients, and found that urinary TIMP-2 × IGFBP-7 4 h after postoperative ICU admission identified patients at risk for developing AKI following cardiac surgery.

Finge et al. (2017) included 93 patients undergoing cardiac surgery with CPB, and concluded that the urinary TIMP-2 × IGFBP-7 could not accurately predict the occurrence of postoperative AKI.

Cuartero et al. (2017) studied 98 patients admitted to ICU, and found that the urinary TIMP-2 × IGFBP-7 was an early predictor of AKI in ICU patients regardless of sepsis.

Meersch et al. (2014) studied 50 patients undergoing cardiac surgery with CPB, and found that the urinary TIMP-2 × IGFBP-7 served as a sensitive and specific biomarker to predict AKI early after cardiac surgery and to predict renal recovery.

Dusse et al. (2016) included 40 patients undergoing transapical and transaortic aortic valve implantation, and showed that the urinary TIMP-2 × IGFBP-7 provided an excellent diagnostic accuracy in predicting AKI.

Gunnerson et al. (2016) studied 375 adult surgical patients admitted to ICU, and found that a single urinary TIMP-2 × IGFBP-7 accurately identified patients at risk for developing AKI within the ensuing 12 hrs.

Wetz et al. (2015) enrolled 42 patients undergoing CABG surgery, and showed that urinary TIMP-2 × IGFBP-7 could identify patients at increased risk of AKI after cardiac surgery within 24 h after operation.

Kimmel et al. (2016) included 362 patients from the emergency department, and concluded that the urinary TIMP-2 × IGFBP-7 may enable more effective use of urine microscopy in these patients.

Pilarczyk et al. (2015) analyzed 60 patients undergoing isolated on‐pump CABG, and concluded that the urinary TIMP-2 × IGFBP-7 represented a sensitive and specific biomarker to predict moderate to severe AKI very early after operation.

Hoste et al. (2014) analyzed 153 critically ill patients, and suggested that urinary TIMP-2 × IGFBP-7 values of 0.3 or greater identified patients at high risk and those >2 at highest risk for AKI.

Cummings et al. (2018) investigated 400 patients undergoing cardiac surgery, and found that intraoperative elevations of urinary TIMP-2 × IGFBP-7 could predict moderate-severe AKI.

Waskowski et al. (2021) enrolled 93 adults with emergency/elective abdominal aortic repair, and showed that the previously proposed cutoff levels of TIMP-2 × IGFBP-7 at 0.3 and 2 showed moderate SEN/SPE (0.58/0.58 and 0.16/0.98, respectively).

Pilarczyk et al. (2022) studied 101 patients undergoing thoracic aortic surgery with moderate hypothermic circulatory arrest, and found that TIMP-2 × IGFBP-7 4 h after surgery was able to predict postoperative AKI (AUC-ROC 0.724, p = 0.020).

Irqsusi et al. (2021) included 50 patients undergoing cardiac surgery with the use of CPB, and found that predictive value for TIMP-2 × IGFBP-7 was shown at 0 and 24 h after admission to ICU. At 0 h, SEN was 84.6% and SPE was 55.6% for an ideal calculated cutoff at 0.07. After 24 h, the ideal cutoff amounted to 0.35 with a SEN 53.8% and SPE 88.2%. The AUC-ROC were 0.725 and 0.718 at 0 h and after 24 h respectively.

Lakhal et al. (2021) included 65 patients over 75 years-old undergoing aortic valve replacement with CPB, and found that AUC-ROC of TIMP-2 × IGFBP-7 (6 h after CPB) was 0.64 (95% CI 0.51 - 0.76) for the detection of cardiac surgery-associated AKI.

**We included 10 articles exploring urine L-FABP and 8 articles exploring urine L-FABP/creatinine.**

Zeng et al. (2014) enrolled 199 patients undergoing major surgery, and found that the AUC-ROC of L-FABP 4 h after surgery for predicting AKI was 0.85.

Liu et al. (2013) included 109 patients undergoing open heart surgery, and revealed that the AUC-ROC for L-FABP was 0.844 (SEN 0.846, SPE 0.819, cut-off 2226.50 μg/g Ucr) at 0 h and 0.832 at 2 h (SEN 0.808, SPE 0.747, cut-off 673.09 μg/g Ucr).

Katagiri et al. (2012) studied 77 patients undergoing cardiac surgery. For predicting AKI, urine L-FABP at 4 h had an AUC-ROC 0.72, with high SEN.

Doi et al. (2011) included 339 critically ill adults, and found that urinary L-FABP detected AKI better than the other biomarkers did (AUC-ROC for L-FABP was 0.75).

Ferguson et al. (2010) included 92 patients with established AKI and 68 control subjects, and found that the diagnostic performance of urinary L-FABP for AKI, assessed by the AUC-ROC, was 0.93 in hospitalized patients.

Li et al. (2012) studied 25 patients receiving liver transplantation, and urinary L-FABP before surgery and at 2, 4, 6, 12, 24, 48, 72 and 120 h after the anhepatic phase were tested. For predicting AKI, The AUC-ROC of L-FABP at 4 h was 0.760.

Manabe et al. (2012) investigated 220 patients with chronic kidney disease who underwent elective catheterization, and revealed that urinary L-FABP level was useful for predicting the onset of contrast-induced AKI before contrast medium exposure.

Matsui et al. (2012) investigated 85 patients receiving cardiac surgery, and concluded that urinary L-FABP was a useful biomarker for early detection of AKI and was a good early predictor of the onset of AKI.

Nicolas et al. (2013) studied 1635 unselected emergency department patients at the time of hospital admission using biomarkers to predict intrinsic AKI. AUC-ROC analyses indicated fair discriminatory ability for urinary L-FABP (AUC-ROC: 0.70, 95% CI 0.65 - 0.76).

Cho et al. (2013) included 145 critically ill patients, and found that urinary L-FABP seemed to be promising both for the diagnosis of AKI and for the prediction of prognosis in heterogeneous ICU patients.

Ueta et al. (2014) studied 42 patients receiving endovascular stent graft repair of aortic aneurysm, and found that the AUC-ROC for prediction of AKI at 2 to 6 h after stent graft repair of aortic aneurysm of urinary L-FABP was 0.87 (cutoff 25.1 ng/mL, SEN 0.83, SPE 0.83).

Torregrosa et al. (2014) investigated 144 patients with ACS or heart failure undergoing coronary angiography, and found that L-FABP did not significantly predict AKI in the group of angiography, but was a significant predictor of AKI in the group of cardiac surgery (AUC-ROC: 0.743; 95 % CI 0.588 - 0.898; p = 0.015).

Matsui et al. (2011) studied 25 patients admitted to ICU, and suggested that L-FABP was a useful biomarker for early detection of AKI.

Prowle et al.(2015) enrolled 93 patients undergoing CPB, and revealed that L-FABP could predict AKI, with AUC-ROC 0.69.

Lee at al. (2021) enrolled 144 patients who received cardiovascular surgery, and showed that the AUC-ROC of urinary L-FABP in predicting postoperative AKI within 7 days was 0.720 at 16 to 18 h postoperatively.

Obata et al. (2021) studied 64 patients undergoing open surgical repair of an abdominal aortic aneurysm, and found that urinary L-FABP levels 2 h after aortic cross-clamping may be useful for early detection of AKI.

Okuda et al. (2022) analyzed 48 patients undergoing emergency laparotomy, and found that AUC-ROC of urinary L-FABP was greater than 0.8 perioperatively, which was larger than that of other biomarkers throughout the study period.

**There were 13 articles exploring urine kidney injury molecule-1 (KIM-1) and 6 articles exploring urine KIM-1/creatinine included in our analysis.**

Lei et al. (2018) enrolled 150 patients with decompensated cirrhosis and healthy controls, and showed that urinary KIM-1 for diagnosis of AKI secondary to decompensated cirrhosis had an AUC-ROC 0.843 (95% CI 0.736 - 0.850, p < 0.001).

van Wolfswinkel et al. (2016) included 39 patients with imported falciparum malaria, and found that urine KIM-1 had a good AUC-ROC (0.87, 95% CI 0.75 - 0.99) for predicting AKI.

Endre et al. (2011) included 528 patients admitted to ICU, and concluded that the duration of injury and baseline renal function should be considered in evaluating the performance of urine KIM-1 to diagnose AKI.

Sun et al. (2017) included 145 patients with scrub typhus, and found that both serum KIM-1 and urine KIM-1/creatinine were not significant predictors of AKI by multivariate logistic regression.

Ferguson et al. (2010) studied 92 patients with AKI and 68 control subjects, and showed that urinary KIM-1 had an AUC-ROC 0.89 (95% CI 0.82 - 0.94) for detecting AKI in hospitalized patients.

Khreba et al. (2019) enrolled 45 patients receiving CPB in open heart surgery, and concluded that urinary KIM-1 could be used as simple noninvasive and specific biomarker for early diagnosis of AKI.

Tu et al. (2014) investigated 150 septic patients, and found that KIM-1 was useful as an early biomarker in the diagnosis of septic AKI. Further, persistent elevation of urinary KIM-1 may be associated with poor prognosis.

Han et al. (2009) studied 90 adults undergoing cardiac surgery, and found that the AUC-ROC for KIM-1 to predict AKI immediately and 3 h after operation were 0.68 and 0.65.

Liangos et al. (2009) included 103 patients undergoing cardiac surgery with CPB. For predicting AKI, the urinary KIM-1 achieved the highest AUC-ROC 0.78, with 95% CI 0.64 - 0.91.

Naggar et al. (2012) investigated 20 critically-ill patients and 20 healthy controls, and concluded that urinary KIM-1 was a reliable early marker for AKI with excellent SEN and SPE.

Nicholas et al. (2012) studied 1,635 unselected emergency department patients at the time of hospital admission, and found that urinary KIM-1 predicted a composite outcome of dialysis initiation or death during hospitalization.

Vaidya et al. (2008) analyzed 102 patients with AKI and 102 patients without AKI, and found that urinary KIM-1 performed well in differentiating between patients with and without AKI with AUC-ROC 0.93 (95% CI 0.88 - 0.96).

Yang et al. (2016) investigated 103 patients with acute decompensated heart failure, and concluded that combinations of Cystatin-C and KIM-1 were effective clinical models for predicting AKI in these patients.

Wybraniec et al. (2017) enrolled 95 patients with coronary artery disease subject to elective or urgent coronary angiography/PCI, ROC analysis denoted that post-procedural KIM-1 at 6 h >0.425ng/mg (AUC-ROC 0.81, p = 0.001) predicted contrast-induced AKI onset.

Sinkala et al. (2016) concluded that KIM‐1 performance was overall unsatisfactory in the diagnosis of kidney disease.

Torregrosa et al. (2014) investigated 144 patients with ACS or heart failure undergoing coronary angiography. For predicting AKI, the AUC-ROC for urinary KIM-1 12 h after intervention was 0.713 in the coronary angiography group, and 0.716 in the cardiac surgery group, respectively.

Tekce et al. (2014) analyzed 22 patients on cisplatin treatment, and concluded that urinary KIM-1 may predict cisplatin-induced AKI in early stages with high SEN and SPE.

Tan et al. (2022) included 157 patients with urosepsis after ureteroscopic lithotripsy, and revealed that the combined detection of urine IL-18, NGAL and KIM-1 at 12 h had a larger AUC-ROC than a single marker (0.997, 95% CI 0.991 - 0.998) with SEN 98.2% and SPE 96.7%.

**There are 12 articles exploring urine IL-18 and 3 articles exploring urine IL-18/creatinine included in our study.**

Endre et al. (2011) included 528 patients admitted to ICU, and concluded that the duration of injury and baseline renal function should be considered in evaluating the performance of urine IL-18/creatinine to diagnose AKI.

Ferguson et al. (2010) included 92 patients with established AKI and 68 control subjects, and found that the diagnostic performance of urinary IL-18/creatinine for AKI, assessed by the AUC-ROC, was 0.83 in hospitalized patients.

Parikh et al. (2005) performed a nested case-control study including 52 case patients with acute respiratory distress syndrome (ARDS) and 86 control patients, and found that urine IL-18 demonstrates an AUC-ROC of 73% to predict AKI in the next 24 h. They concluded that urinary IL-18 could be used for the early diagnosis of AKI, and it also predicted the mortality of patients who have ARDS in ICU.

Parikh et al. (2004) studied urinary IL-18 in 72 patients including healthy controls, patients with different forms of AKI, and patients with other renal diseases. They found that IL-18 levels are elevated in urine in patients with acute tubular necrosis (ATN) and delayed graft function compared with other renal diseases, and concluded that urinary IL-18 may serve as a marker for proximal tubular injury in ATN.

Liangos et al. (2009) included 103 patients undergoing cardiac surgery with CPB. For predicting AKI, the urinary IL-18 had an AUC-ROC 0.66, with 95% CI 0.49 - 0.83.

Nicolas et al. (2012) studied 1635 unselected emergency department patients at the time of hospital admission using biomarkers to predict intrinsic AKI. AUC-ROC analyses indicated poor discriminatory ability for urinary IL-18 (AUC-ROC: 0.64, 95% CI 0.57 - 0.70).

Vaidya et al. (2008) analyzed 102 patients with AKI and 102 patients without AKI, and found that urinary IL-18 performed well in differentiating between patients with and without AKI with AUC-ROC 0.83 (95% CI 0.77 - 0.88).

Nisula et al. (2015) studied 1439 critically ill patients, and concluded that IL-18 had poor-to-moderate ability to predict AKI, RRT, or 90-day mortality in critically ill patients.

Chen et al. (2012) investigated 150 patients in the CCU. The AUC-ROC of the urinary IL-18 on the first day of CCU admission in predicting AKI was 0.621(95% CI 0.504 - 0.732, p = 0.032), and in predicting 6-month mortality was 0.755 (95% CI 0.604 - 0.906, p = 0.001).

Torregrosa et al. (2012) studied 135 patients admitted to the ICU after heart angiography or heart surgery in patients with ACS or AHF. The SEN and SPE of IL-18 for patients receiving heart surgery in predicting AKI were 64.0 and 60.0%, while the SEN and SPE of IL-18 for patients with ACS or AHF in predicting AKI were 67.0 and 73.0%.

Parikh et al. (2011) investigated 1219 adults undergoing cardiac surgery, and concluded that urine IL-18, urine NGAL, and plasma NGAL associated with subsequent AKI and poor outcomes among adults undergoing cardiac surgery.

Wang et al. (2017) enrolled 103 patients undergoing CPB, and revealed that urinary IL-18 in patients at 2 h after CPB had an AUC-ROC 0.905, with the cutoff value 1.6 μg/L, the SEN and SPE were 90.91 and 91.36%, respectively. They concluded that after CPB, the level of urinary IL-18 showed a more promising diagnostic value than serum creatinine and urinary NGAL in early diagnosis of AKI.

Chen et al. (2020) enrolled 269 patients admitted to CCU and reported that AKI biomarkers can predict AKD in CCU patients. About the predictive performance of IL-18/Cr for the occurrence of AKI, the SEN and SPE were 56.5 and 65.1%, respectively.

Tan et al. (2022) included 157 patients with urosepsis after ureteroscopic lithotripsy, and revealed that the combined detection of urine IL-18, NGAL and KIM-1 at 12 h had a larger AUC-ROC than a single marker (0.997, 95% CI 0.991 - 0.998) with SEN 98.2% and SPE 96.7%.

**9. PROSPERO protocol registration**

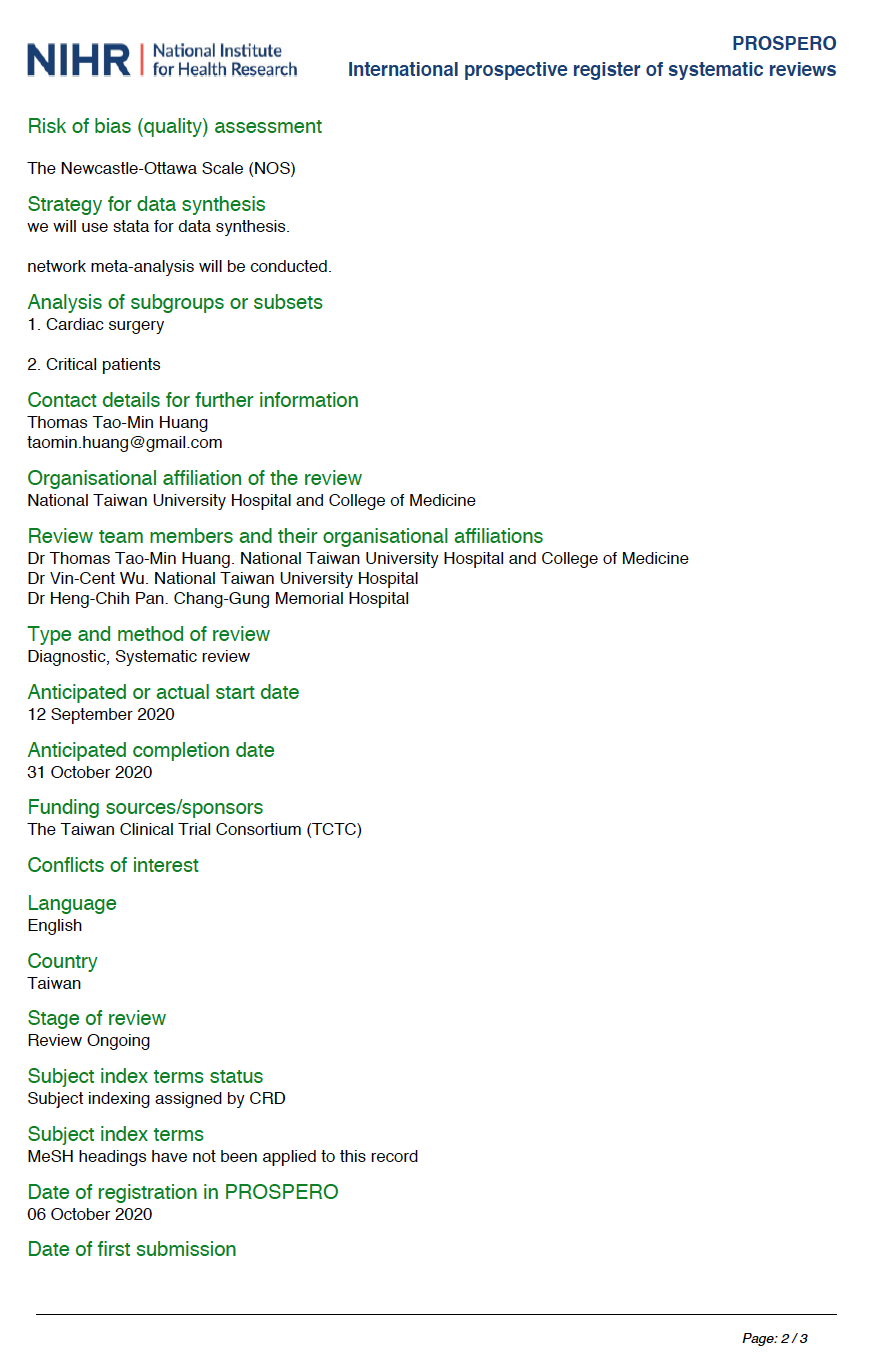


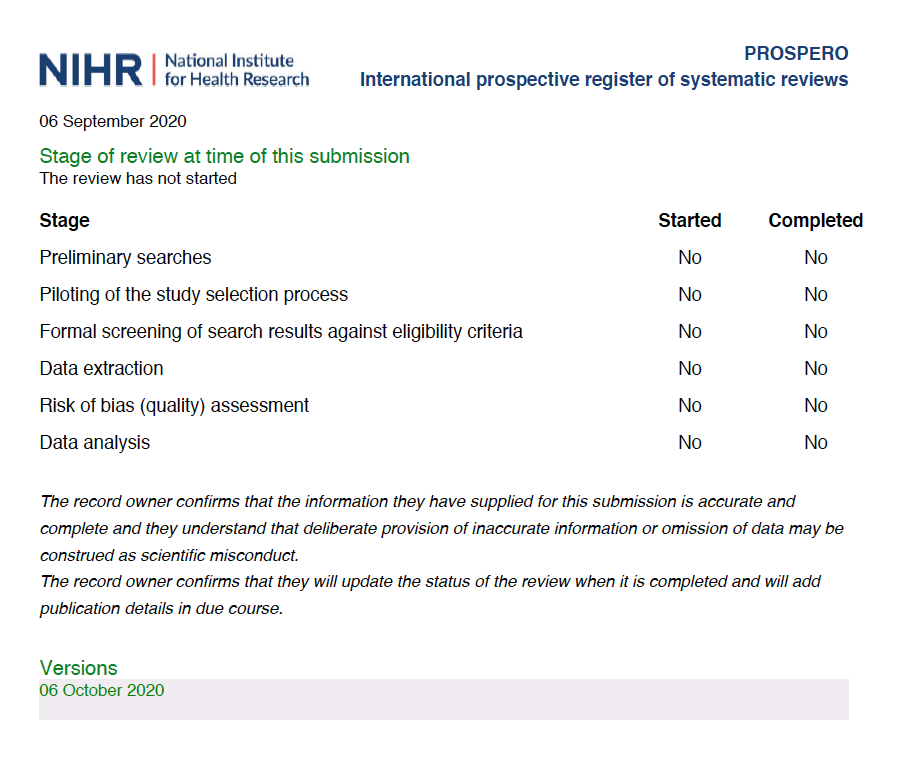


**10. The GRADE results**

as ****
